# Supplementary material for: Spatio-temporal analysis of prostate tumors in situ suggests pre-existence of treatment-resistant clones
Source: Nat Commun. 2022 Sep 17;13:5475. doi: 10.1038/s41467-022-33069-3 (PMC9482614; doi:10.1038/s41467-022-33069-3)
Supplement: Supplementary file 1 — Supplementary Information [file 41467_2022_33069_MOESM1_ESM.pdf]

Spatio-temporal analysis of prostate tumors in situ suggests pre-existence of treatment-resistant clones

Marklund et al.

Supplementary information

Supplementary Table 1: Spearman's rank correlation coefficient between assessed intensity of nuclear AR and repair proteins Ku70 and P-DNA-PKcs. Immunostaining from the fluorescent-labeled AR to the DNA-repair proteins from all individual epithelial cell nuclei across the tissue section was compared, demonstrating that high levels of AR predicts high levels of repair proteins.

Spearman´s rank correlation Before castration	Patient	Mean intensity of nuclear Ku70 (arb. units)		Mean intensity of nuclear P-DNA-PKcs (arb. units)	
		Rho	*p* value	Rho	*p* value
Mean intensity of nuclear AR (arb. units)	A	0.650	<0.001	0.019	NS
	B	0.533	<0.001	0.249	<0.001
	C	0.750	<0.001	0.221	0.003
	D	0.312	<0.001	-0.181	0.013
	E	0.789	<0.001	0.407	<0.001

Spearman´s rank correlation After castration	Patient	Mean intensity of nuclear Ku70 (arb. units)		Mean intensity of nuclear P-DNA-PKcs (arb. units)	
		Rho	*p* value	Rho	*p* value
Mean intensity of nuclear AR (arb. units)	A	0.896	<0.001	0.411	<0.001
	B	0.364	<0.001	0.296	<0.001
	C	0.338	<0.001	0.548	<0.001
	D	0.712	<0.001	0.296	0.001
	E	0.645	<0.001	0.191	0.003

Spearman´s rank correlation After castration and RT	Patient	Mean intensity of nuclear Ku70 (arb. units)		Mean intensity of nuclear P-DNA-PKcs (arb. units)	
		Rho	*p* value	Rho	*p* value
Mean intensity of nuclear AR (arb. units)	A	ND		ND	
	B	0.714	<0.001	0.557	0.001
	C	ND		ND	
	D	ND		ND	
	E	0.659	<0.001	0.621	<0.001

A 2-tailed Spearman´s rank correlation test was used for statistics. Abbreviations: AR; Androgen receptor, RT; Radiation therapy, P-DNA-PKcs; phosphorylated DNA-protein kinase catalytic subunit, arb. units; arbitrary units; ND; not done; NS; non-significant

Supplementary Table 2: Clinical data. Data collected pre- and post-ADT for the three patients.

	Patient 1	Patient 2	Patient 3
Initial status			
Clinical staging	Local advanced non-metastatic PCa	Local advanced PCa with initially bone metastases	Local advanced PCa with bone metastases and progression to retroperitoneal lymph nodes
PSA [ng/mL]	41	10780	65
Palpation	Enlarged, solid, stiff and nodular on right side	Enlarged, solid nodular	Enlarged, solid, nodular on right apex
TNM classification	T3N0M0	T4NxM1	T3N0M1
Skeletal scintigraphy	Negative	Positive: Metastasis with BSI-index 8%	Positive: Metastasis with BSI-index 8%
CT thorax/abdomen	Negative	ND	Retroperitoneal lymph node metastasis
MR	Tumor in prostate with seminal vesicle invasion (2015)	ND	ND
Biopsies	For diagnosis*: 8 needle core biopsies were taken, all showed poorly differentiated adenocarcinoma Gleason score 9 (4+5), Grade Group 5.<br>For research: 4 biopsies were taken and annotated (Suppl. Fig. 3)	For diagnosis*: 4 needle core biopsies were taken, all showed poorly differentiated adenocarcinoma Gleason score 8 (4+4), Grade Group 4.<br>For research: 4 biopsies were taken and annotated (Suppl. Fig. 3)	For diagnosis*: 12 needle core biopsies were taken, 8 of 12 poorly differentiated adenocarcinoma Gleason score 9 (4+5), Grade Group 5.<br>For research: 4 biopsies were taken and annotated (Suppl. Fig. 3)
Diagnosis	C619, malignant tumor in prostate	C619, malignant tumor in prostate	C619, malignant tumor in prostate
Treatment	GnRH-analogue (Eligard)	GnRH-analogue (Eligard)	GnRH-analogue (Eligard)
After 8 weeks of GnRH-analogue			
PSA [ng/mL] nadir	0.17	2.1	17
CRPC (year)	No (2016)	2016	2016
PSA [ng/mL], year	<0.1, 2016<br><0.1, 2017	59, 2016<br>90, 2017	274, 2016<br>1033, 2017

* not analyzed in this paper

Abbreviations: ND; Not determined, CRPC; castration resistant prostate cancer, PSA; Prostate specific antigen, LUTS; Lower Urinary Tract Symptoms, PCA; prostate cancer, CT; computed tomography

Supplementary Table 3: The ratio between the chromogranin A and DNA marked area within the region for a given factor. The ratio is expressed as % chromogranin/DNA area.

	Non-Resistant (N-R)			Resistant (R)					
Factor	F2	F3	F5	F4	F6	F7	F10	F12	F14
Ratio	2.3	3.9	0.1	0.7	1.1	0.0	2.1	0.4	1.9
					0.4	1.2			
					0.0	6.6			
					0.3				
Mean ratio factor									
Mean (SEM)	2.3	3.9	0.1	07.	0.4 (0.20)	2.6 (1.67)	2.1	0.4	1.9
Mean N-R, R (SEM)	2.1 (0.91)			1.4 (0.36)					

Mann-Whitney, resistant factors > non-resistant factors, single sided, $p = 0.35$

Supplementary Table 4: The ratio between the chromogranin A and DNA marked area for the whole biopsy. The ratio is expressed as % chromogranin area/DNA area.

Patient 2, Biopsies	2 pre ADT	3 pre ADT	4 pre ADT	1 post ADT	4 post ADT
Ratio, whole biopsy	2.6	2.9	1.8	3.0	3.5

Mann-whitney, resistant factors (factor values from table 3) > whole biopsies, single sided, $p = 0.022$

Supplementary Table 5: Gene markers up- and downregulated in pre-existent resistant cells (left) in patient 1 and 2 from top 50 genes. q-value < 0.05 and logFC > 0.3 for patient 1 and > 0.5 for patient 2) calculated with two-sided Wilcoxon rank-sum test adjusted for multiple comparisons. Related to Fig. 4.

Gene markers upregulated in pre-existent resistant cells	Gene markers downregulated in pre-existent resistant cells
DHCR24	SPINK1
SNHG25	NCAPD3
TRPM8	ABHD2
IFI6	TMEFF2
H2AFJ	COL5A2
FKBP2	FABP5P3
EDF1	AGR2
PPDPF	HSPA8
CTSD	CTD-2290C23.1
C4B	COX6C
TAGLN	CD46
MYL9	CTD-2287O16.1
APOE	AC016712.2
NBL1	AC010468.1
TIMP1	PABPC1
AEBP1	PABPC3
CD74	SFTPA2
HLA-DRA	GDF5
IGFBP7	MIPEP
MGP	SEMA3C
A2M	MAOA
	EEF1A1P5
	EEF1A1P13
	C1QTNF3
	HPGD
	ACAD8
	CYP2U1
	FAM3B
	SCD

Supplementary Table 6: Subset of genes upregulated in non-responding spots in patient 2.

Gene	Cancer correlation
DHCR24, TRPM8, IF16	*DHCR24* is involved in cholesterol biosynthesis and regulated by the AR, and participates in the conversion of adrenal androgen into its more potent forms; testosterone and dihydrotestosterone. Blocking these pathways might help to reduce treatment resistance. The androgen regulated *TRPM8*, might play an important role in proliferation and apoptosis, and has further been associated with various cancers.<br>IFI6 is an antiapoptotic protein that promotes metastasis in breast cancer.
CD74	The membrane receptor CD74 is upregulated in the non-responsive areas both in patient 1 and 2, and promotes an increased proliferation, migration and metastatic potential in several cancers. It has a role in NF-κB activation, leading to an immunosuppressive environment. It also plays a role in chemokine production of the potent CCL2, enabling attraction of myeloid-derived suppressor cells and TAMs to the tumor. CD74 interacts with the proinflammatory cytokine macrophage migration inhibitory factor (MIF*)* and has shown to induce EMT.
TIMP1	A secreted glycoprotein that has been linked to promotion of tumor progression by inhibition of apoptosis and stimulation of prostate cancer cell growth. Elevated levels of TIMP1 levels in plasma predict worse survival outcome in metastatic CRPC patients.
IGFBP7, MGP	The tumor stroma marker IGFBP7 regulates the insulin pathway and has been shown to be elevated in invasive prostate neoplasms. *MGP* expression is known to be upregulated in CAFs in PCa.<br><br>Matrix Gla-protein (MGP) belongs to the family of matricellular proteins (MCP) and induces changes in the extra cellular matrix (ECM) crosslinking and is linked to migration.
A2M	Enzymatic activity of PSA-A2M is present in the serum of men with advanced prostate cancer, which in turn affect a range of growth factors such as IL-6, TGF-beta, PDGF, and FGF, leading to tumor microenvironmental changes.

Supplementary Table 7: Stromal gene markers upregulated and downregulated around pre-existent resistant cells in patient 2 and 3. Adjusted *p*-value < 0.05, average logFC > 0.5, calculated with two-sided Wilcoxon rank-sum test adjusted for multiple comparisons

**Gene markers elevated in stroma compartment around pre-existent resistant cells**	**Gene markers downregulated in stroma compartment around pre-existent resistant cells**
SPARC	MSMB
COL1A1	ACTG2
COL1A2	DES
COL3A1	MYH11
SPINK1	MYL9
PTGDS	
CCDC3	
POSTN	
AEBP1	
THBS2	
LRRC32	
FTLP3	
ATXN2L	
SPON2	
FAM3B	
BGN	
TIMP1	
SULF1	
COM	
CXCL14	
SFRP4	

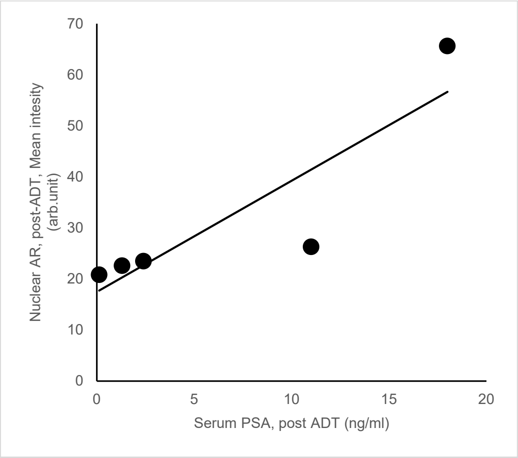

Supplementary Figure 1: Correlation between the main intensity of nuclear AR and serum PSA levels in 5 patients post-ADT. Spearman's rho correlation test ϱ = 0.99, $p < 0.001$. Although with a limited set of patients, a significant correlation between AR in nucleus and PSA-levels could indicate a causative relationship, thus demonstrating that these cells contribute to treatment resistance. This was expected since AR (in the nucleus) functions as a transcription factor for PSA expression. Source data is provided as Source Data file. Abbreviations: ADT; androgen deprivation therapy, AR; androgen receptor, PSA; prostate specific antigen

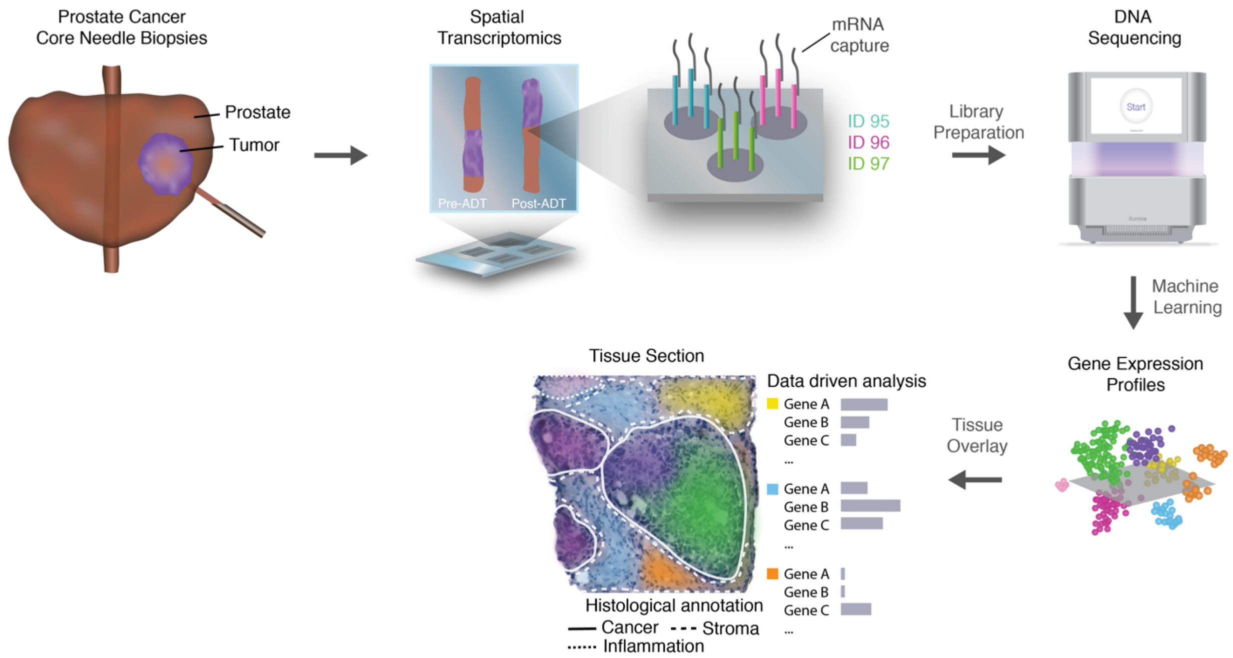

Supplementary Figure 2: Spatial Transcriptomics workflow and UMAP visualization of factor activities. Similar color depicts similar gene expression profile.

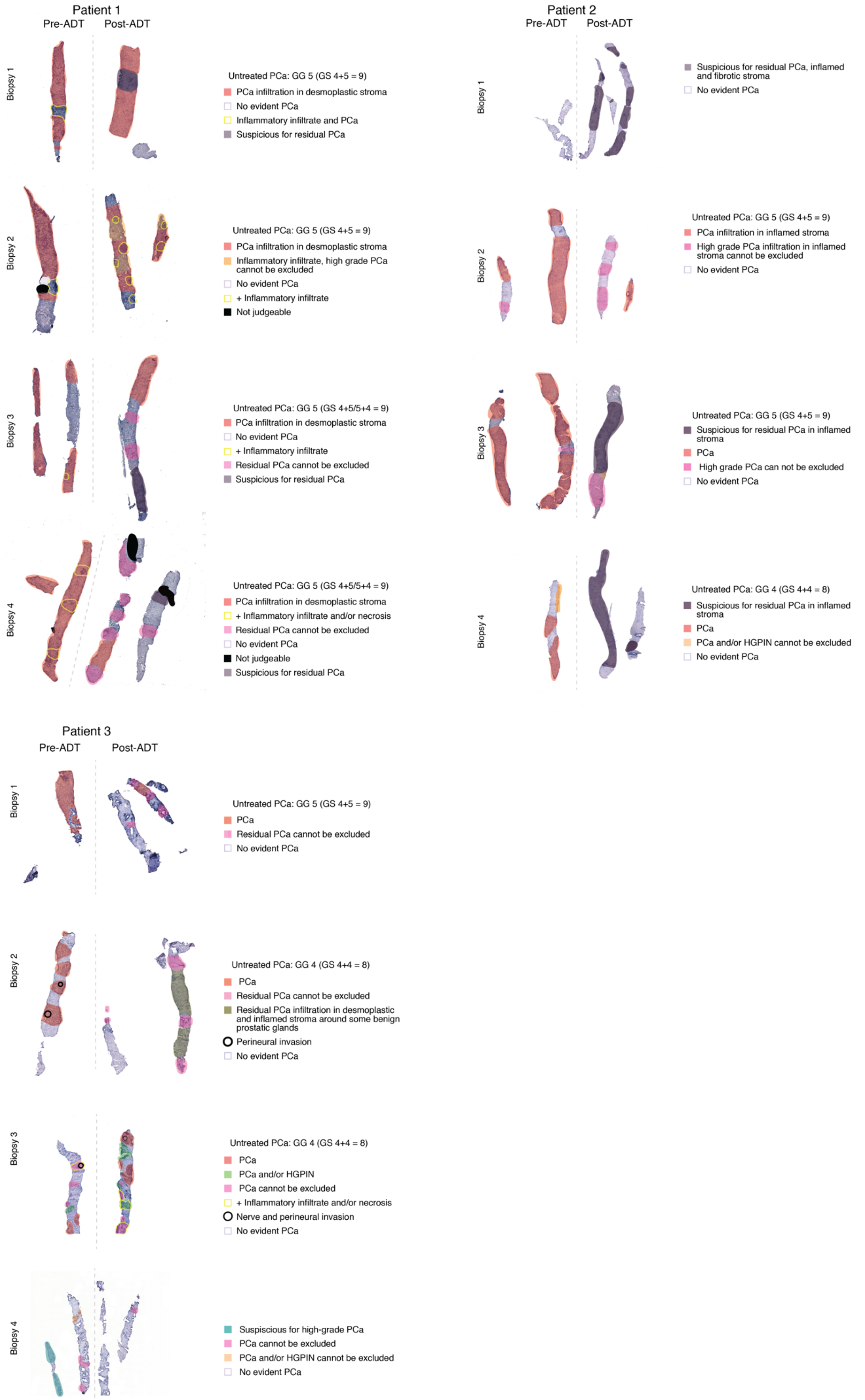

Supplementary Figure 3: Histologic annotations for patient 1, 2 and 3. The sections were annotated for the presence of PCa. Information about areas of suspected PCa, residual PCa post-ADT, HGPIN and immune response/inflammation was also given.
Abbreviations: PCa; Prostate cancer, GG; Grade Group, GS; Gleason Score, HGPIN; high grade prostatic intraepithelial neoplasia

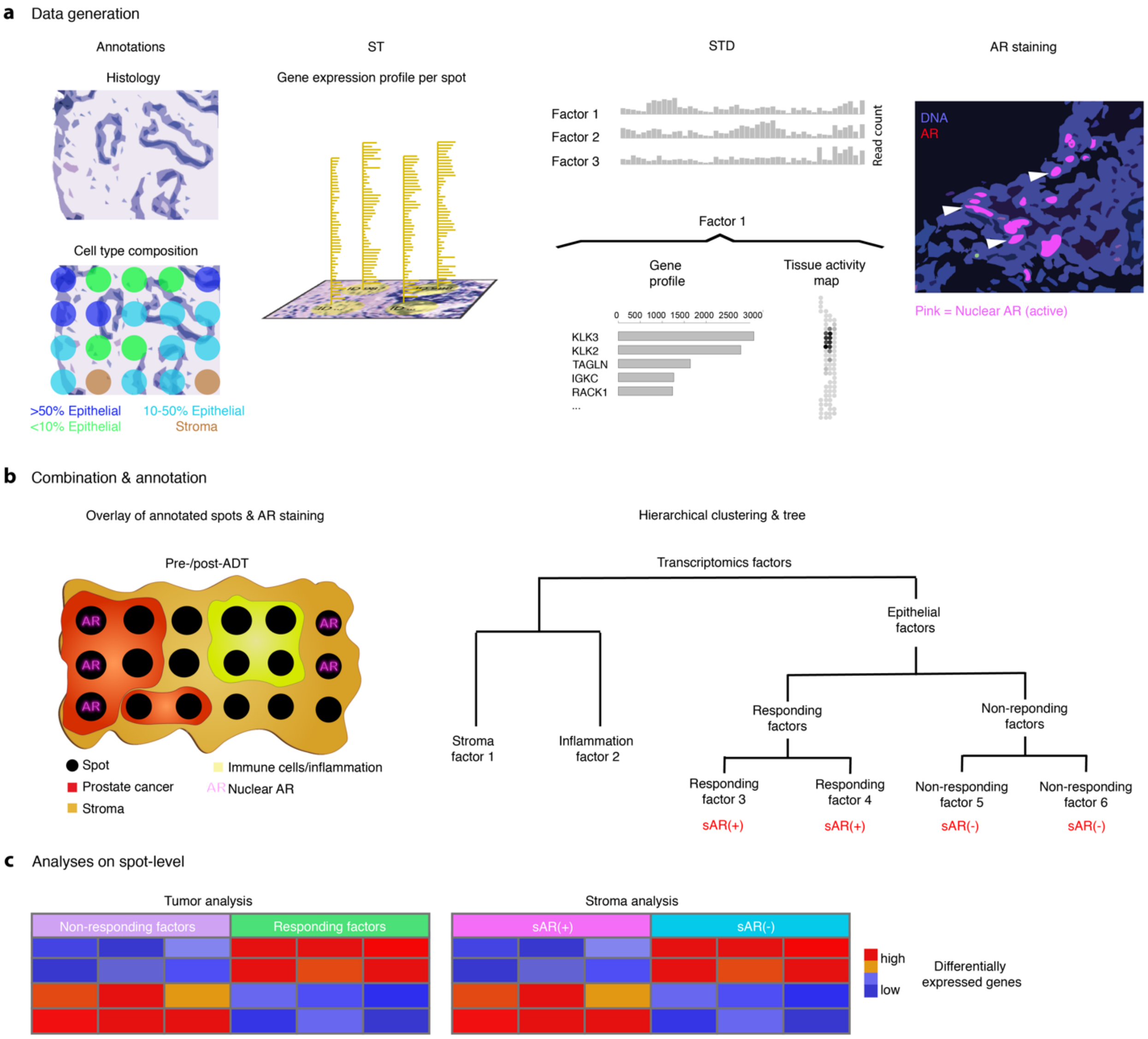

Supplementary Figure. 4: Overview of collected data, their combination and annotation, and the performed analyses. a, Data generation: annotations, ST, STD (performed individually on the patients) with given gene expression profiles (factors) and gene activity maps, and AR staining to determine spatial localization of nuclear AR. **b,** Annotation of the combined data per patient. **c,** Differential gene expression analysis was performed on spot level to compare responding to non-responding factors and sAR(+) areas to sAR(-) areas, and presented as heatmaps.
Abbreviations: AR; androgen receptor, ADT; androgen deprivation therapy, sAR(+); stromal nuclear AR positive, sAR(-); stromal nuclear AR negative, ST; Spatial Transcriptomics, STD; Spatial Transcriptome Decomposition

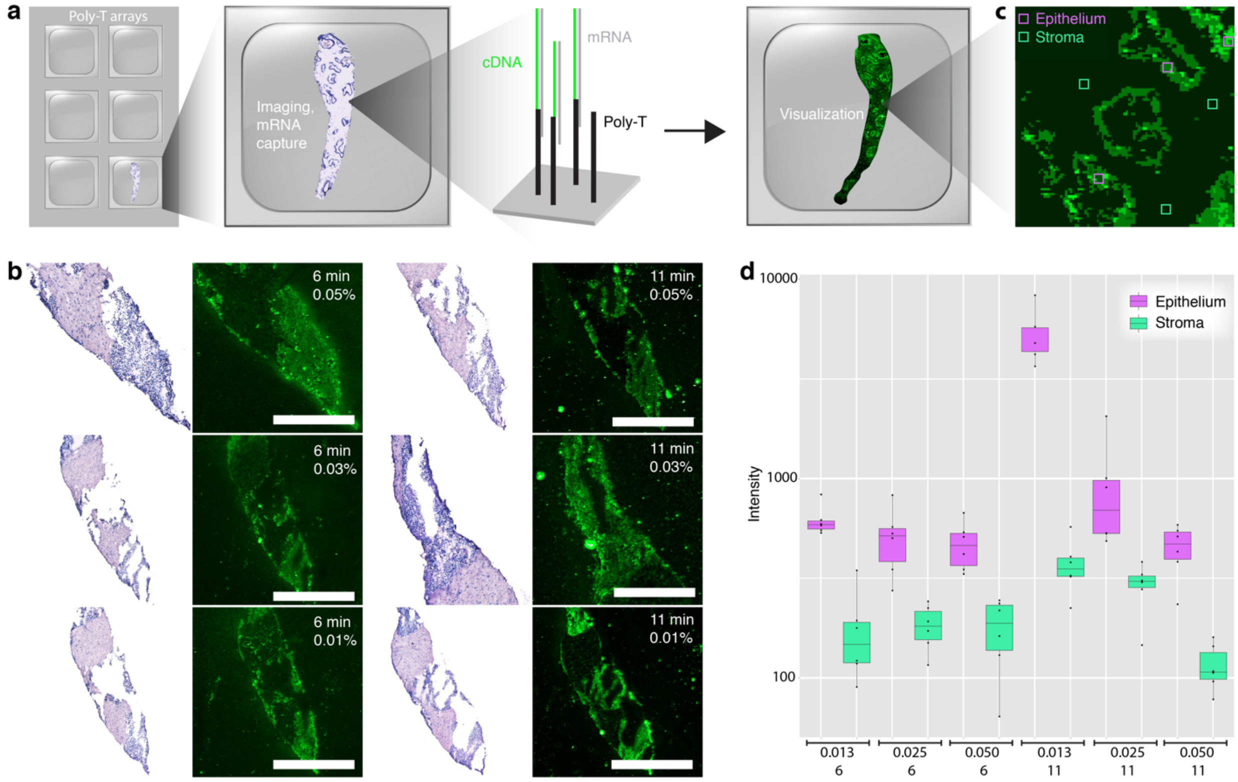

Supplementary Figure 5: Spatial Transcriptomics quality control assay. a, Schematic of the Spatial Transcriptomics quality control assay. Tissue sections are placed onto glass slides with microarrays uniformly coated with poly-T capturing oligonucleotides. Permeabilization allows mRNAs to be captured onto an array followed by reverse transcription with incorporation of Cy3-labeled nucleotides. Removal of the tissue section allows for measuring the cDNA footprint. **b,** Different permeabilization times and pepsin concentrations were tested on two consecutive tissue sections (only singlets shown) to minimize risk for including non-biological-relevant Cy3-aggregations. All pepsin concentrations had 0.1 M HCl. **c,** cDNA footprint signal was estimated by taking intensity subtracted with background on three epithelial and three stroma cell areas per duplicate using GenePix Pro (87/91 in Brightness/Contrast). This gave six values per treatment when averaging the duplicates. Background on each well was determined by taking the average of three intensity values on the background area. Scale bars 1 mm. **d,** Boxplot showing net intensities (n=144 tissue areas). The boxes show the mean and quartiles of the dataset while the whiskers extend to 1.5 of the interquartile range, points beyond the whiskers are considered to be outliers. The highest values for both epithelial and stromal areas is seen at 11 min with 0.013% and these parameters were chosen for further experiments. Source data is provided as Source Data file.

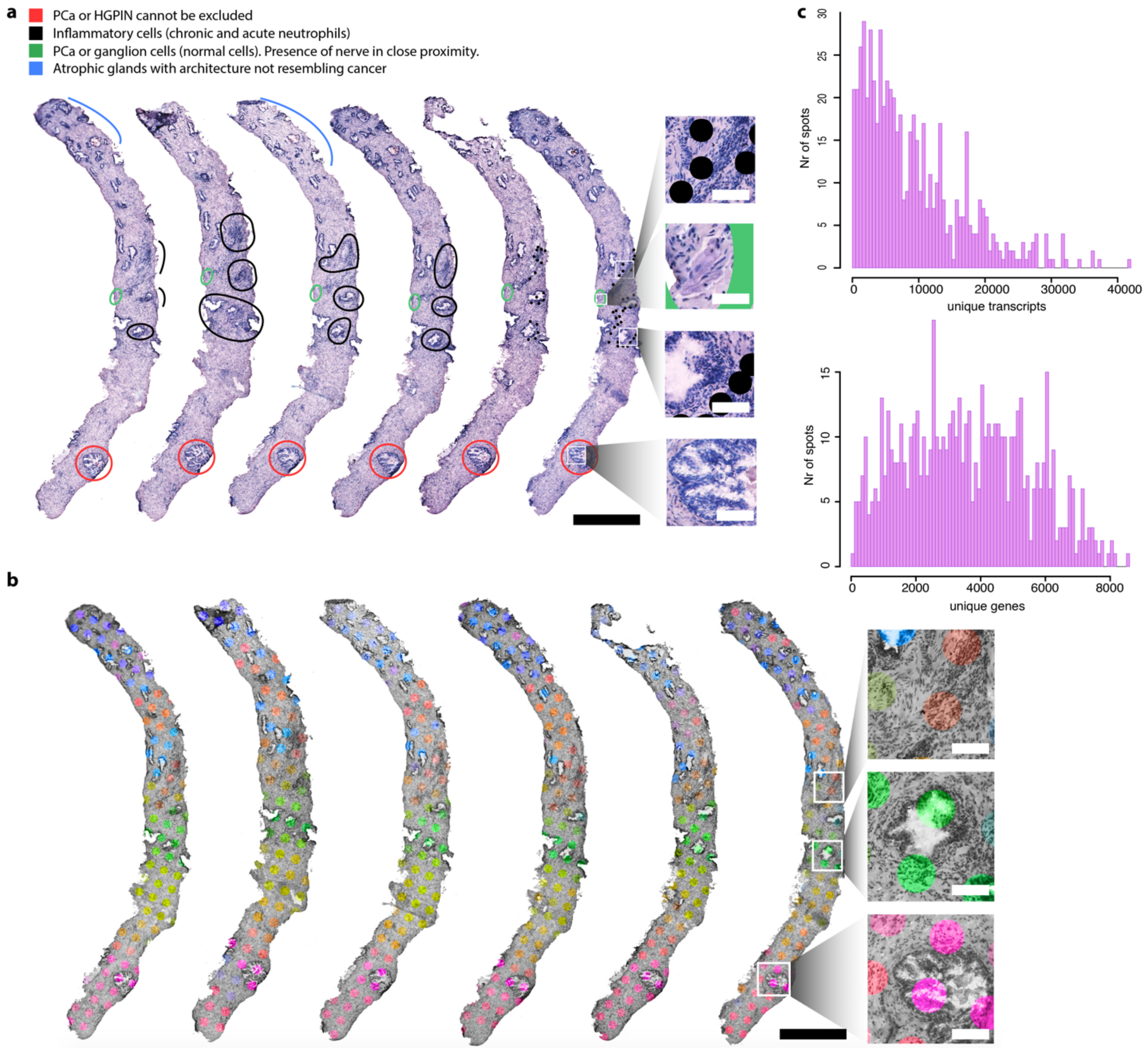

Supplementary Figure 6: ST tissue optimization for core needle biopsies and sequencing statistics for the three patients. a, Histological annotation of six consecutive tissue sections. The blue areas contain prostate cancer mimicking areas called atrophic glands. Black circles and dots contain inflammatory cells, both chronic and acute neutrophils. Green areas are either prostate cancer or Ganglion cells (found only in periprostatic soft tissue). The red area had not sufficient resolution in order to conclude whether it is prostate cancer or HGPIN. Scale bar in whole figure, 1 mm, scale bars in close up, 100 μm. **b.** tSNE overlay on HE images shows high specificity and robustness for optimized ST-protocol. Similar colors indicate similar gene expression. Scale bar in whole figure, 1 mm, scale bars in close up, 100 μm. **c,** Statistics for sequencing data for the three patients showing unique transcripts and genes per spot. Source data are provided as a Source Data file. Abbreviations: ST; Spatial Transcriptomics, DEGs; differentially expressed genes, HGPIN; high grade prostatic intraepithelial neoplasia

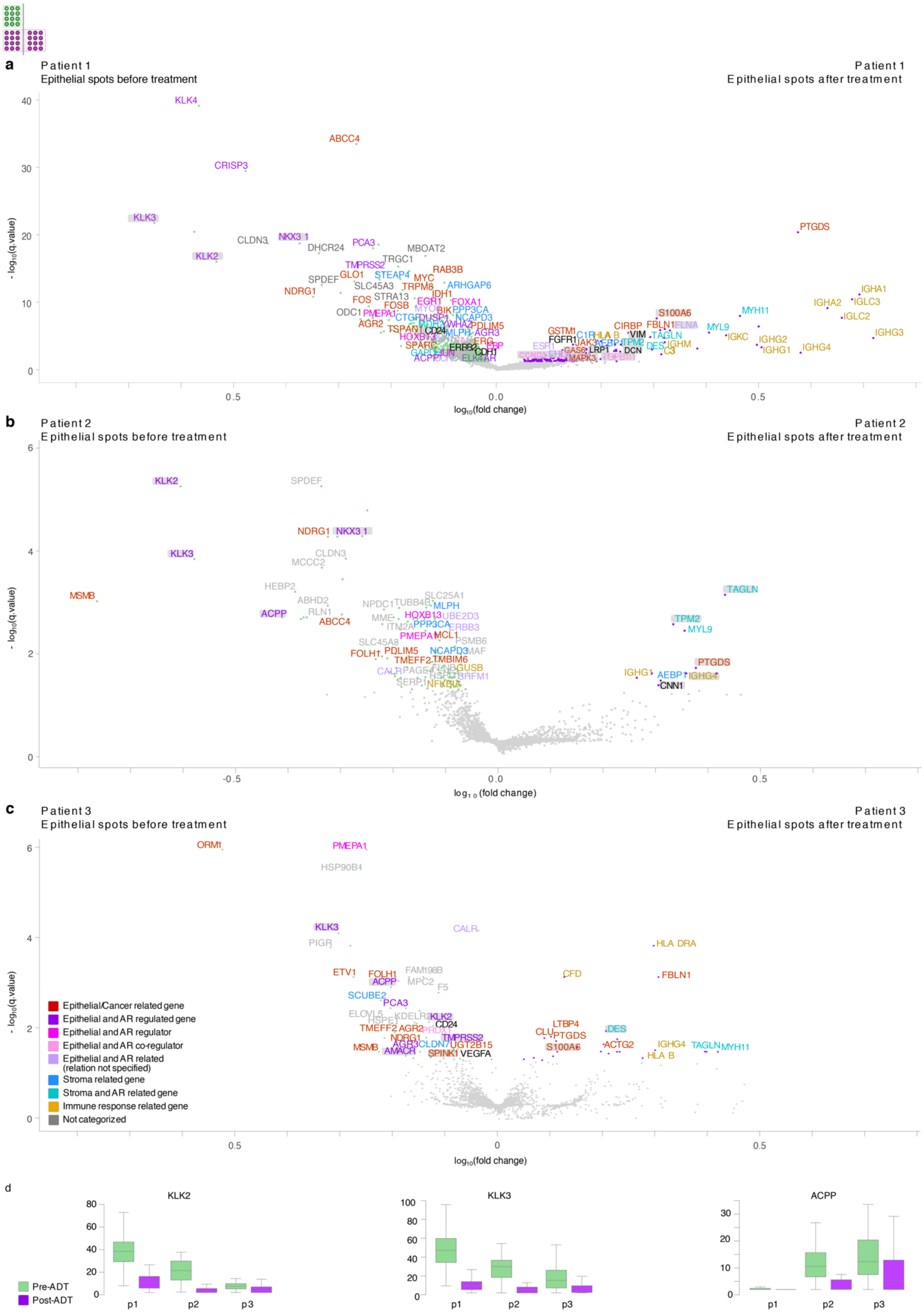

Supplementary Fig. 7a-d: DEGs (Welch's t-test) and pathway annotation for bulk epithelial spots of patients 1, 2 and 3 pre- and post-ADT. **a,** For patient 1, 124 epithelial spots pre- and 51 epithelial spots post-ADT were compared. Pathway annotation was performed on DEGs ($q < 0.01$). **b,** For patient 2, 36 epithelial spots pre- and 21 epithelial spots post-ADT were compared. Pathway annotation was performed on DEGs ($q < 0.05$). **c,** For patient 3, 54 epithelial spots pre- and 88 epithelial spots post-ADT were compared. Pathway annotation was performed on DEGs ($q < 0.05$). **a-c,** Genes shaded gray are relevant to PCa or genes of interest. Source data are provided as Source Data files. **d,** Boxplots for the AR regulated genes *KLK2*, *KLK3* and *ACPP* using the same spots as in a-c. The boxes show the median and the quartiles of the dataset while the whiskers extend to 1.5 of the interquartile range. Abbreviations: DEGs; Differentially expressed genes

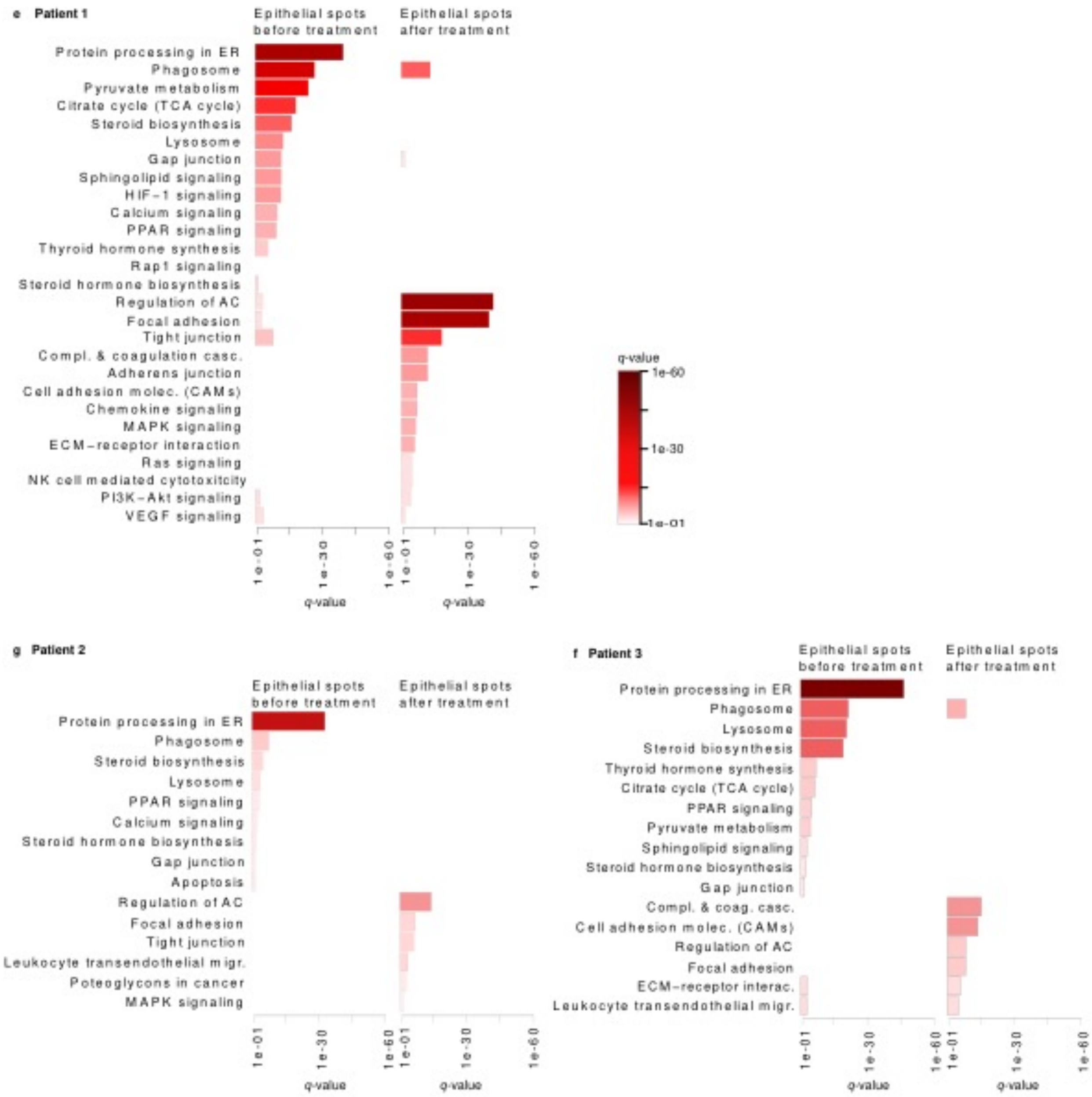

Supplementary Fig. 7e-g: Pathway annotation for DEGs shown in Supplementary Figure 7a-c for bulk epithelial spots of the patients 1, 2 and 3 pre- and post-ADT. Pathway annotation was performed on DEGs **e,** for patient 1 ($q < 0.01$) **f,** for patient 2 ($q < 0.05$) **g,** for patient 3 ($q < 0.05$).
Abbreviations: DEGs; differentially expressed genes, ER; endoplasmic reticulum, AC; actin cytoskeleton, MAPK; mitogen-activated protein kinase, PPAR; peroxisome proliferator-activated receptors, ECM; extracellular matrix, TCA; tricarboxylic acid, VEGF; vascular endothelial growth factor, NK; natural killer, PI3K; phosphoinositide 3-kinases, HIF; hypoxia-inducible factors

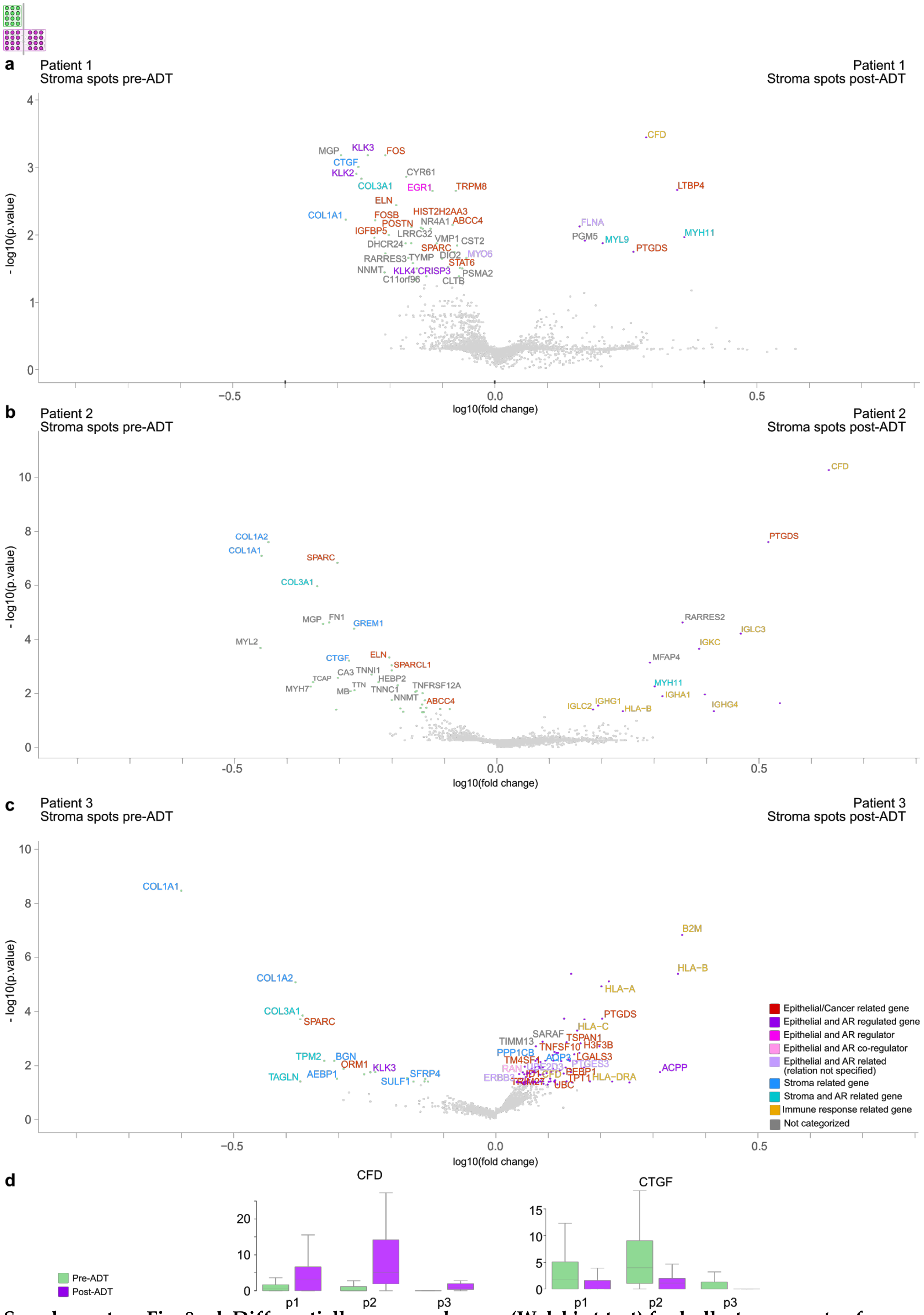

Supplementary Fig. 8a-d: Differentially expressed genes (Welch's *t*-test) for bulk stroma spots of patients 1, 2 and 3 pre- and post-ADT. a, Patient 1, 75 stroma spots pre- and 62 stroma spots post-ADT were compared. **b,** Patient 2, 75 stroma spots pre- and 70 stroma spots post-ADT were compared. **c,** Patient 3, 39 stroma spots pre- and 38 stroma spots post-ADT were compared. **d,** *CFD* and *CTFG* were significantly differentially expressed in the three patients; the same spots as in a-c were used. The boxes show the median and the quartiles of the dataset while the whiskers extend to 1.5 of the interquartile range.

Abbreviations: DEGs; differentially expressed genes

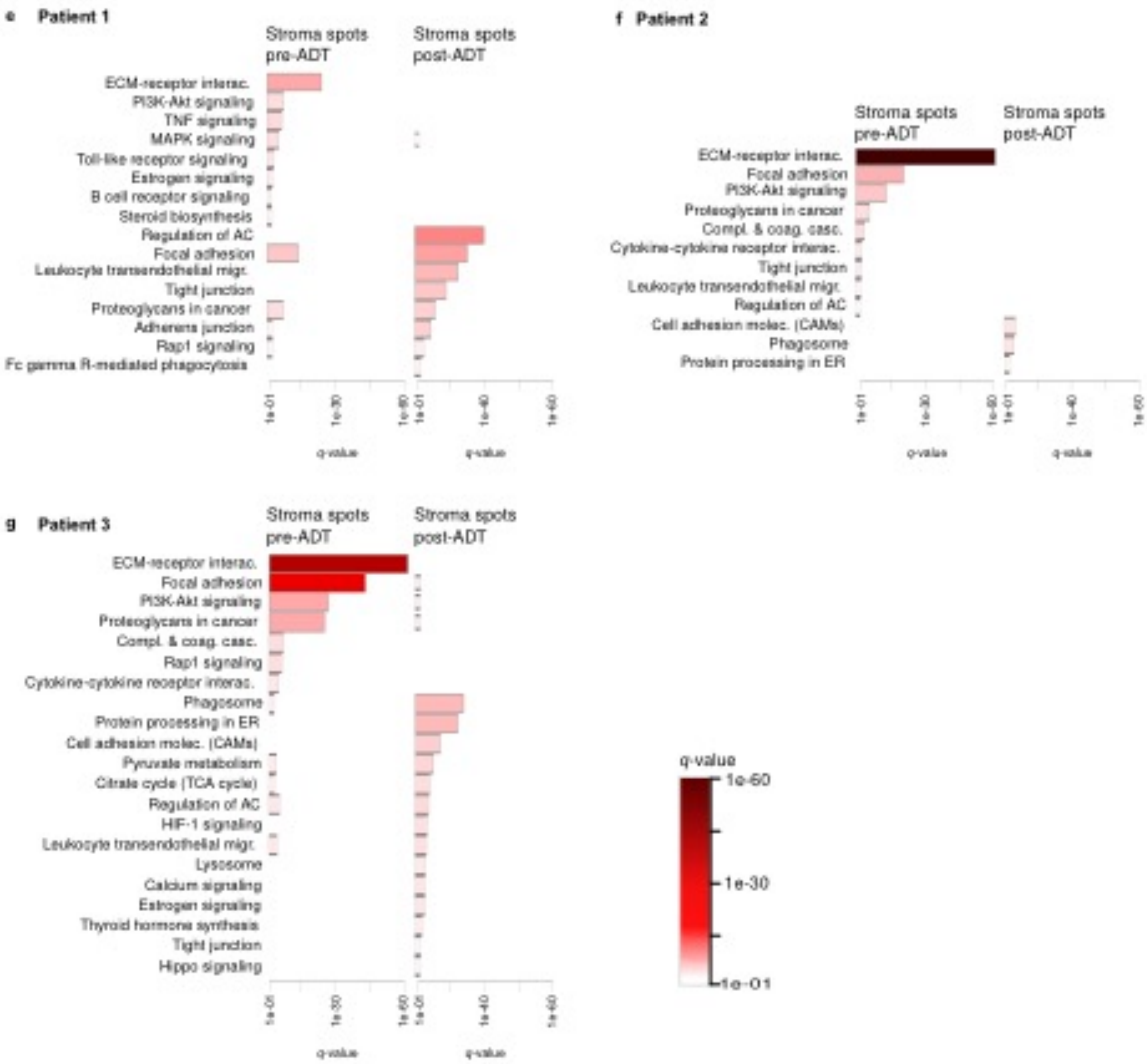

Supplementary Fig. 8e-g: Pathway annotation for DEGs shown in Supplementary Figure 8a-c for bulk stromal spots of patients 1, 2 and 3 pre- and post-ADT. ECM-receptor interaction is the highest activated pathway pre-ADT in the three patients. Post-ADT are the pathways Cell adhesion molecules and Phagosome dominant in patients 2 and 3. Pathway annotation was performed **e,** for patient 1 on DEGs ($q < 0.05$) **f,** for patient 2 on DEGs ($q < 0.05$) **g,** for patient 3 on DEGs ($q < 0.05$). Abbreviations: DEGs; Differentially expressed genes, ER; endoplasmic reticulum, AC; actin cytoskeleton, MAPK; mitogen-activated protein kinase, ECM; extracellular matrix, TCA; tricarboxylic acid, PI3K; Phosphoinositide 3-kinases, HIF; Hypoxia-inducible factors

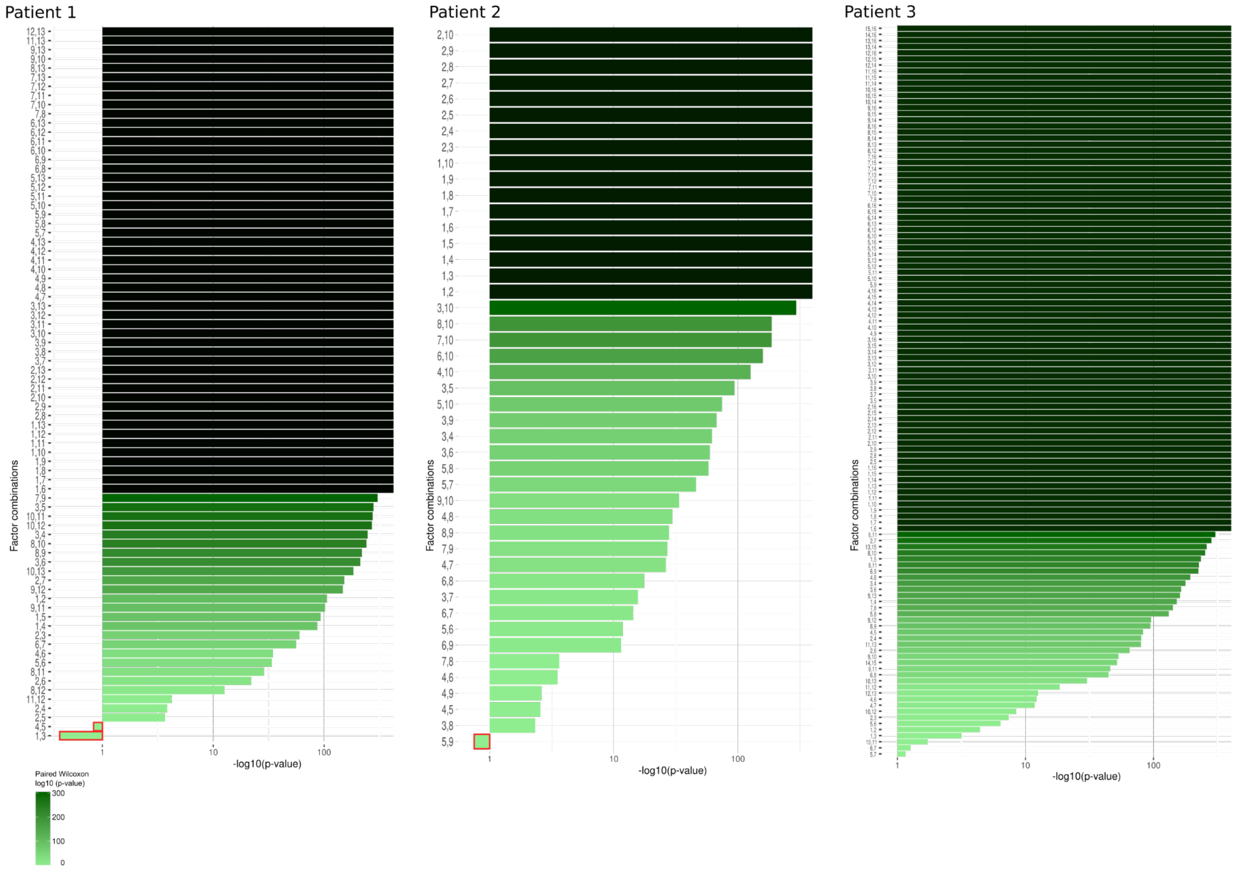

Supplementary Figure 9: Paired Wilcoxon for each possible factor combination of a patient. *P*-values equal to 0 are marked in black. Except for two factor combinations in patient 1, and 1 factor combination in patient 2, the factors of a patient are independent. Source data is provided as Source Data file.

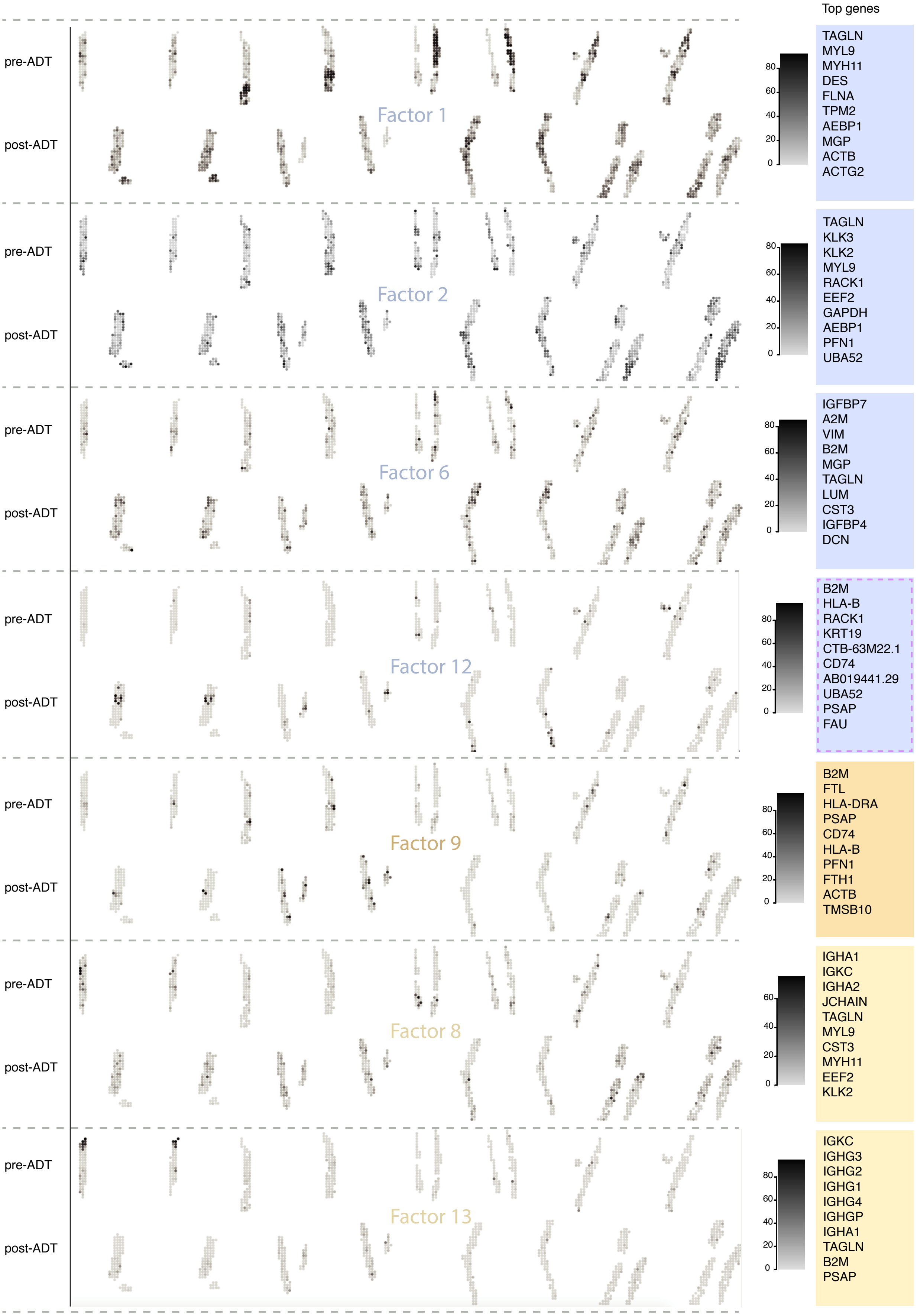
Top genes
pre-ADT
post-ADT
Factor 1
TAGLN MYL9 MYH11 DES FLNA TPM2 AEBP1 MGP ACTB ACTG2
Factor 2
TAGLN KLK3 KLK2 MYL9 RACK1 EEF2 GAPDH AEBP1 PFN1 UBA52
Factor 6
IGFBP7 A2M VIM B2M MGP TAGLN LUM CST3 IGFBP4 DCN
Factor 12
B2M HLA-B RACK1 KRT19 CTB-63M22.1 CD74 AB019441.29 UBA52 PSAP FAU
Factor 9
B2M FTL HLA-DRA PSAP CD74 HLA-B PFN1 FTH1 ACTB TMSB10
Factor 8
IGHA1 IGKC IGHA2 JCHAIN TAGLN MYL9 CST3 MYH11 EEF2 KLK2
Factor 13
IGKC IGHG3 IGHG2 IGHG1 IGHG4 IGHGP IGHA1 TAGLN B2M PSAP

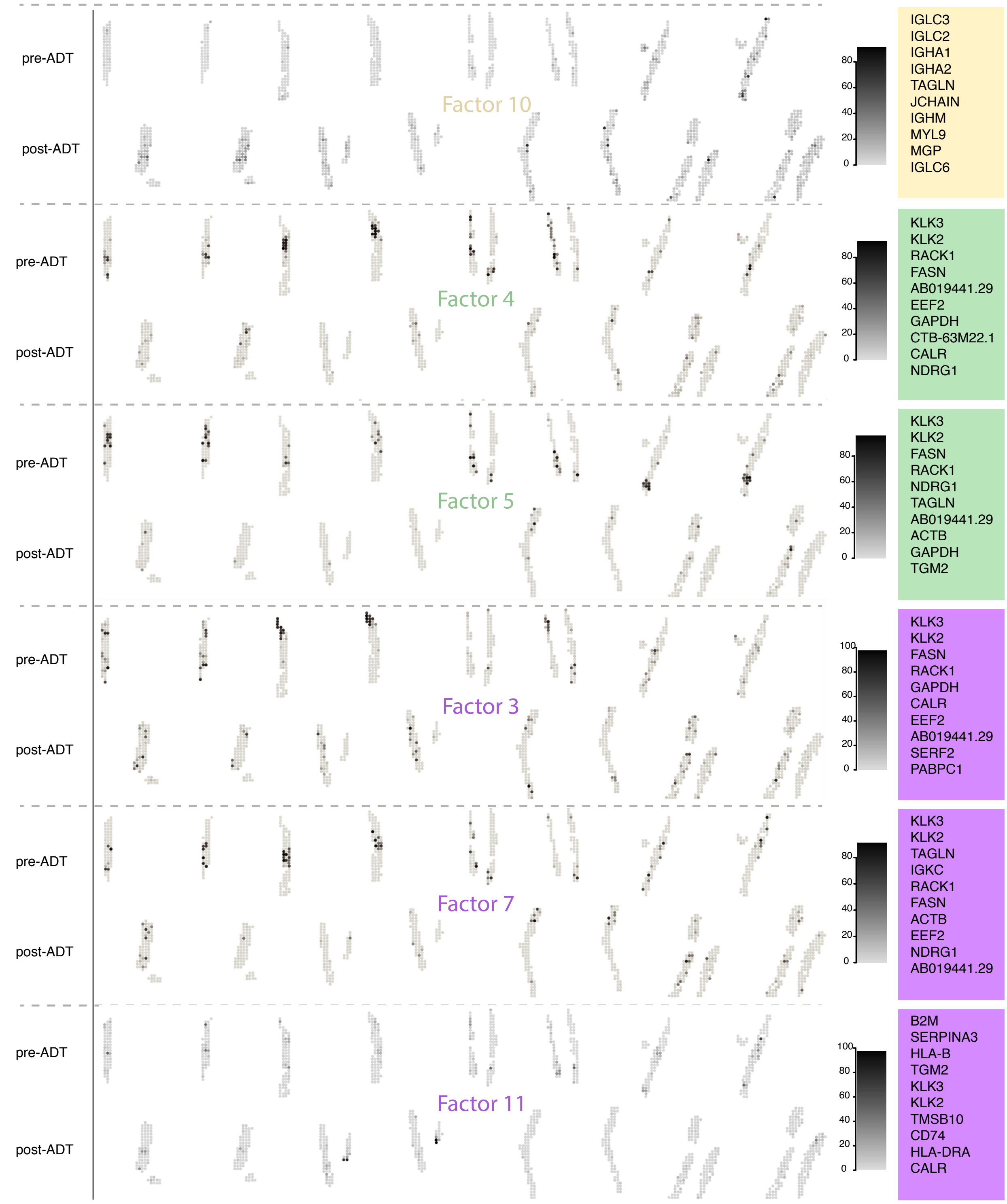

Supplementary Figure 10: Activity maps and top 10 driving genes of transcriptomics factors of STD for patient 1. Color coding depicts stromal factors (blue), mix of stroma and inflammation/immune response (orange), inflammation/immune response (yellow), responding factors (green), and non-responding factors (purple). Factor 12 is a mixed stromal and epithelial factor caused by infiltration of non-responding tumor cells. Color bar shows expected percentage of reads. Abbreviations: ADT; androgen deprivation therapy, STD; Spatial Transcriptome Decomposition

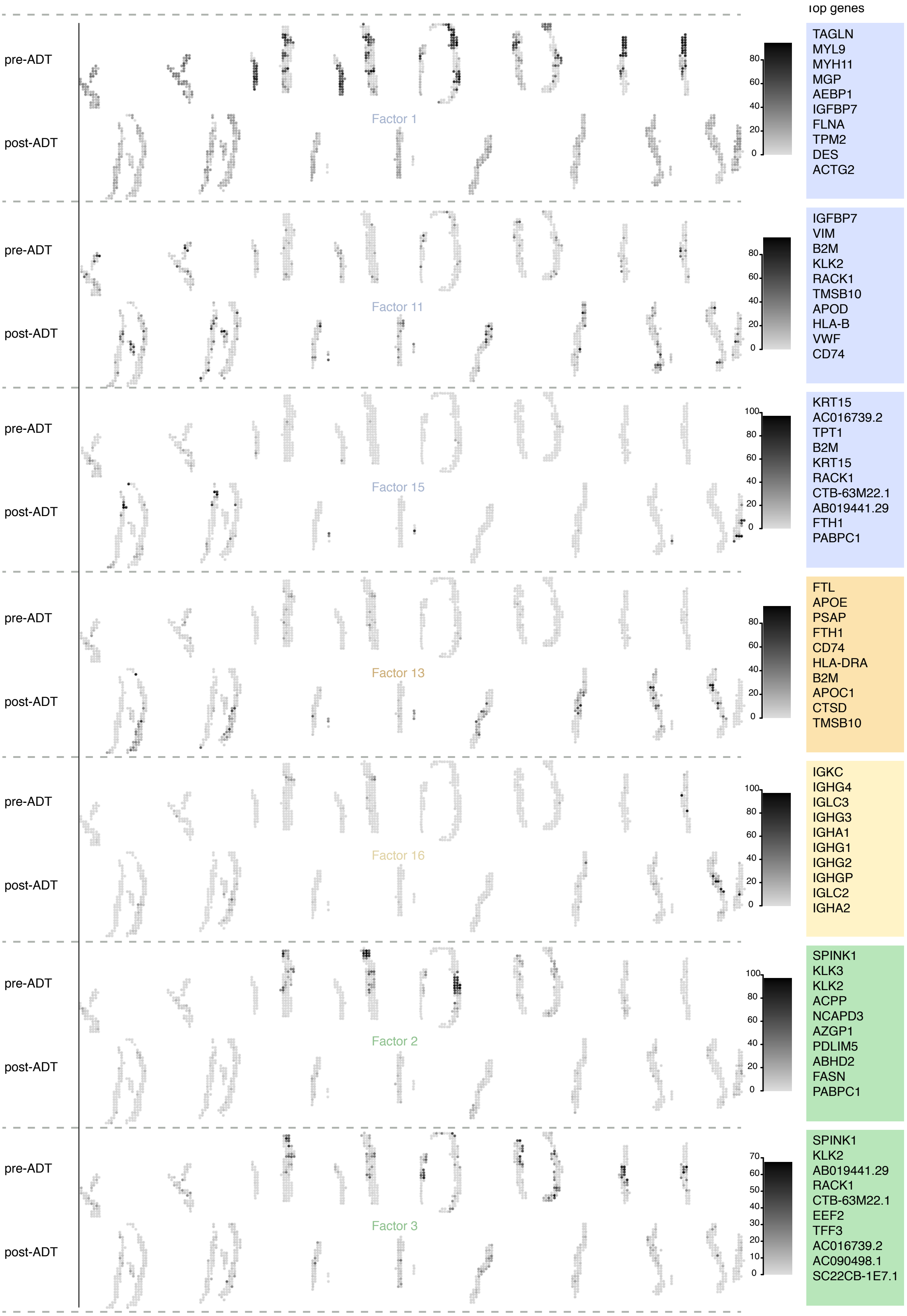
Top genes
pre-ADT
post-ADT
Factor 1
TAGLN
MYL9
MYH11
MGP
AEBP1
IGFBP7
FLNA
TPM2
DES
ACTG2
Factor 11
IGFBP7
VIM
B2M
KLK2
RACK1
TMSB10
APOD
HLA-B
VWF
CD74
Factor 15
KRT15
AC016739.2
TPT1
B2M
KRT15
RACK1
CTB-63M22.1
AB019441.29
FTH1
PABPC1
Factor 13
FTL
APOE
PSAP
FTH1
CD74
HLA-DRA
B2M
APOC1
CTSD
TMSB10
Factor 16
IGKC
IGHG4
IGLC3
IGHG3
IGHA1
IGHG1
IGHG2
IGHGP
IGLC2
IGHA2
Factor 2
SPINK1
KLK3
KLK2
ACPP
NCAPD3
AZGP1
PDLIM5
ABHD2
FASN
PABPC1
Factor 3
SPINK1
KLK2
AB019441.29
RACK1
CTB-63M22.1
EEF2
TFF3
AC016739.2
AC090498.1
SC22CB-1E7.1

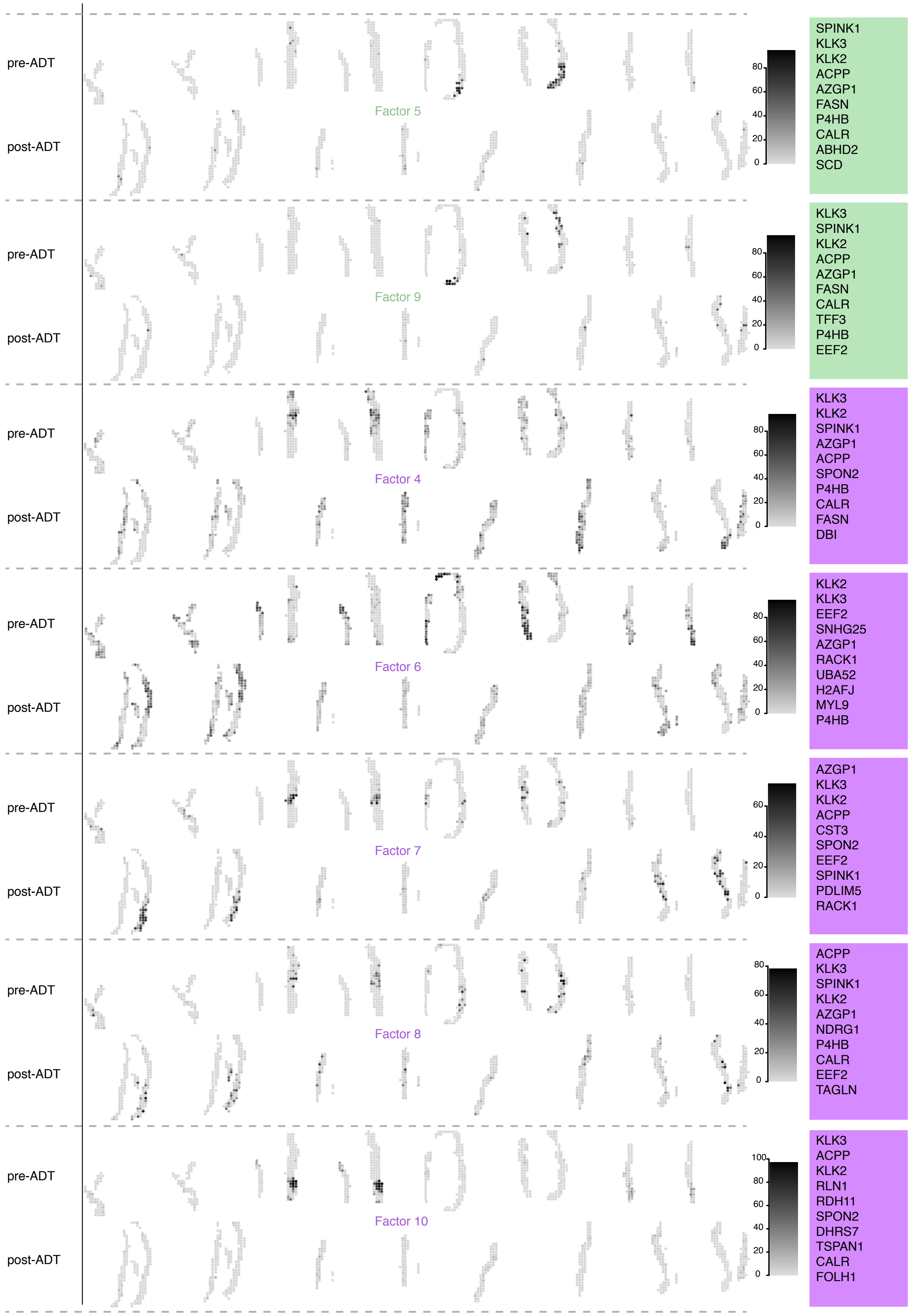

pre-ADT
post-ADT
Factor 5
SPINK1 KLK3 KLK2 ACPP AZGP1 FASN P4HB CALR ABHD2 SCD
Factor 9
KLK3 SPINK1 KLK2 ACPP AZGP1 FASN CALR TFF3 P4HB EEF2
Factor 4
KLK3 KLK2 SPINK1 AZGP1 ACPP SPON2 P4HB CALR FASN DBI
Factor 6
KLK2 KLK3 EEF2 SNHG25 AZGP1 RACK1 UBA52 H2AFJ MYL9 P4HB
Factor 7
AZGP1 KLK3 KLK2 ACPP CST3 SPON2 EEF2 SPINK1 PDLIM5 RACK1
Factor 8
ACPP KLK3 SPINK1 KLK2 AZGP1 NDRG1 P4HB CALR EEF2 TAGLN
Factor 10
KLK3 ACPP KLK2 RLN1 RDH11 SPON2 DHRS7 TSPAN1 CALR FOLH1

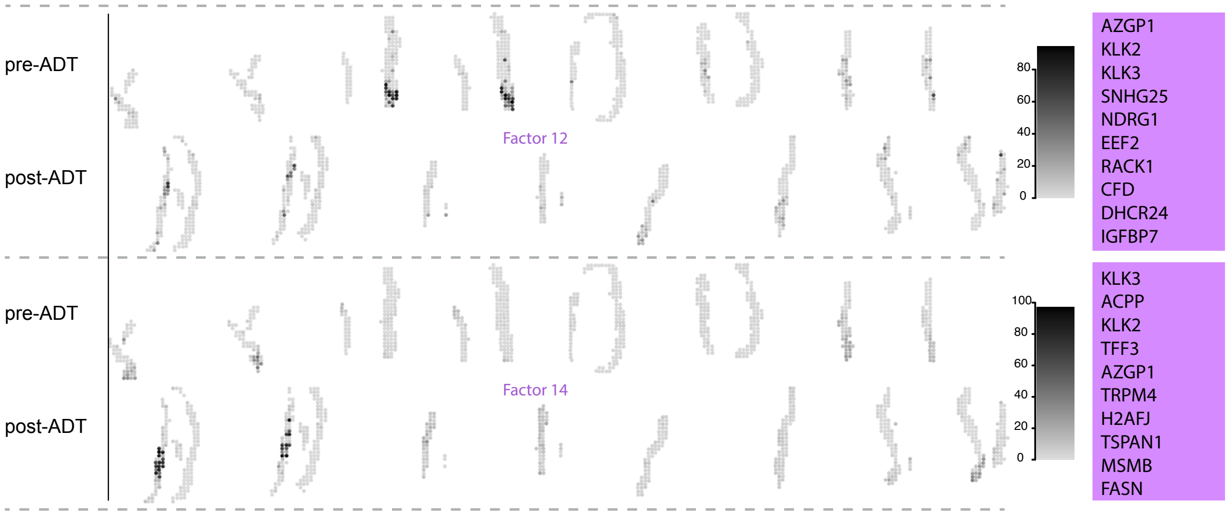

Supplementary Figure 11: Activity maps of transcriptomics factors of STD for patient 2. Color coding depicts stromal factors (blue), mix of stroma and inflammation/immune response (orange), inflammation/immune response (yellow), responding factors (green), and non-responding factors (purple). Factor 3 and 11 is annotated to PCa, inflammation and/or possible HGPIN. Color bar shows expected percentage of reads. Abbreviations: ADT; androgen deprivation therapy, STD; Spatial Transcriptome Decomposition

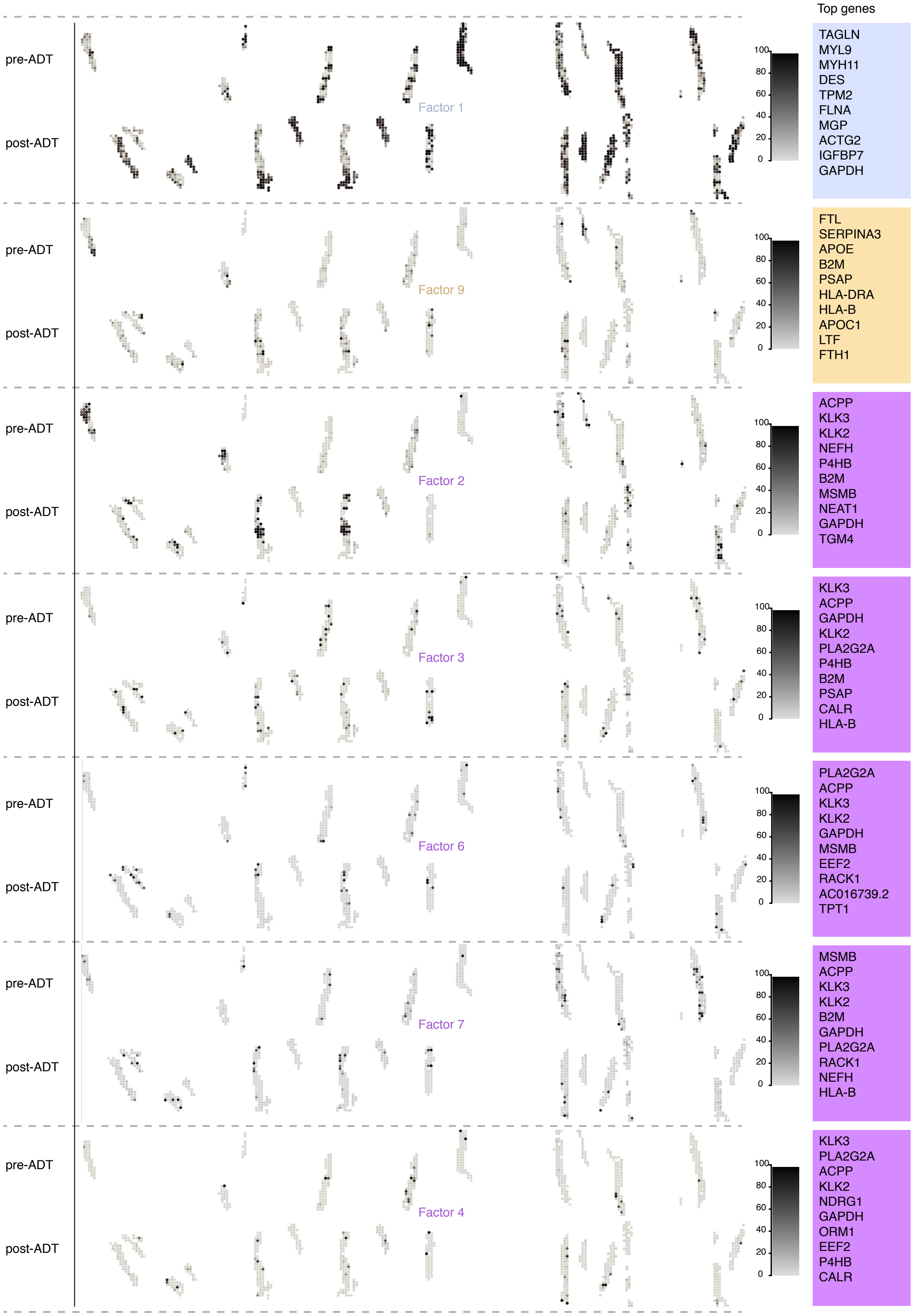
Top genes
pre-ADT
post-ADT
Factor 1
TAGLN
MYL9
MYH11
DES
TPM2
FLNA
MGP
ACTG2
IGFBP7
GAPDH
Factor 9
FTL
SERPINA3
APOE
B2M
PSAP
HLA-DRA
HLA-B
APOC1
LTF
FTH1
Factor 2
ACPP
KLK3
KLK2
NEFH
P4HB
B2M
MSMB
NEAT1
GAPDH
TGM4
Factor 3
KLK3
ACPP
GAPDH
KLK2
PLA2G2A
P4HB
B2M
PSAP
CALR
HLA-B
Factor 6
PLA2G2A
ACPP
KLK3
KLK2
GAPDH
MSMB
EEF2
RACK1
AC016739.2
TPT1
Factor 7
MSMB
ACPP
KLK3
KLK2
B2M
GAPDH
PLA2G2A
RACK1
NEFH
HLA-B
Factor 4
KLK3
PLA2G2A
ACPP
KLK2
NDRG1
GAPDH
ORM1
EEF2
P4HB
CALR
100
80
60
40
20
0

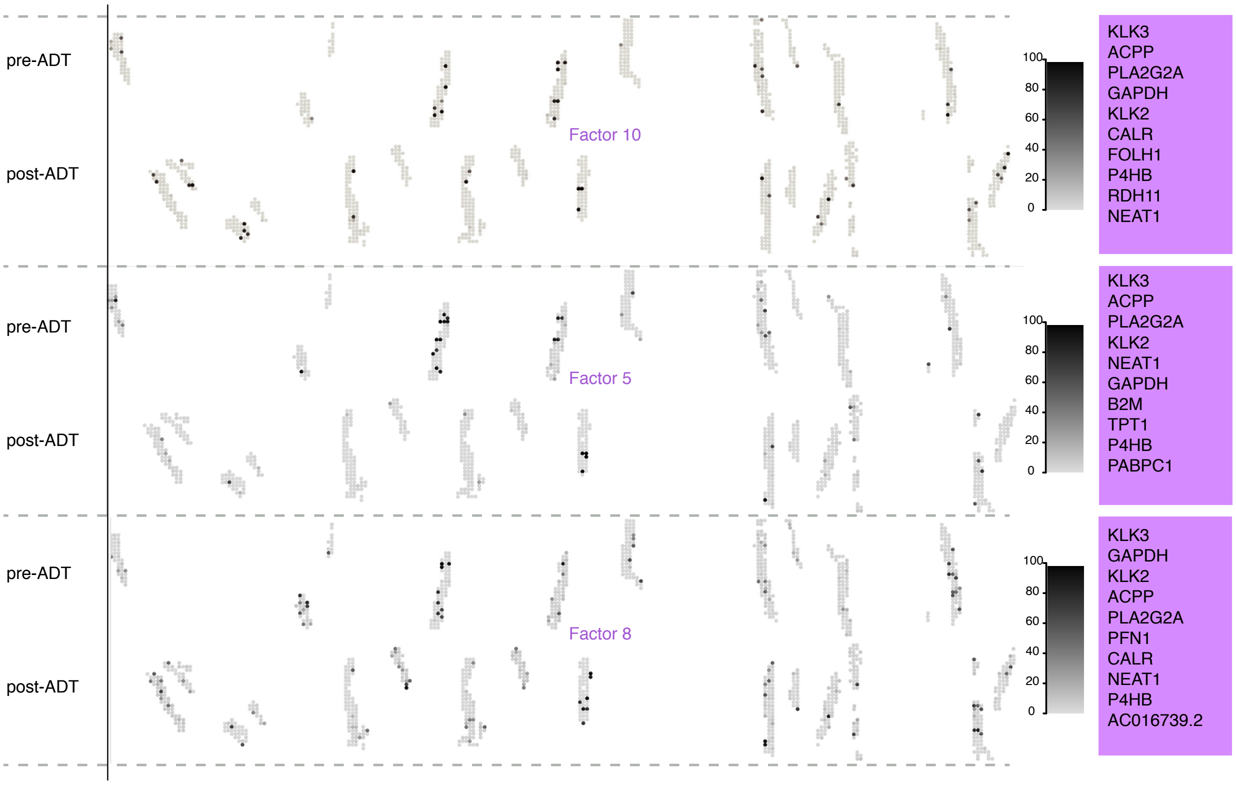

Supplementary Figure 12: Activity maps of transcriptomics factors of STD for patient 3. Color coding depicts stromal factors (blue), mix of stroma and inflammation/immune response (orange), and non-responding factors (purple). Color bar shows expected percentage of reads. Abbreviations: ADT; androgen deprivation therapy, STD; Spatial Transcriptome Decomposition

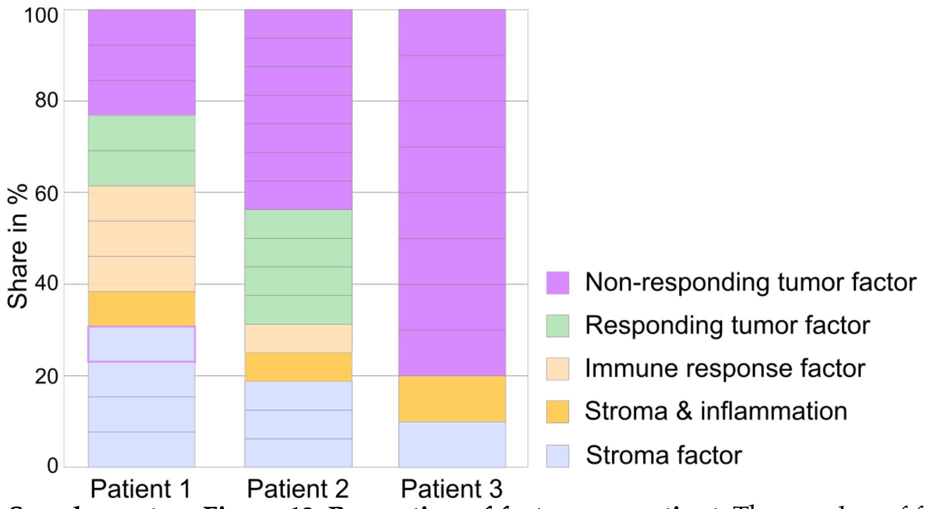

Supplementary Figure 13: Proportion of factors per patient. The number of factors per stroma, stroma & inflammation, immune response, responding tumor, and non-responding tumor factor indicates the diversity of expression profiles corresponds with the treatment response. Encircled stroma factor indicates non-responsive characteristics. Source data is provided as Source Data file.

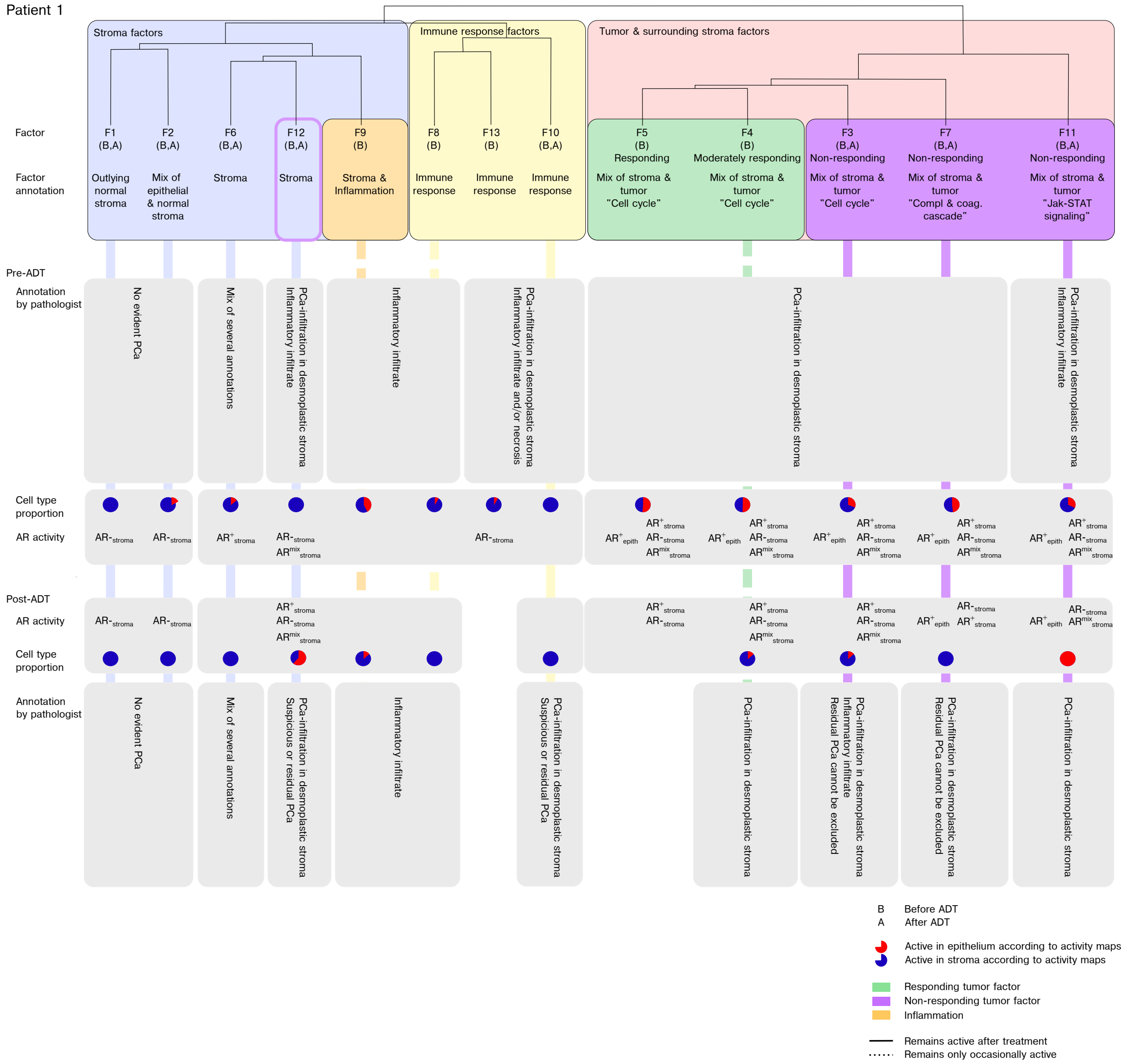

Supplementary Figure 14: Tree using hierarchical clustering of STD transcriptomic factors for patient 1. Stroma/immune response and tumor factors divide into different clusters. Stroma factors separate from the immune response factors. Non-responding tumor factors (purple) separate from the responding tumor factor (green). Circles indicate the ratio of epithelial and stroma spots as representative of the active transcriptomic factors. Each transcriptomic factor was mapped to an annotation and to the AR activity in the nucleus. 'B' corresponds to presence before treatment and 'B/A' to presence before and after treatment. Source data is provided as Source Data file. Abbreviations: AR; androgen receptor, ADT; androgen deprivation therapy, F; factor, PCa; prostate cancer

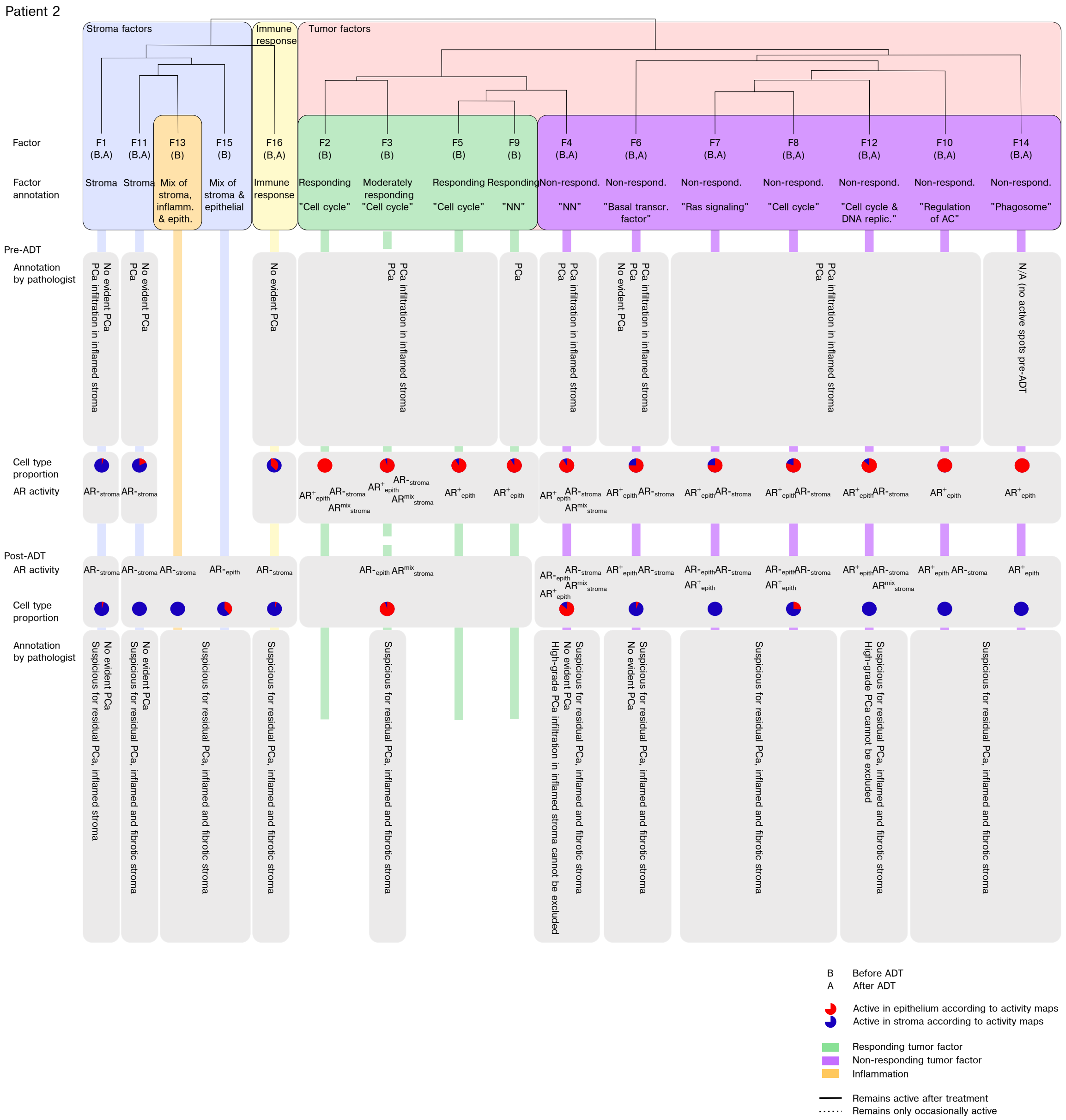

Supplementary Figure 15. Tree using hierarchical clustering of STD transcriptomic factors for patient 2. Stroma and tumor factors cluster into two different groups. Source data is provided as Source Data file. Source data is provided as Source Data file. Abbreviations: AR; androgen receptor, ADT; androgen deprivation therapy, F; factor, PCa; prostate cancer

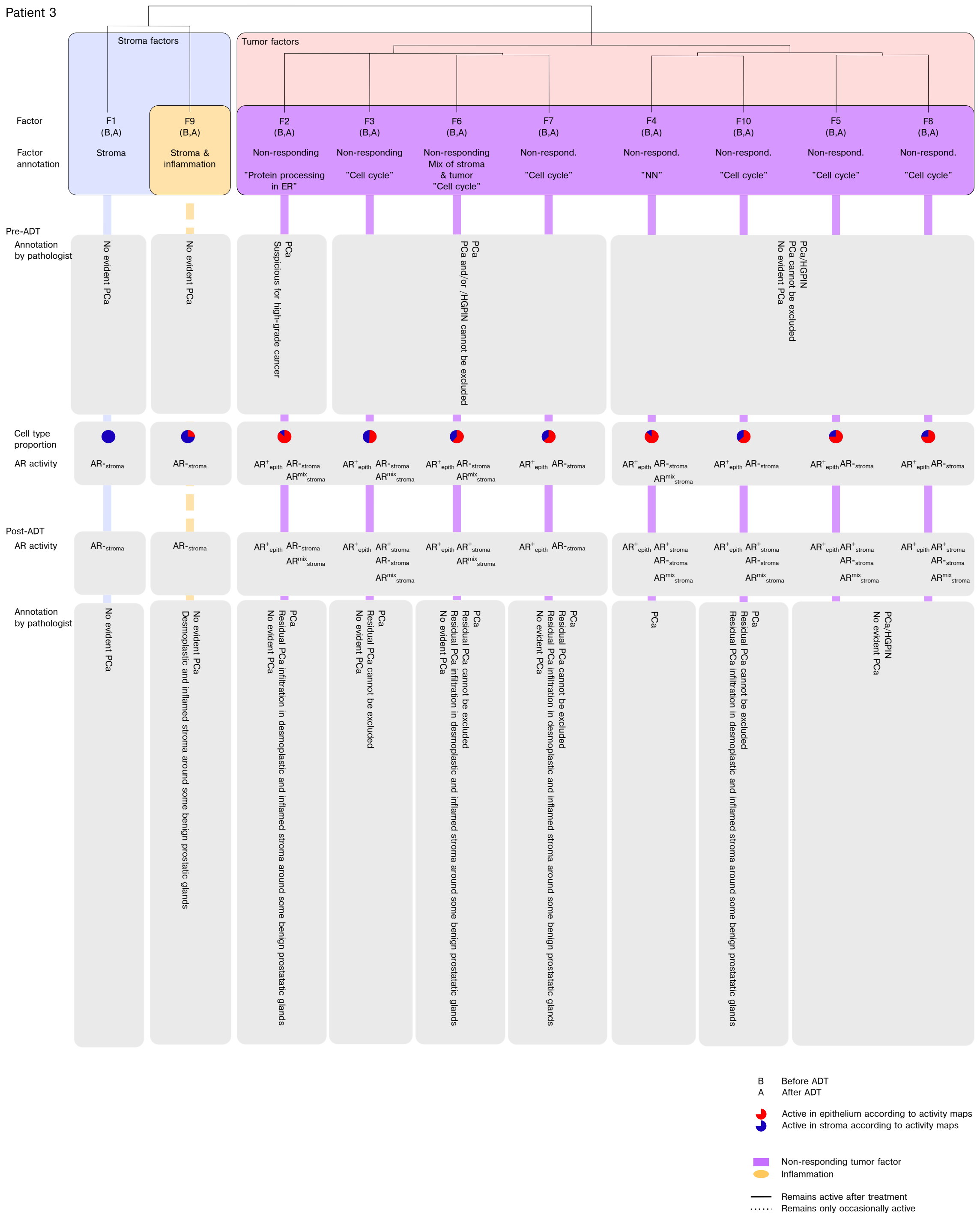

Supplementary Figure 16. Tree using hierarchical clustering of STD transcriptomic factors for patient 3. Stroma/immune related factors and tumor factors cluster into two different groups. There are two clusters of non-responding tumor factors (violet) each dominated by another pathologist annotation. Circles indicate the ratio of epithelial and stroma spots as representative of the active transcriptomic factors. Each transcriptomic factor was mapped to an annotation and to the nuclear AR activity. Source data is provided as Source Data file. Source data is provided as Source Data file. Abbreviations: AR; androgen receptor, ADT; androgen deprivation therapy, F; factor, PCa; prostate cancer

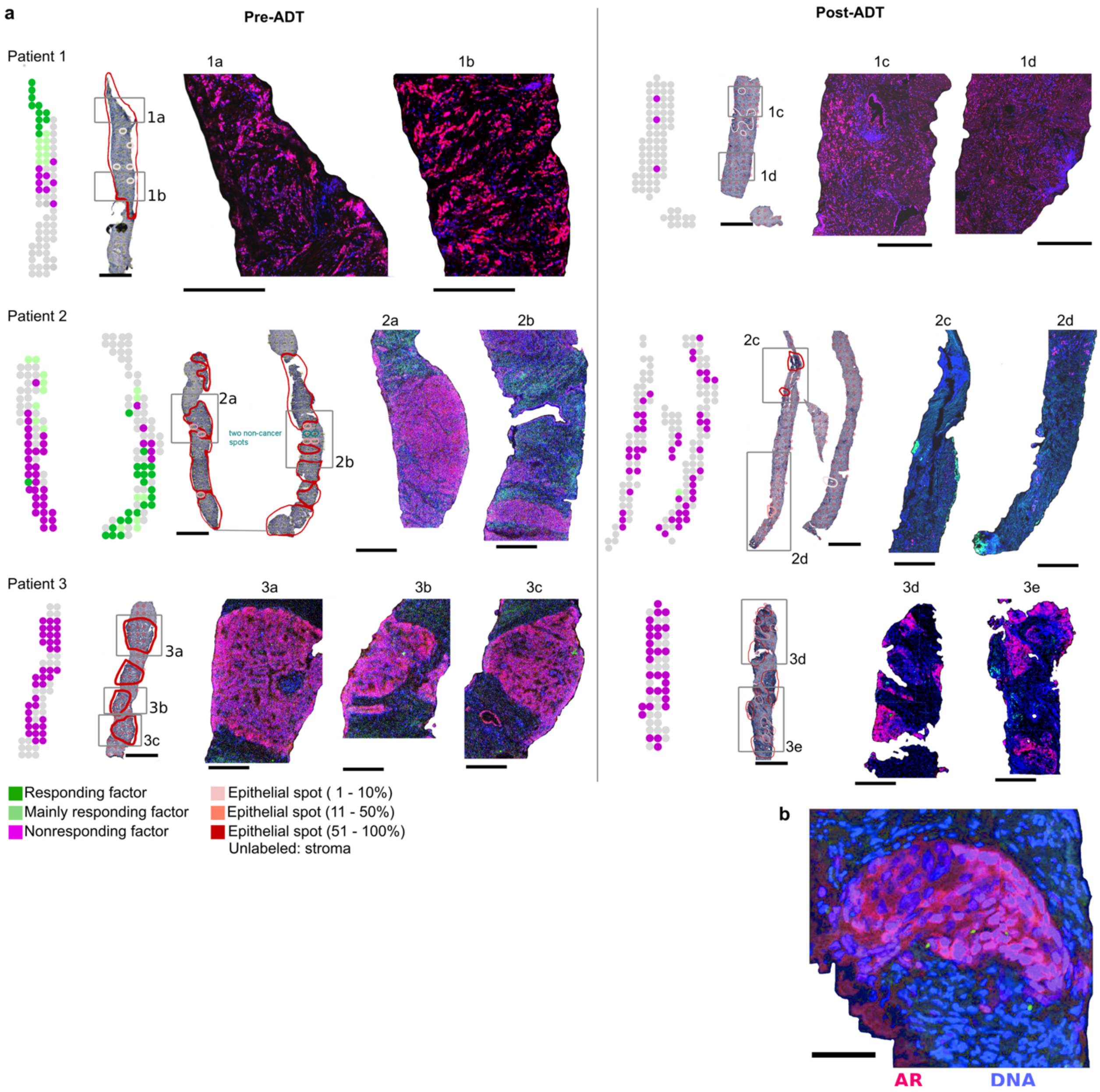

Supplementary Fig. 17: Correlation of cancer factors to AR activity. a, Examples of spatial distribution of spots, pre- and post-ADT, representative of responding and non-responding factors pre- and post-ADT. As expected, nuclear AR of epithelial cells pre-ADT was detected irrespectively of responsiveness, while nuclei AR of epithelial cells post-ADT was only seen in non-responsive tissue areas. The core needle biopsies of patient 2 were originally connected on the bottom. Scale bars on HE images corresponds to 1mm and bars on immunostained close ups corresponds to 500 µm. **b,** Zoom-in immunostained image showing that the AR of epithelial cells is nuclear. Scale bar corresponds to 10 µm. The bars in the close ups correspond to 200 µm.

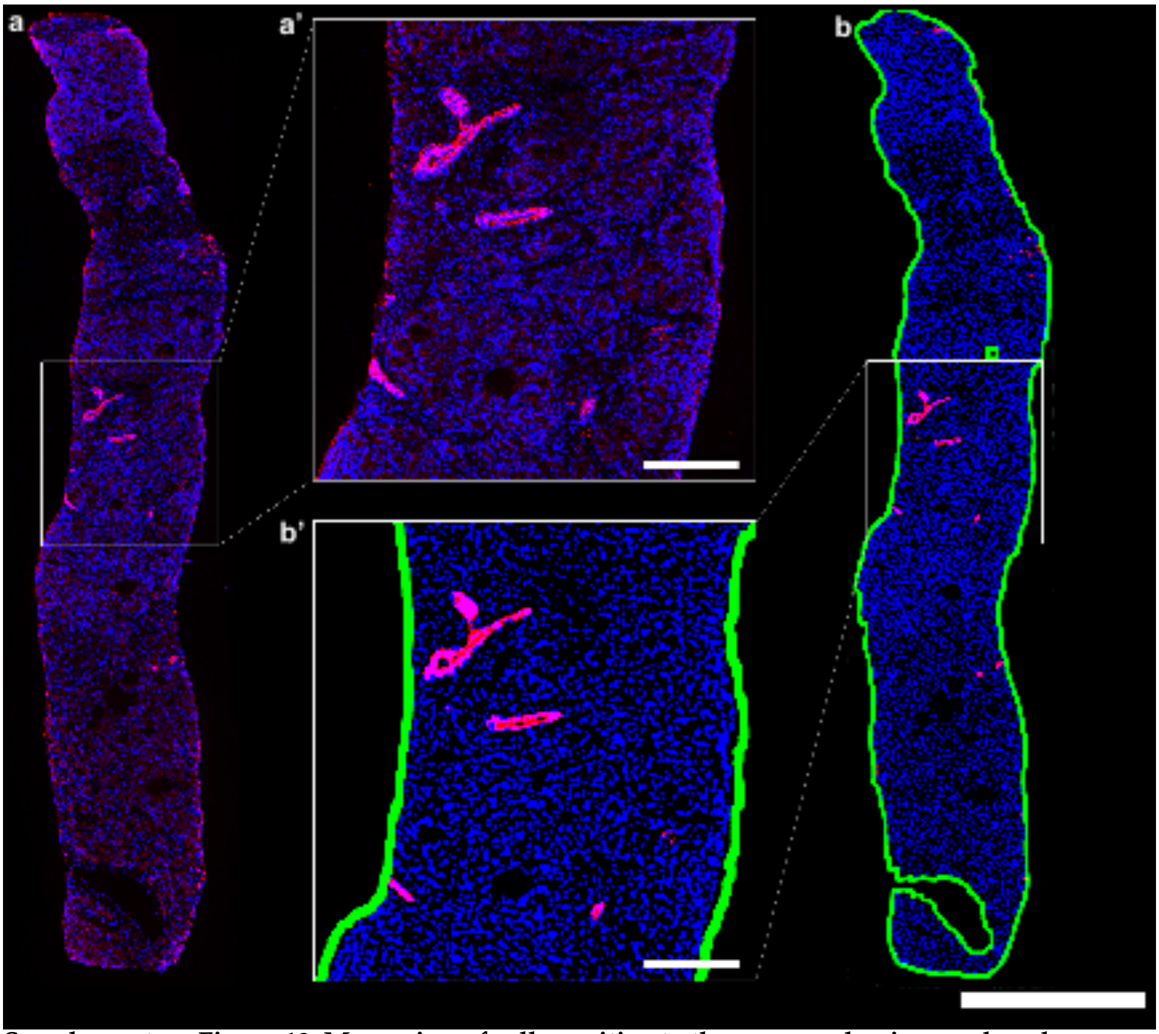

Supplementary Figure 18. Measuring of cells positive to the neuroendocrine marker chromogranin A. a, A biopsy from patient 2. DNA is depicted in blue and chromogranin A in red. A close up of the white square on the biopsy is shown in the upper mid and named a′. The scale bar in the figure corresponds to 1 mm and the bars in the close ups correspond to 200 um. **b,** The processed image used for measurement of DNA area and chromogranin A area. The borders of the biopsy are eroded by the algorithm (shown in green) to avoid the nonspecific antibody binding that often occurs at the tissue rim.

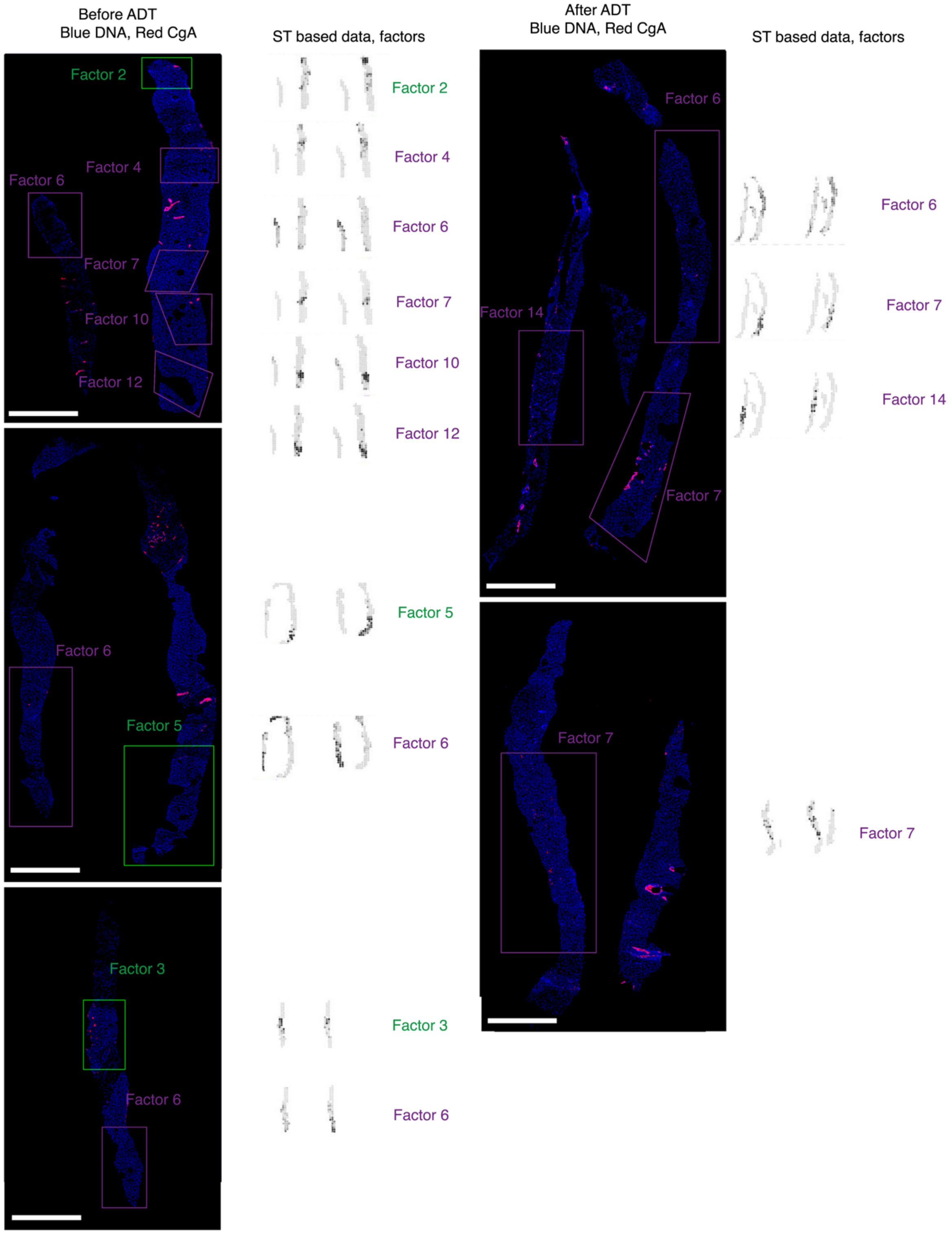

Supplementary Figure 19. Measuring of the fraction of cells positive to the neuroendocrine marker chromograninA (CgA) in biopsy areas. Factors shown in green correspond to non-resistant cancer while factors in purple correspond to resistant cancer. Areas with at least five spots with a given factor clustered together in the same area in both of the ST replicas were depicted for analyses. The Ratio of the CgA area and DNA area in these areas was measured and expressed as percentages of CgA area of the DNA area (Supplementary table 3 and 4) Normally the area of the cytoplasm is approximately three times the area of the nucleus. Thus, to get an estimation of the percentages of CgA positive cells, the numbers should be divided by 3. The length of the scale bars are 1 mm.

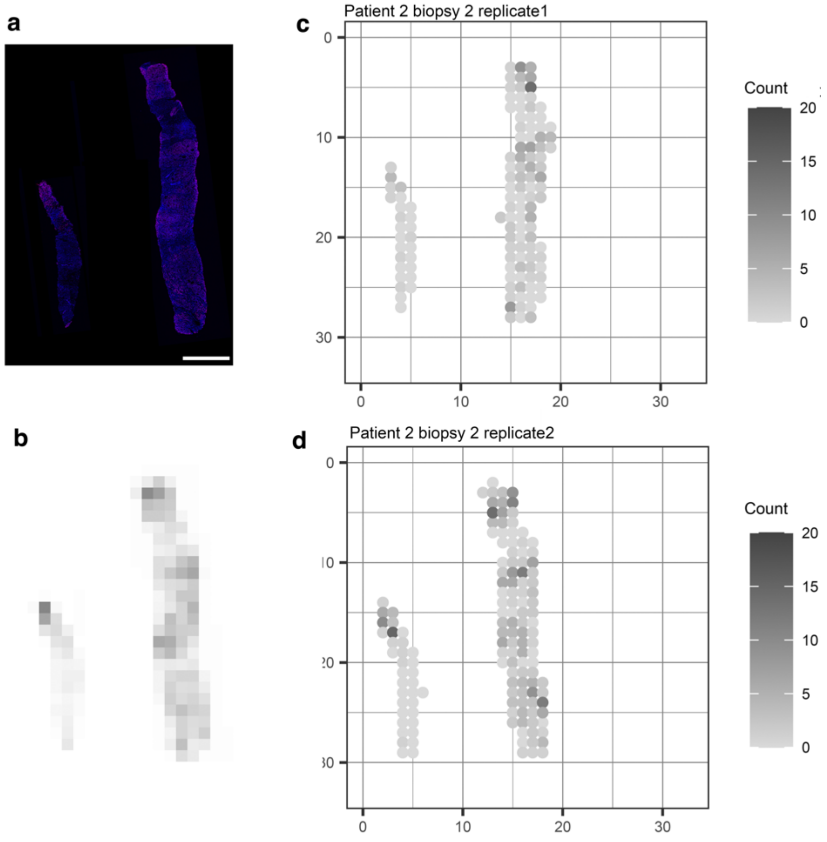

Supplementary Figure 20. Comparing the RNA expression of the androgen receptor (AR) with the amount of AR protein. a, An immunostaining of a prostate biopsy stained for AR-red and DNA- blue. Scale bar, 1 mm. **b,** The signal of the AR transformed to a heatmap with the same resolution as the ST data. **c,** A heat map of the ST data for AR for replicate 1, and **d**, for replicate 2. The immunostained section was cut after replicate 2 and is therefore spatially closest to that section. Accordingly, the coherence between the RNA expression and amount of protein is best for replicate 2.

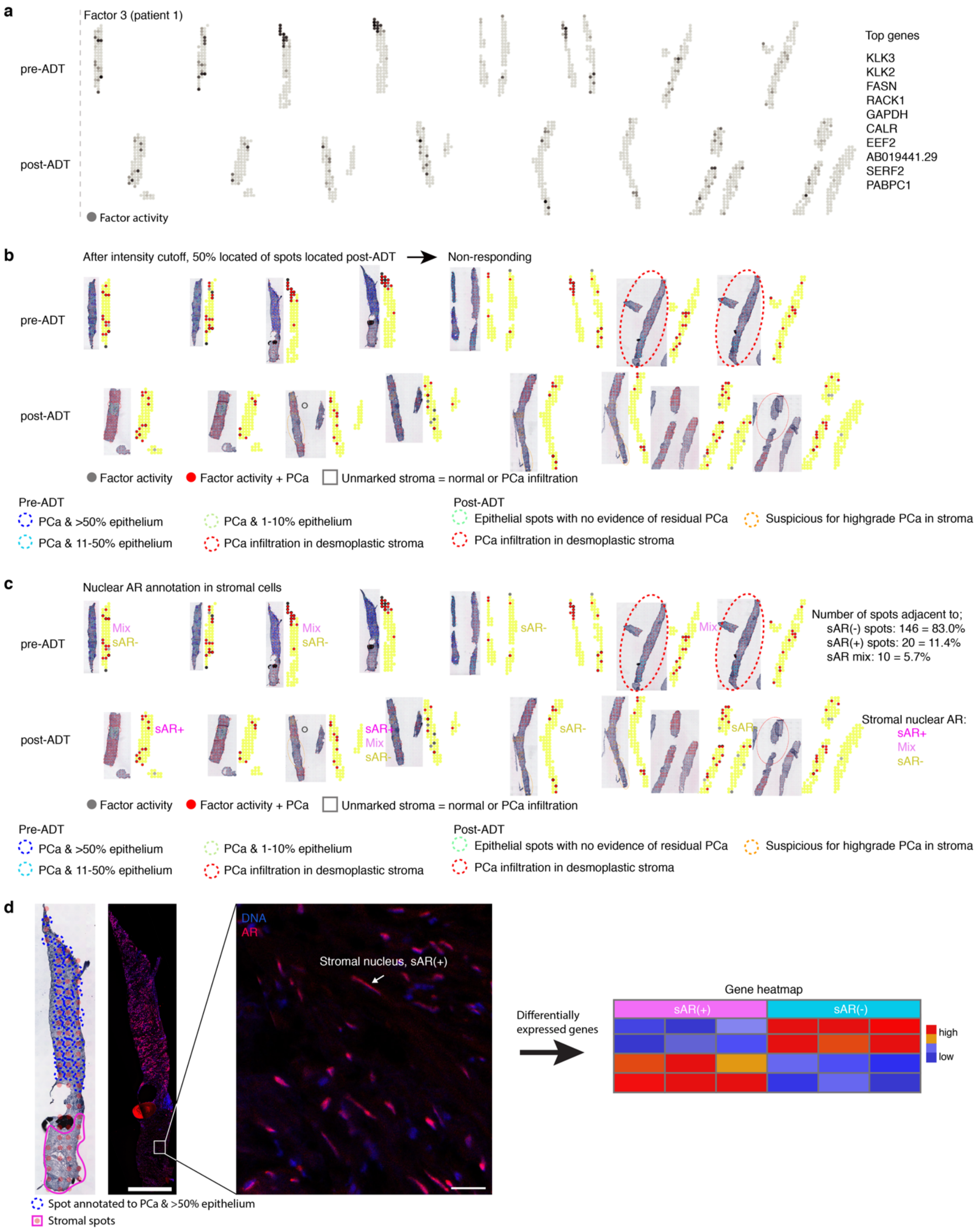

Supplementary Figure 21: Representative example of annotation of tumor factor and procedure for androgen receptor-based annotation in stromal cells. a, Raw activity map of tumor factor prior intensity cutoff. **b,** The same tumor factor is refined by applying an intensity-based cutoff. For annotation of responding or non-responding spots, the fraction of spots with activity post-ADT were calculated. For this calculation, only spots that remained after the cutoff, and were annotated to PCa, and contained >11% epithelial cells/stromal spots annotated to 'PCa infiltration in desmoplastic stroma', were counted as spots pre-ADT, while post-ADT, all spots were included. For the DGE analysis of responding versus non-responding spots, only spots pre-ADT were included (see Methods for further details). **c,** Adjacent stromal areas to each tumor factor spot were then evaluated for AR status (sAR(+), sAR(-), and sAR(mix)) and summarized. **d,** The transcriptomics data from stromal spot clusters with nuclei AR-positive and nuclei AR-negative status, adjacent to PCa regions, were used for DGE analysis. The image exemplifies AR-status in biopsy 2, pre-ADT (patient 1), which is sAR(+). Scale bar in whole figure, 1 mm, scale bars in close up, 10 μm. Abbreviations: ADT; androgen deprivation therapy, sAR(+); stromal nuclear AR positive, sAR(-); stromal nuclear AR negative, PCa; prostate cancer

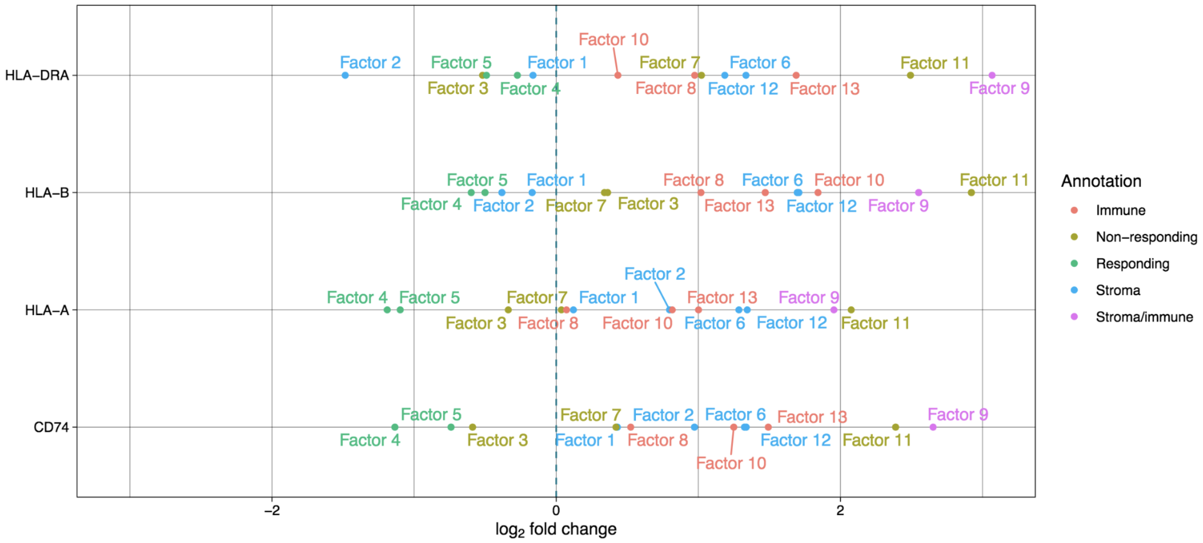

Supplementary Figure 22: Control that gene contributions were factor specific. Genes HLA-DRA, HLA-B, HLA-A and CD74 that were upregulated in spots from areas with non-responding factors in patient 1 were shown to be factor-specific, as opposed to confounding non-factor-specific genes, by comparing the contribution of these genes in all factors of patient 1. It can be seen that these genes are upregulated in non-responding factor 11, relative to the responding areas. Source data is provided as Source Data file.

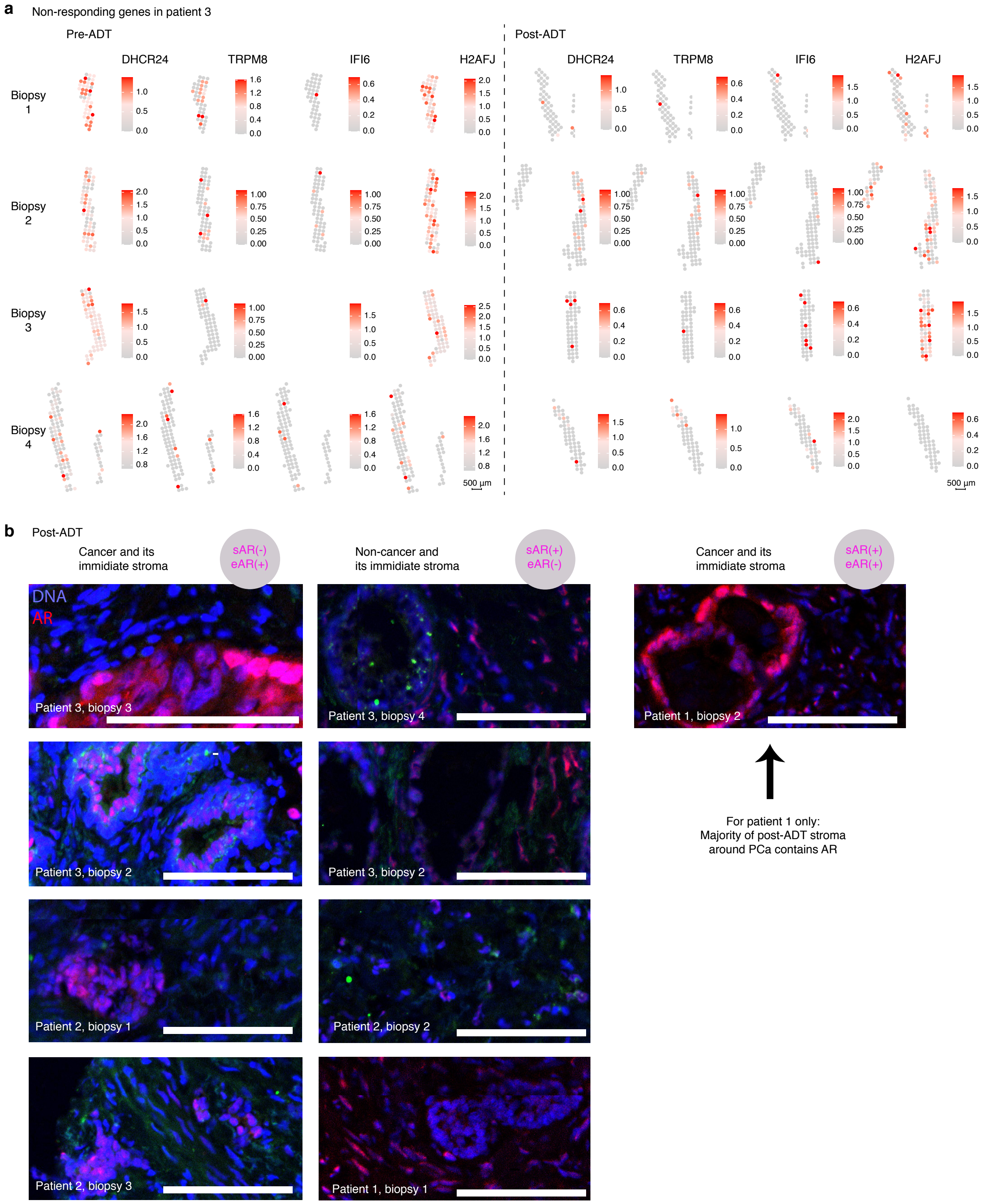

Supplementary Figure 23: Responding and non-responding tumor areas. a, Examples of genes upregulated in non-responding factors in patient 2 plotted onto the tissue sections of patient 3. **b,** Representative images of AR staining in stromal and epithelial areas, post-ADT, in the three patients. Stromal AR (sAR) and epithelial AR (eAR) have been denoted with +/-. Scale bars 100 µm.

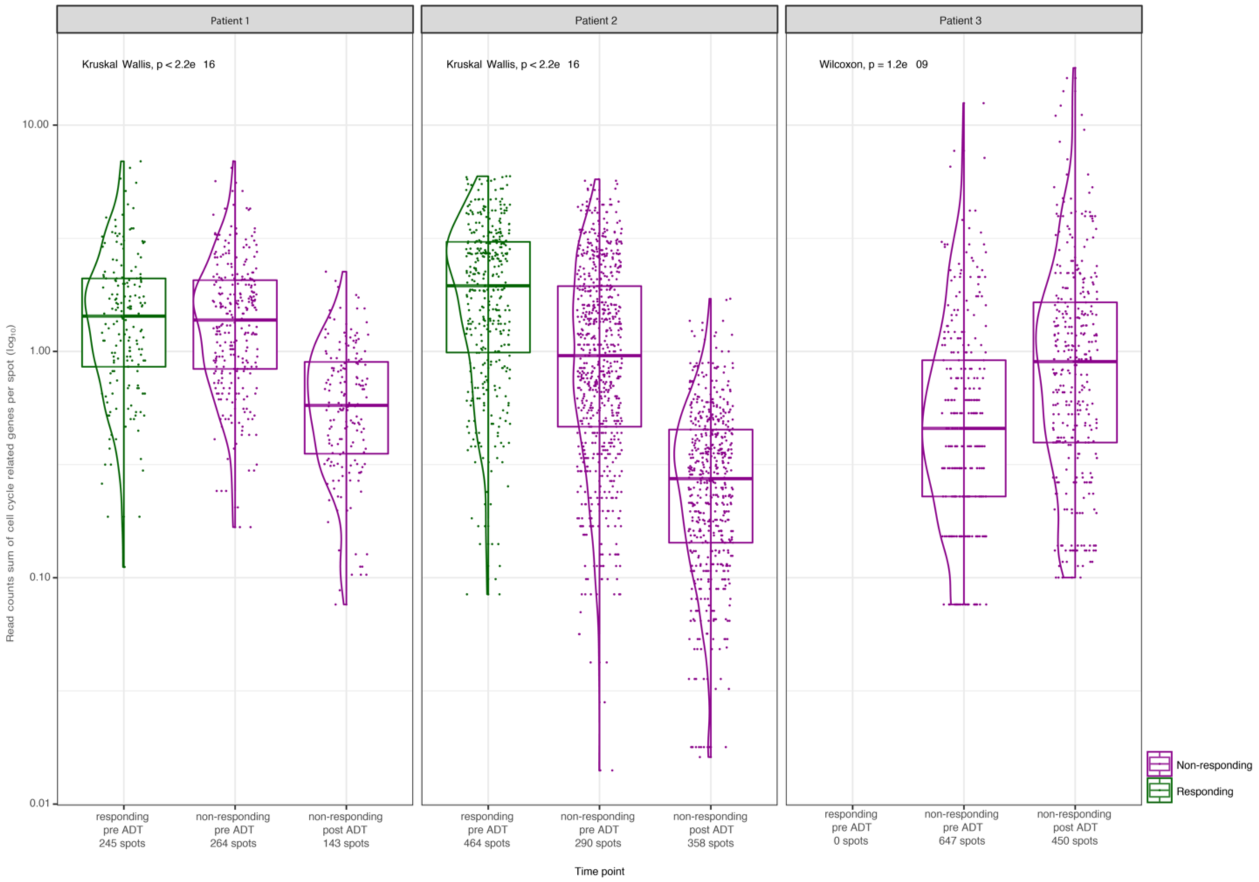

Supplementary Figure 24: Comparison of the summed read counts of the 71 cell cycle gene signature in non-responding cell clusters pre- and post- ADT. Each data point represents a spot containing responsive or non-responsive cell clusters either pre- or post-ADT. The boxes show the median and the quartiles of the dataset while the whiskers show minimum and maximum value of the data. Source data is provided as Source Data file.

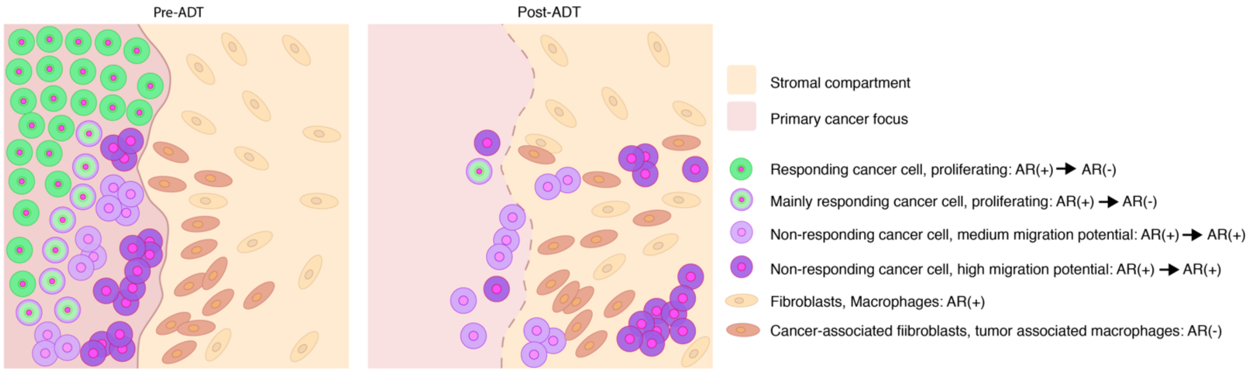

Supplementary Figure 25: A model of migration of non-responding cancer cells during the course of ADT-treatment and their crosstalk with the stroma compartment. The tumor cells acquire migratory traits for dissemination to distant organs. Abbreviations: AR; androgen receptor, ADT; androgen deprivation therapy, AR(+); AR positive, AR(-); AR negative
