# Supplementary material for: Spatio-temporal analysis of prostate tumors in situ suggests pre-existence of treatment-resistant clones
Source: Nat Commun. 2022 Sep 17;13:5475. doi: 10.1038/s41467-022-33069-3 (PMC9482614; doi:10.1038/s41467-022-33069-3)
Supplement: Supplementary file 3 — Description to Additional Supplementary Information [file 41467_2022_33069_MOESM3_ESM.pdf]

Description of Additional Supplementary Files

Supplementary_Data_File1_STD_Patient1_Act ivityMap_13_Factors: PDFs with ActivityMaps from the STD results of patient 1

Supplementary_Data_File2_STD_Patient1_Ge nes_13_Factors: PDFs with gene lists for factors from the STD results of patient 1

Supplementary_Data_File3_STD Patient2_ActivityMap_16_Factors: PDFs with ActivityMaps from the STD results of patient 2

Supplementary_Data_File4_STD_Patient2_Ge nes_16_Factors: PDFs with gene lists for factors from the STD results of patient 2

Supplementary_Data_File5_STD_Patient3_Act ivityMap_10_Factors: PDFs with ActivityMaps from the STD results of patient 3

Supplementary_Data_File6_STD_Patient3_Ge nes_10_Factors: PDFs with gene lists for factors from the STD results of patient 3
