# Supplementary material for: Spatio-temporal analysis of prostate tumors in situ suggests pre-existence of treatment-resistant clones
Source: Nat Commun. 2022 Sep 17;13:5475. doi: 10.1038/s41467-022-33069-3 (PMC9482614; doi:10.1038/s41467-022-33069-3)

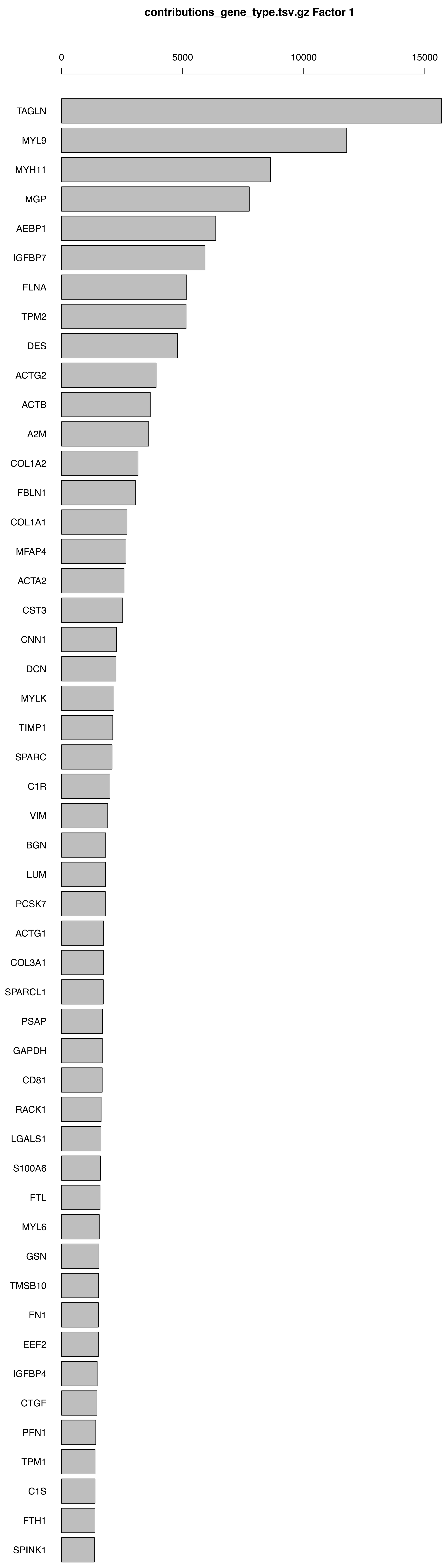
contributions_gene_type.tsv.gz Factor 1
0
5000
10000
15000
TAGLN
MYL9
MYH11
MGP
AEBP1
IGFBP7
FLNA
TPM2
DES
ACTG2
ACTB
A2M
COL1A2
FBLN1
COL1A1
MFAP4
ACTA2
CST3
CNN1
DCN
MYLK
TIMP1
SPARC
C1R
VIM
BGN
LUM
PCSK7
ACTG1
COL3A1
SPARCL1
PSAP
GAPDH
CD81
RACK1
LGALS1
S100A6
FTL
MYL6
GSN
TMSB10
FN1
EEF2
IGFBP4
CTGF
PFN1
TPM1
C1S
FTH1
SPINK1

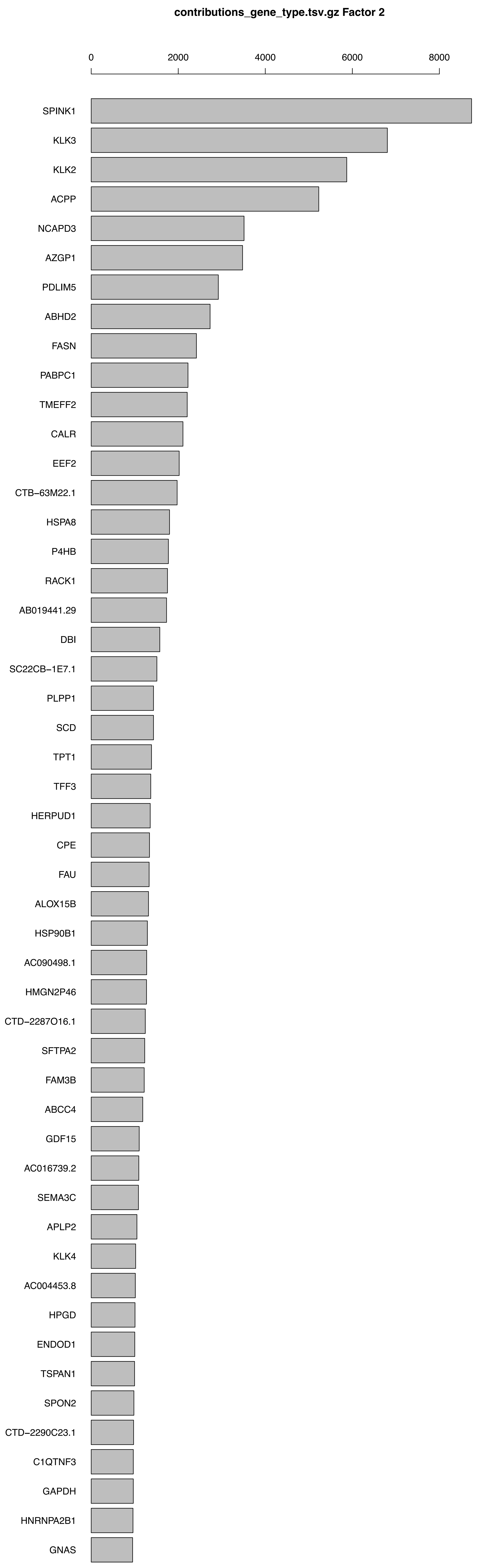
contributions_gene_type.tsv.gz Factor 2
0
2000
4000
6000
8000
SPINK1
KLK3
KLK2
ACPP
NCAPD3
AZGP1
PDLIM5
ABHD2
FASN
PABPC1
TMEFF2
CALR
EEF2
CTB−63M22.1
HSPA8
P4HB
RACK1
AB019441.29
DBI
SC22CB−1E7.1
PLPP1
SCD
TPT1
TFF3
HERPUD1
CPE
FAU
ALOX15B
HSP90B1
AC090498.1
HMGN2P46
CTD−2287O16.1
SFTPA2
FAM3B
ABCC4
GDF15
AC016739.2
SEMA3C
APLP2
KLK4
AC004453.8
HPGD
ENDOD1
TSPAN1
SPON2
CTD−2290C23.1
C1QTNF3
GAPDH
HNRNPA2B1
GNAS

contributions_gene_type.tsv.gz Factor 3

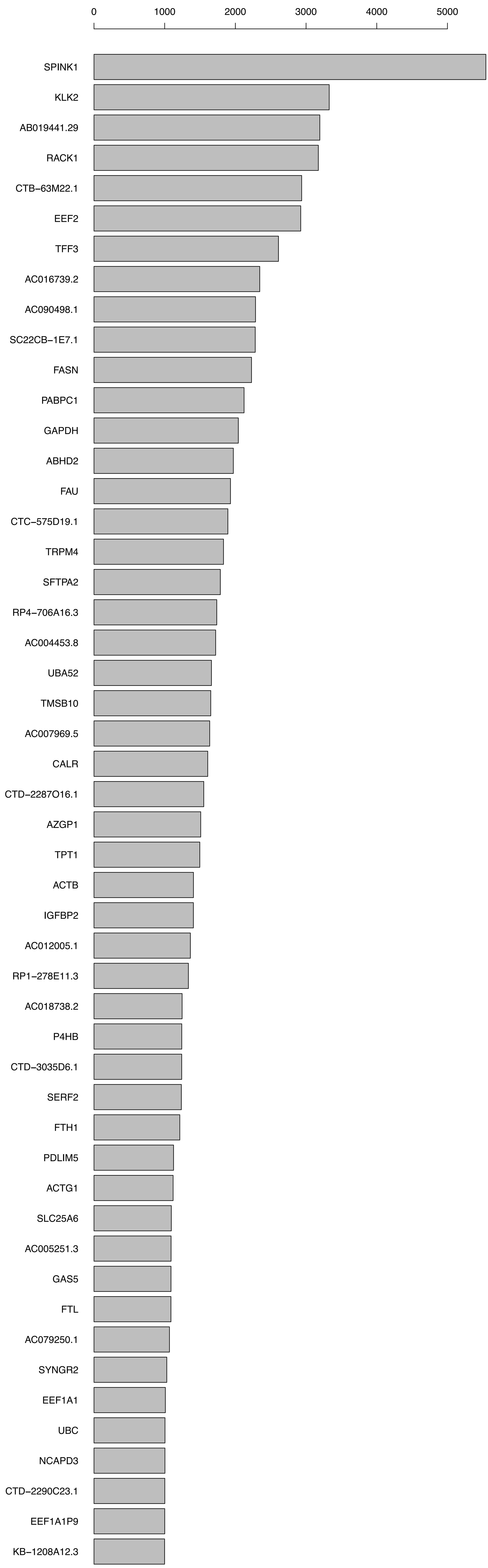

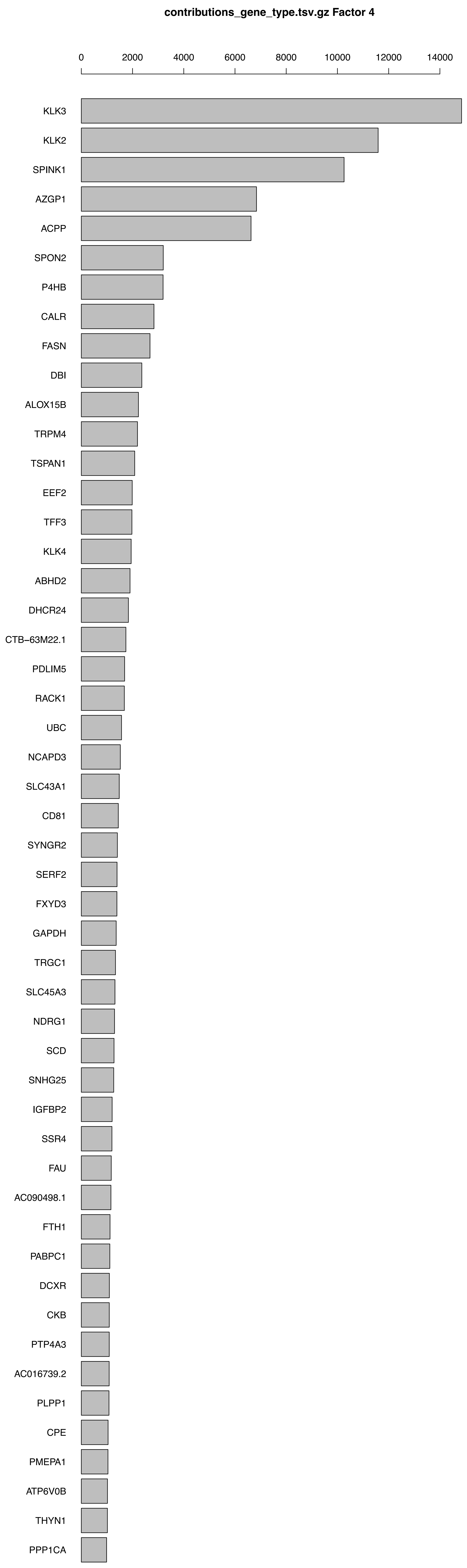
contributions_gene_type.tsv.gz Factor 4
0
2000
4000
6000
8000
10000
12000
14000
KLK3
KLK2
SPINK1
AZGP1
ACPP
SPON2
P4HB
CALR
FASN
DBI
ALOX15B
TRPM4
TSPAN1
EEF2
TFF3
KLK4
ABHD2
DHCR24
CTB−63M22.1
PDLIM5
RACK1
UBC
NCAPD3
SLC43A1
CD81
SYNGR2
SERF2
FXYD3
GAPDH
TRGC1
SLC45A3
NDRG1
SCD
SNHG25
IGFBP2
SSR4
FAU
AC090498.1
FTH1
PABPC1
DCXR
CKB
PTP4A3
AC016739.2
PLPP1
CPE
PMEPA1
ATP6V0B
THYN1
PPP1CA

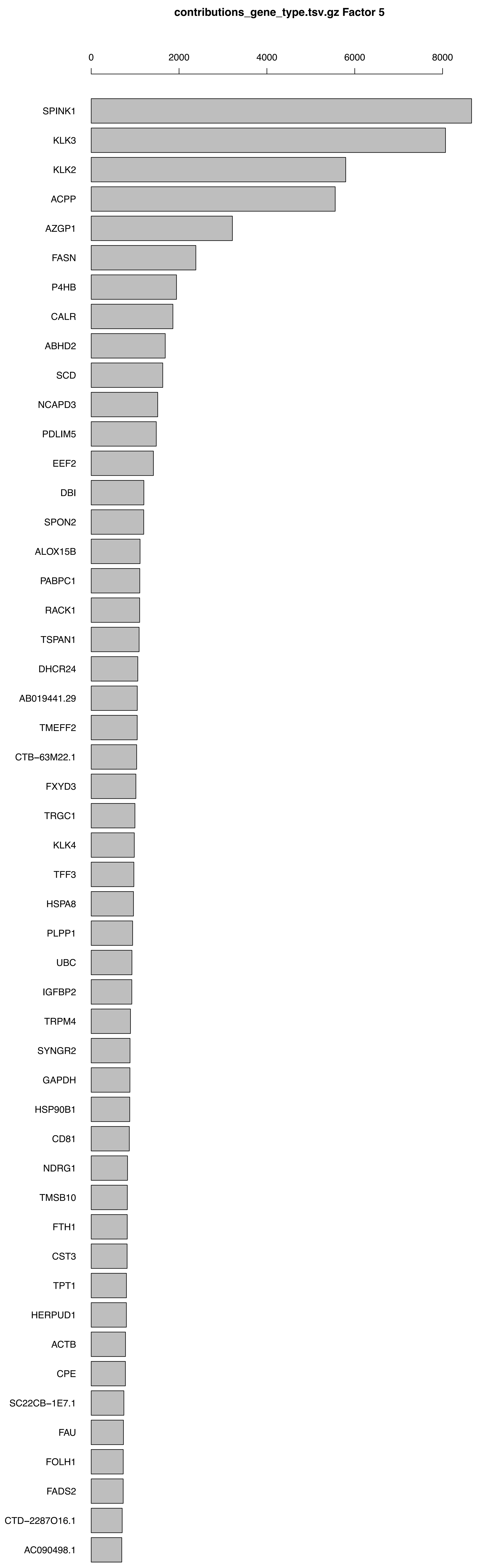
contributions_gene_type.tsv.gz Factor 5
0
2000
4000
6000
8000
SPINK1
KLK3
KLK2
ACPP
AZGP1
FASN
P4HB
CALR
ABHD2
SCD
NCAPD3
PDLIM5
EEF2
DBI
SPON2
ALOX15B
PABPC1
RACK1
TSPAN1
DHCR24
AB019441.29
TMEFF2
CTB−63M22.1
FXYD3
TRGC1
KLK4
TFF3
HSPA8
PLPP1
UBC
IGFBP2
TRPM4
SYNGR2
GAPDH
HSP90B1
CD81
NDRG1
TMSB10
FTH1
CST3
TPT1
HERPUD1
ACTB
CPE
SC22CB−1E7.1
FAU
FOLH1
FADS2
CTD−2287O16.1
AC090498.1

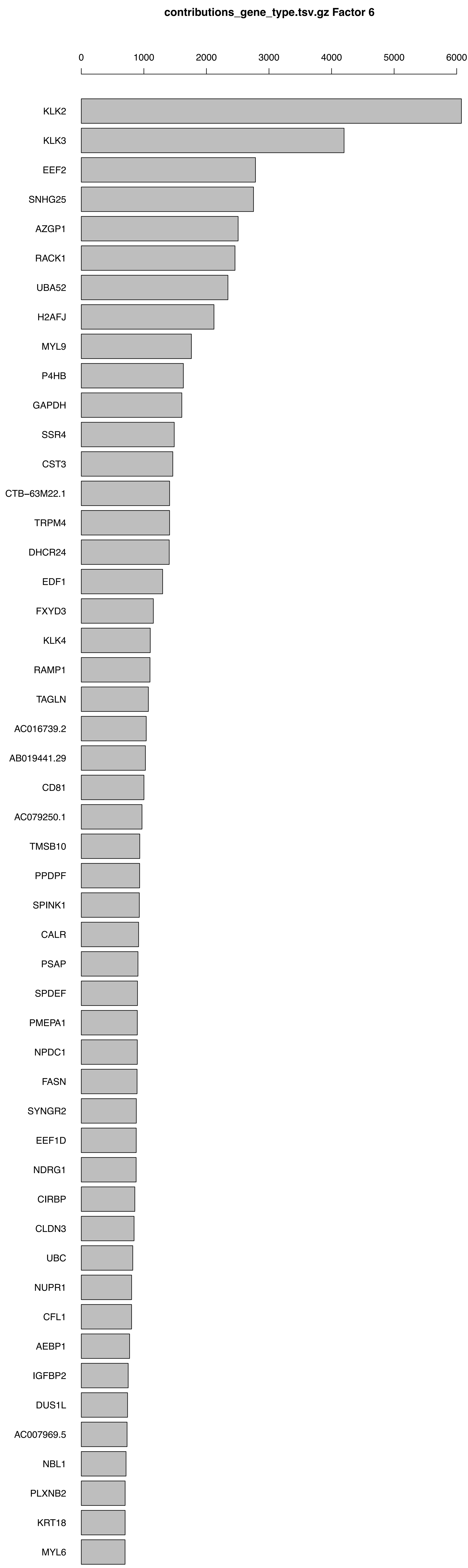
contributions_gene_type.tsv.gz Factor 6
0
1000
2000
3000
4000
5000
6000
KLK2
KLK3
EEF2
SNHG25
AZGP1
RACK1
UBA52
H2AFJ
MYL9
P4HB
GAPDH
SSR4
CST3
CTB−63M22.1
TRPM4
DHCR24
EDF1
FXYD3
KLK4
RAMP1
TAGLN
AC016739.2
AB019441.29
CD81
AC079250.1
TMSB10
PPDPF
SPINK1
CALR
PSAP
SPDEF
PMEPA1
NPDC1
FASN
SYNGR2
EEF1D
NDRG1
CIRBP
CLDN3
UBC
NUPR1
CFL1
AEBP1
IGFBP2
DUS1L
AC007969.5
NBL1
PLXNB2
KRT18
MYL6

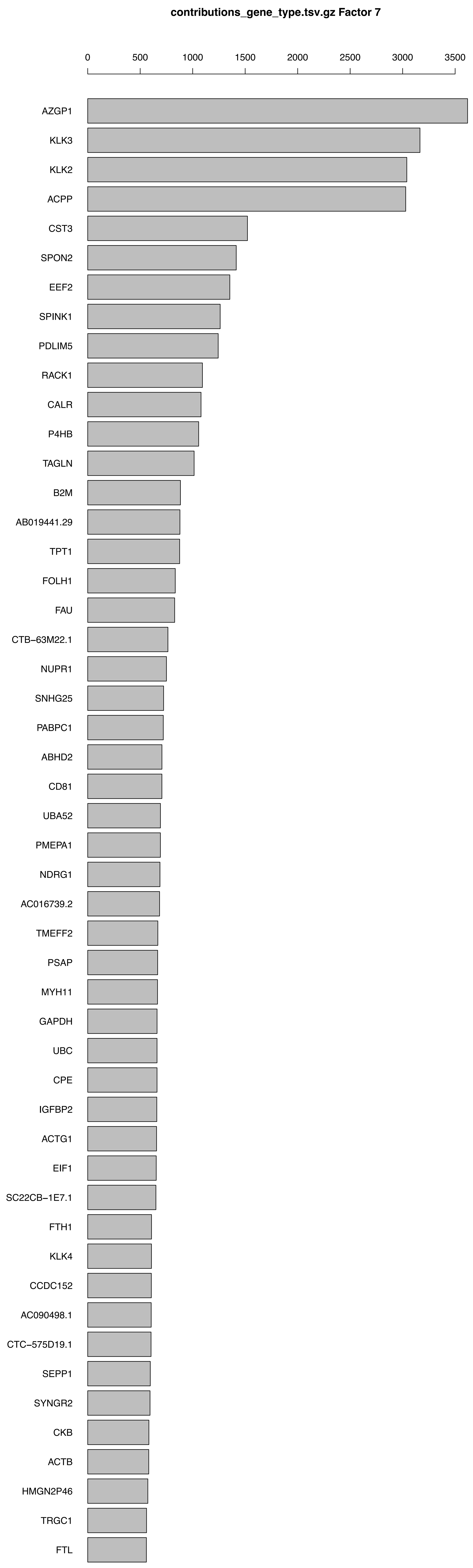
contributions_gene_type.tsv.gz Factor 7
0
500
1000
1500
2000
2500
3000
3500
AZGP1
KLK3
KLK2
ACPP
CST3
SPON2
EEF2
SPINK1
PDLIM5
RACK1
CALR
P4HB
TAGLN
B2M
AB019441.29
TPT1
FOLH1
FAU
CTB−63M22.1
NUPR1
SNHG25
PABPC1
ABHD2
CD81
UBA52
PMEPA1
NDRG1
AC016739.2
TMEFF2
PSAP
MYH11
GAPDH
UBC
CPE
IGFBP2
ACTG1
EIF1
SC22CB−1E7.1
FTH1
KLK4
CCDC152
AC090498.1
CTC−575D19.1
SEPP1
SYNGR2
CKB
ACTB
HMGN2P46
TRGC1
FTL

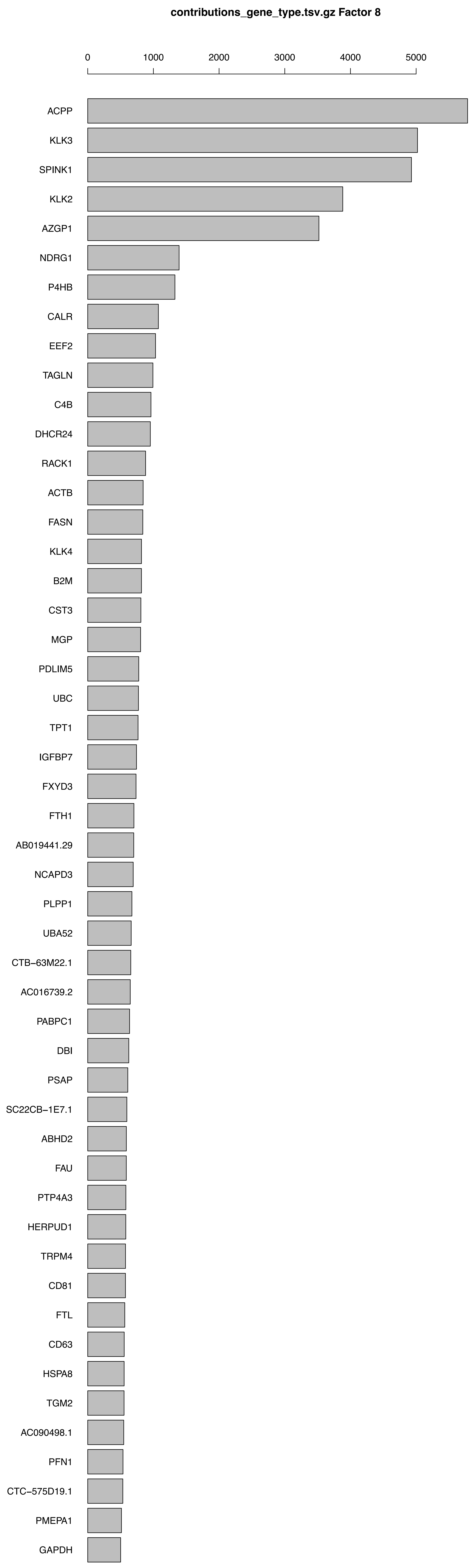
contributions_gene_type.tsv.gz Factor 8
0
1000
2000
3000
4000
5000
ACPP
KLK3
SPINK1
KLK2
AZGP1
NDRG1
P4HB
CALR
EEF2
TAGLN
C4B
DHCR24
RACK1
ACTB
FASN
KLK4
B2M
CST3
MGP
PDLIM5
UBC
TPT1
IGFBP7
FXYD3
FTH1
AB019441.29
NCAPD3
PLPP1
UBA52
CTB−63M22.1
AC016739.2
PABPC1
DBI
PSAP
SC22CB−1E7.1
ABHD2
FAU
PTP4A3
HERPUD1
TRPM4
CD81
FTL
CD63
HSPA8
TGM2
AC090498.1
PFN1
CTC−575D19.1
PMEPA1
GAPDH

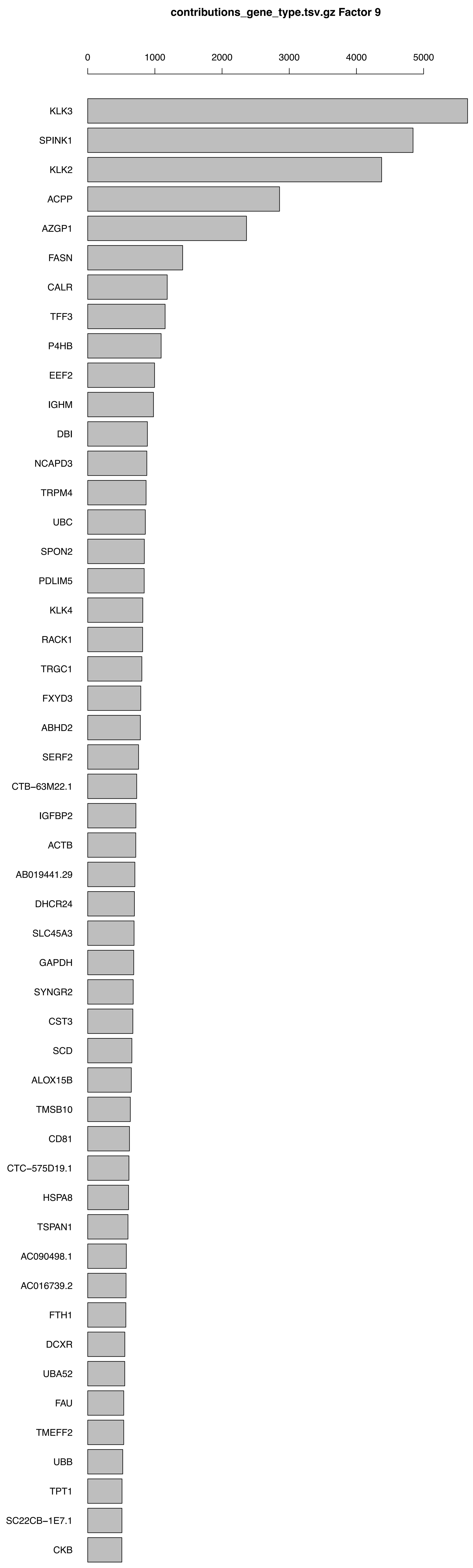

contributions_gene_type.tsv.gz Factor 9
0
1000
2000
3000
4000
5000
KLK3
SPINK1
KLK2
ACPP
AZGP1
FASN
CALR
TFF3
P4HB
EEF2
IGHM
DBI
NCAPD3
TRPM4
UBC
SPON2
PDLIM5
KLK4
RACK1
TRGC1
FXYD3
ABHD2
SERF2
CTB−63M22.1
IGFBP2
ACTB
AB019441.29
DHCR24
SLC45A3
GAPDH
SYNGR2
CST3
SCD
ALOX15B
TMSB10
CD81
CTC−575D19.1
HSPA8
TSPAN1
AC090498.1
AC016739.2
FTH1
DCXR
UBA52
FAU
TMEFF2
UBB
TPT1
SC22CB−1E7.1
CKB

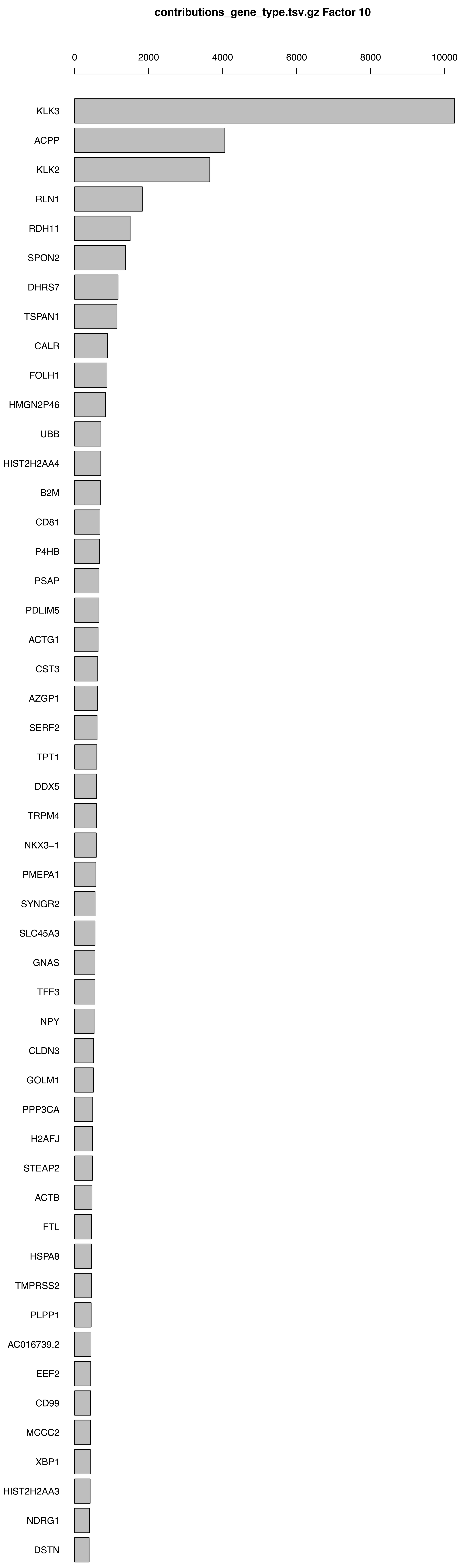
contributions_gene_type.tsv.gz Factor 10
0
2000
4000
6000
8000
10000
KLK3
ACPP
KLK2
RLN1
RDH11
SPON2
DHRS7
TSPAN1
CALR
FOLH1
HMGN2P46
UBB
HIST2H2AA4
B2M
CD81
P4HB
PSAP
PDLIM5
ACTG1
CST3
AZGP1
SERF2
TPT1
DDX5
TRPM4
NKX3−1
PMEPA1
SYNGR2
SLC45A3
GNAS
TFF3
NPY
CLDN3
GOLM1
PPP3CA
H2AFJ
STEAP2
ACTB
FTL
HSPA8
TMPRSS2
PLPP1
AC016739.2
EEF2
CD99
MCCC2
XBP1
HIST2H2AA3
NDRG1
DSTN

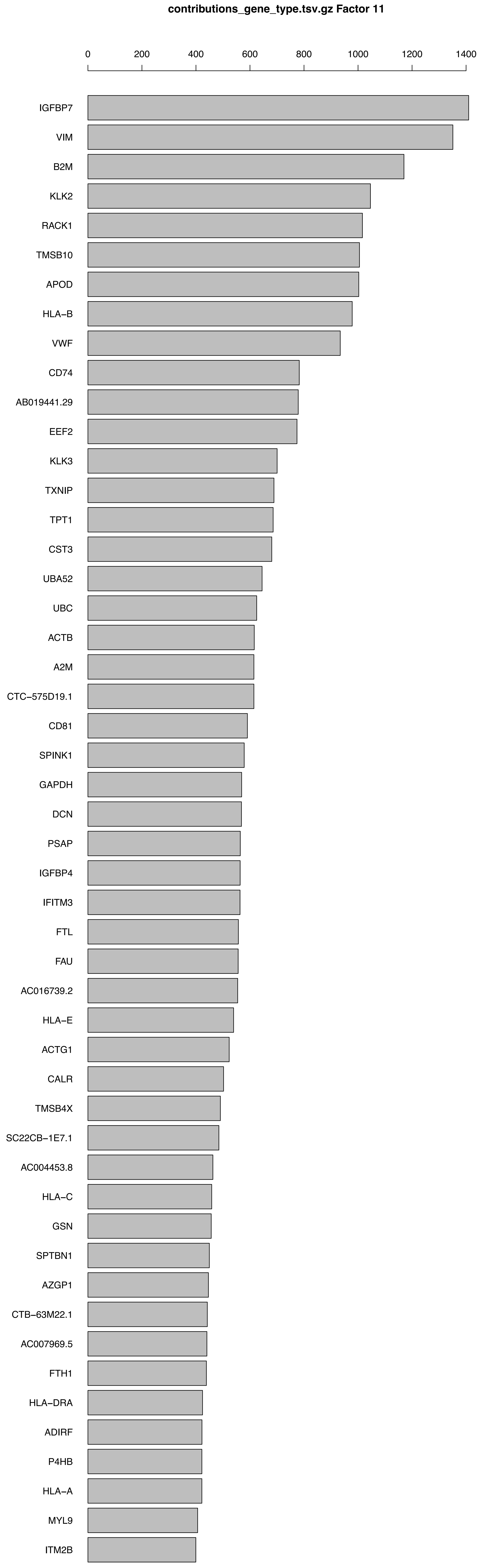
contributions_gene_type.tsv.gz Factor 11
0
200
400
600
800
1000
1200
1400
IGFBP7
VIM
B2M
KLK2
RACK1
TMSB10
APOD
HLA−B
VWF
CD74
AB019441.29
EEF2
KLK3
TXNIP
TPT1
CST3
UBA52
UBC
ACTB
A2M
CTC−575D19.1
CD81
SPINK1
GAPDH
DCN
PSAP
IGFBP4
IFITM3
FTL
FAU
AC016739.2
HLA−E
ACTG1
CALR
TMSB4X
SC22CB−1E7.1
AC004453.8
HLA−C
GSN
SPTBN1
AZGP1
CTB−63M22.1
AC007969.5
FTH1
HLA−DRA
ADIRF
P4HB
HLA−A
MYL9
ITM2B

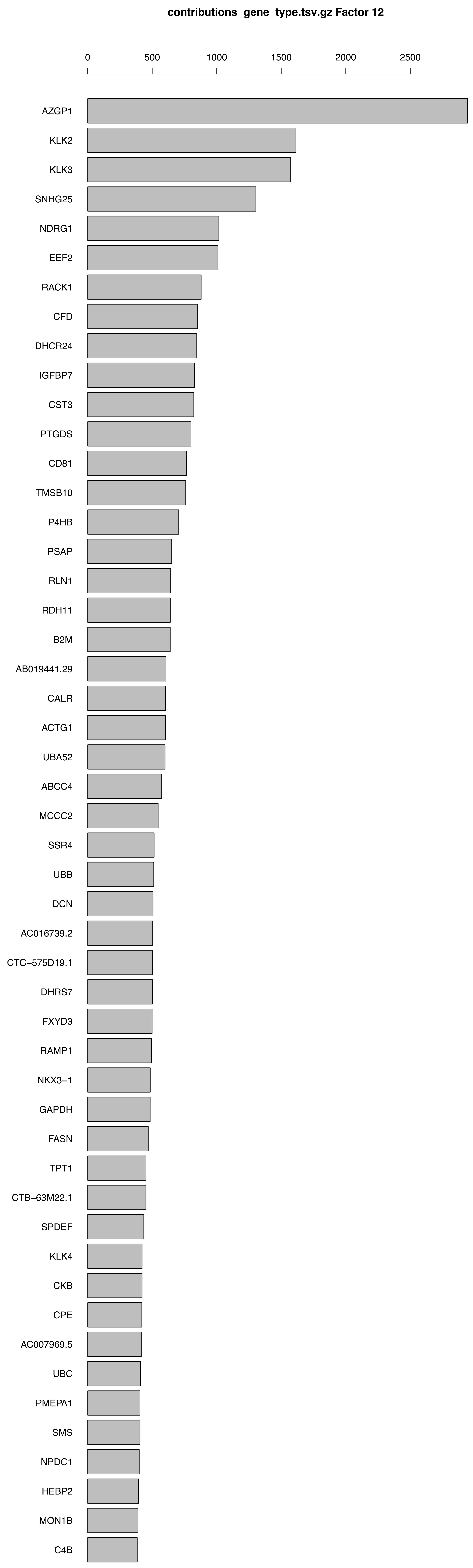
contributions_gene_type.tsv.gz Factor 12
0
500
1000
1500
2000
2500
AZGP1
KLK2
KLK3
SNHG25
NDRG1
EEF2
RACK1
CFD
DHCR24
IGFBP7
CST3
PTGDS
CD81
TMSB10
P4HB
PSAP
RLN1
RDH11
B2M
AB019441.29
CALR
ACTG1
UBA52
ABCC4
MCCC2
SSR4
UBB
DCN
AC016739.2
CTC−575D19.1
DHRS7
FXYD3
RAMP1
NKX3−1
GAPDH
FASN
TPT1
CTB−63M22.1
SPDEF
KLK4
CKB
CPE
AC007969.5
UBC
PMEPA1
SMS
NPDC1
HEBP2
MON1B
C4B

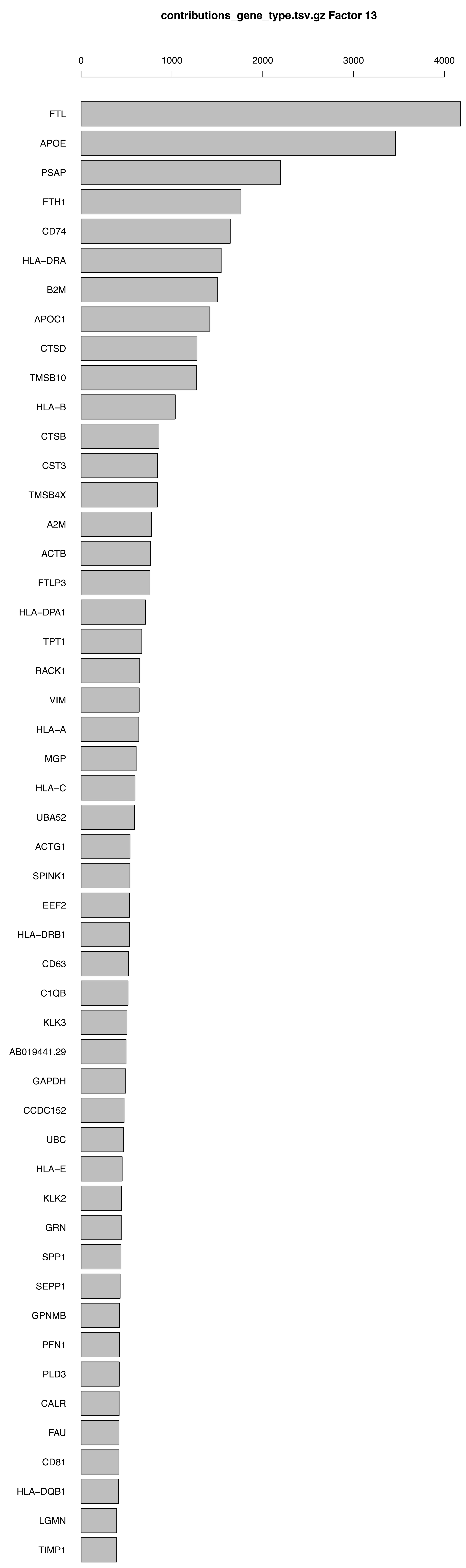
contributions_gene_type.tsv.gz Factor 13
0
1000
2000
3000
4000
FTL
APOE
PSAP
FTH1
CD74
HLA−DRA
B2M
APOC1
CTSD
TMSB10
HLA−B
CTSB
CST3
TMSB4X
A2M
ACTB
FTLP3
HLA−DPA1
TPT1
RACK1
VIM
HLA−A
MGP
HLA−C
UBA52
ACTG1
SPINK1
EEF2
HLA−DRB1
CD63
C1QB
KLK3
AB019441.29
GAPDH
CCDC152
UBC
HLA−E
KLK2
GRN
SPP1
SEPP1
GPNMB
PFN1
PLD3
CALR
FAU
CD81
HLA−DQB1
LGMN
TIMP1

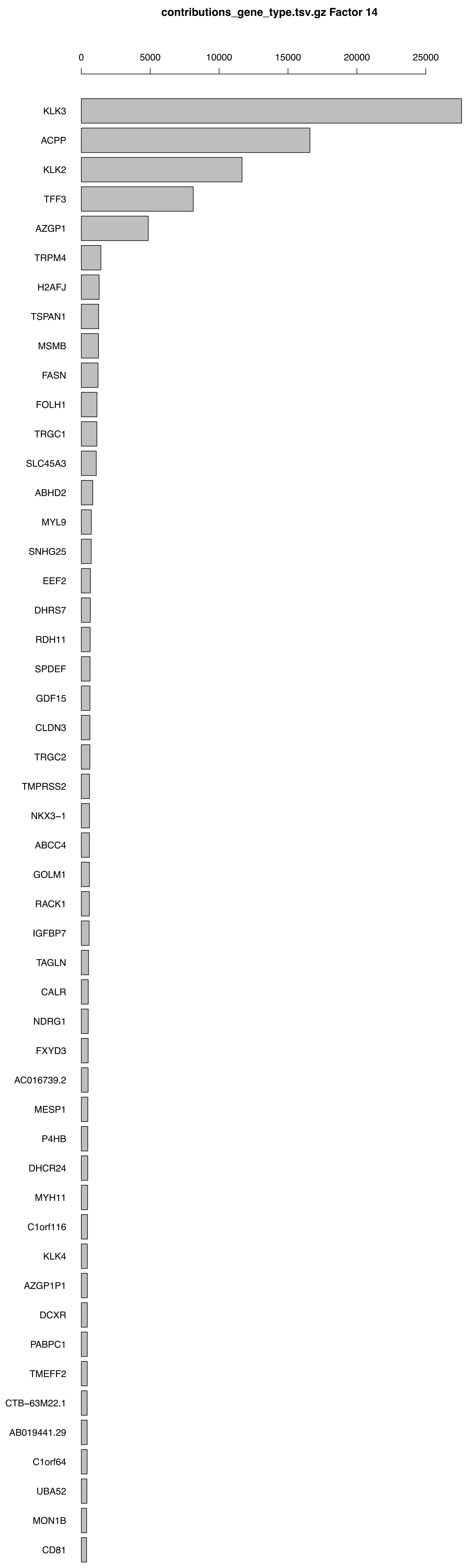
contributions_gene_type.tsv.gz Factor 14
0
5000
10000
15000
20000
25000
KLK3
ACPP
KLK2
TFF3
AZGP1
TRPM4
H2AFJ
TSPAN1
MSMB
FASN
FOLH1
TRGC1
SLC45A3
ABHD2
MYL9
SNHG25
EEF2
DHRS7
RDH11
SPDEF
GDF15
CLDN3
TRGC2
TMPRSS2
NKX3−1
ABCC4
GOLM1
RACK1
IGFBP7
TAGLN
CALR
NDRG1
FXYD3
AC016739.2
MESP1
P4HB
DHCR24
MYH11
C1orf116
KLK4
AZGP1P1
DCXR
PABPC1
TMEFF2
CTB−63M22.1
AB019441.29
C1orf64
UBA52
MON1B
CD81

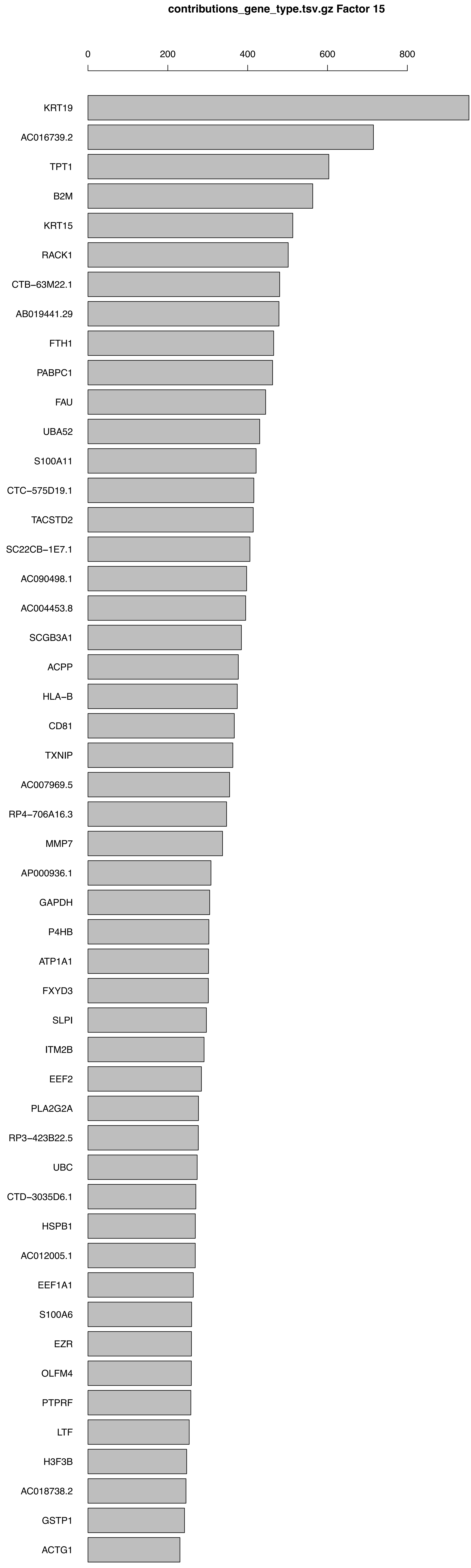
contributions_gene_type.tsv.gz Factor 15
0
200
400
600
800
KRT19
AC016739.2
TPT1
B2M
KRT15
RACK1
CTB−63M22.1
AB019441.29
FTH1
PABPC1
FAU
UBA52
S100A11
CTC−575D19.1
TACSTD2
SC22CB−1E7.1
AC090498.1
AC004453.8
SCGB3A1
ACPP
HLA−B
CD81
TXNIP
AC007969.5
RP4−706A16.3
MMP7
AP000936.1
GAPDH
P4HB
ATP1A1
FXYD3
SLPI
ITM2B
EEF2
PLA2G2A
RP3−423B22.5
UBC
CTD−3035D6.1
HSPB1
AC012005.1
EEF1A1
S100A6
EZR
OLFM4
PTPRF
LTF
H3F3B
AC018738.2
GSTP1
ACTG1

contributions_gene_type.tsv.gz Factor 16

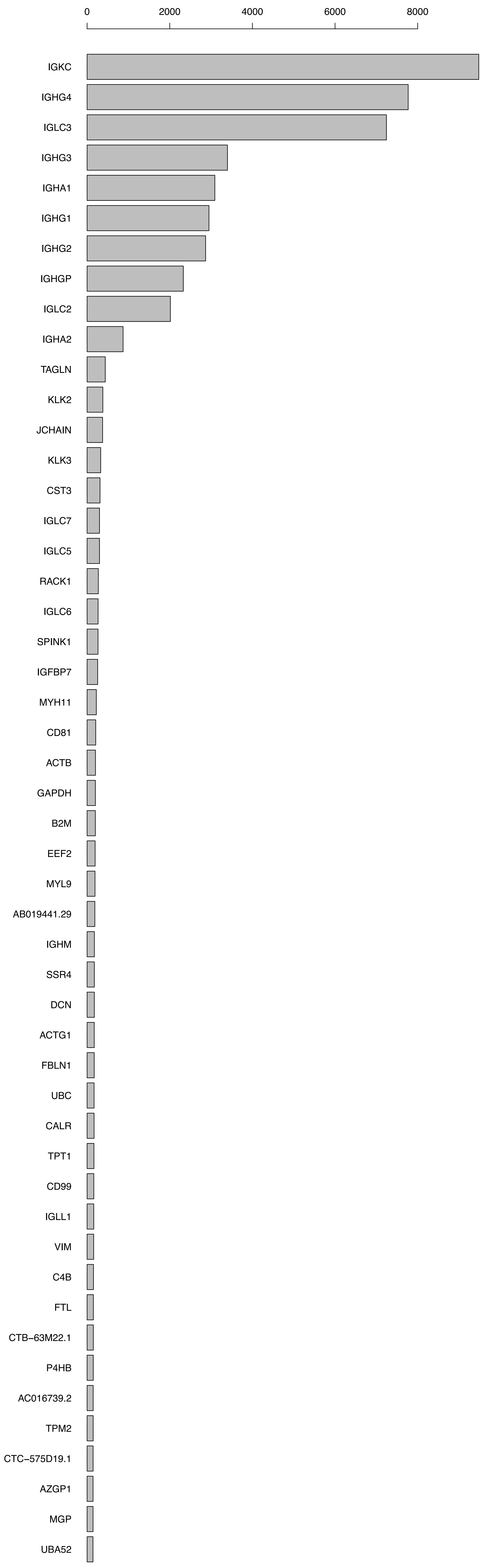

Supplement: Supplementary file 4 — Supplementary Dataset [file 41467_2022_33069_MOESM4_ESM.zip › Supplementary Data Files Nature Communications (Marklund et al. 2022)/Supplementary_Data_File4_STD_Patient2_Genes_16_Factors.pdf]
