# Supplementary material for: Spatio-temporal analysis of prostate tumors in situ suggests pre-existence of treatment-resistant clones
Source: Nat Commun. 2022 Sep 17;13:5475. doi: 10.1038/s41467-022-33069-3 (PMC9482614; doi:10.1038/s41467-022-33069-3)
Supplement: Supplementary file 4 — Supplementary Dataset [file 41467_2022_33069_MOESM4_ESM.zip › Supplementary Data Files Nature Communications (Marklund et al. 2022)/Supplementary_Data_File2_STD_Patient1_Genes_13_Factors.pdf]

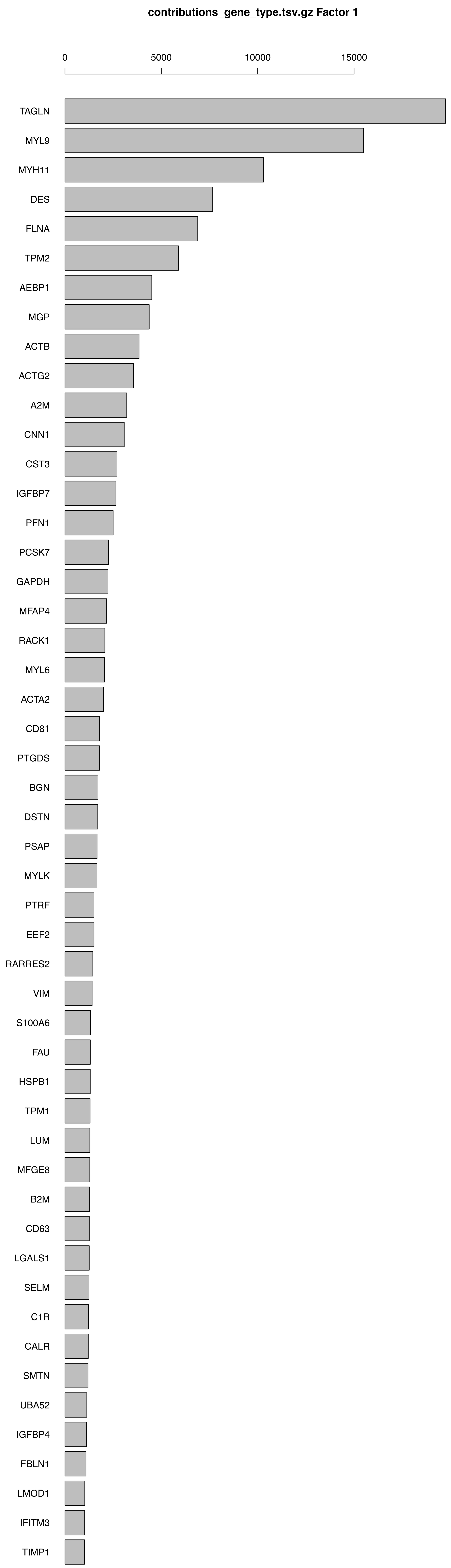
contributions_gene_type.tsv.gz Factor 1
0
5000
10000
15000
TAGLN
MYL9
MYH11
DES
FLNA
TPM2
AEBP1
MGP
ACTB
ACTG2
A2M
CNN1
CST3
IGFBP7
PFN1
PCSK7
GAPDH
MFAP4
RACK1
MYL6
ACTA2
CD81
PTGDS
BGN
DSTN
PSAP
MYLK
PTRF
EEF2
RARRES2
VIM
S100A6
FAU
HSPB1
TPM1
LUM
MFGE8
B2M
CD63
LGALS1
SELM
C1R
CALR
SMTN
UBA52
IGFBP4
FBLN1
LMOD1
IFITM3
TIMP1

contributions_gene_type.tsv.gz Factor 2

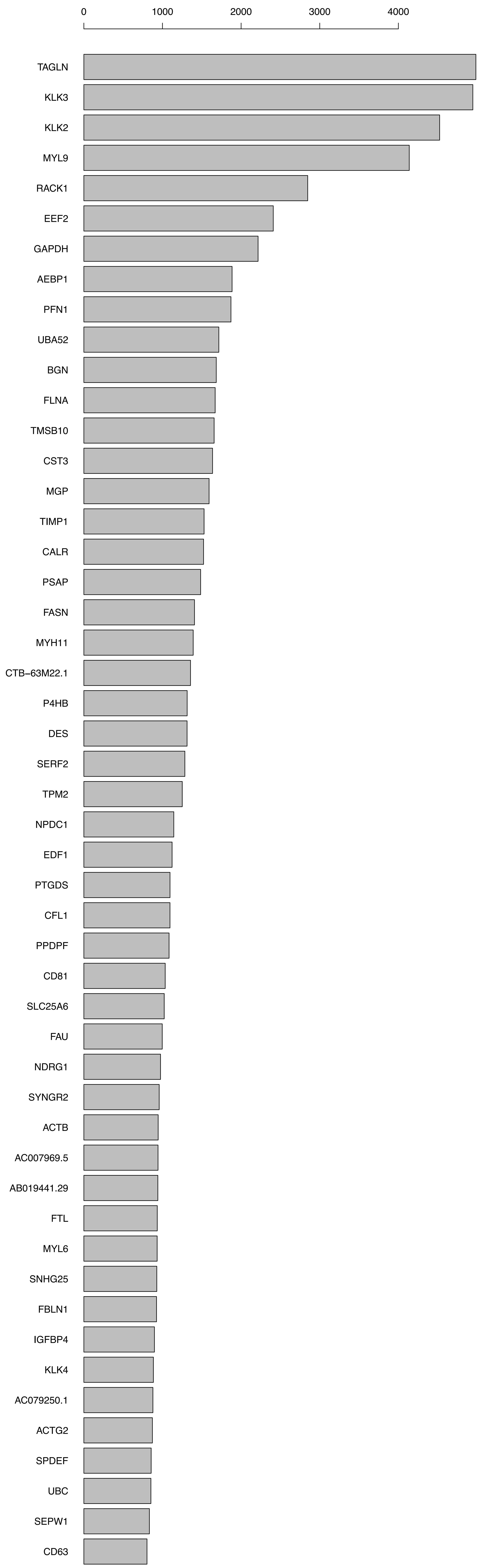

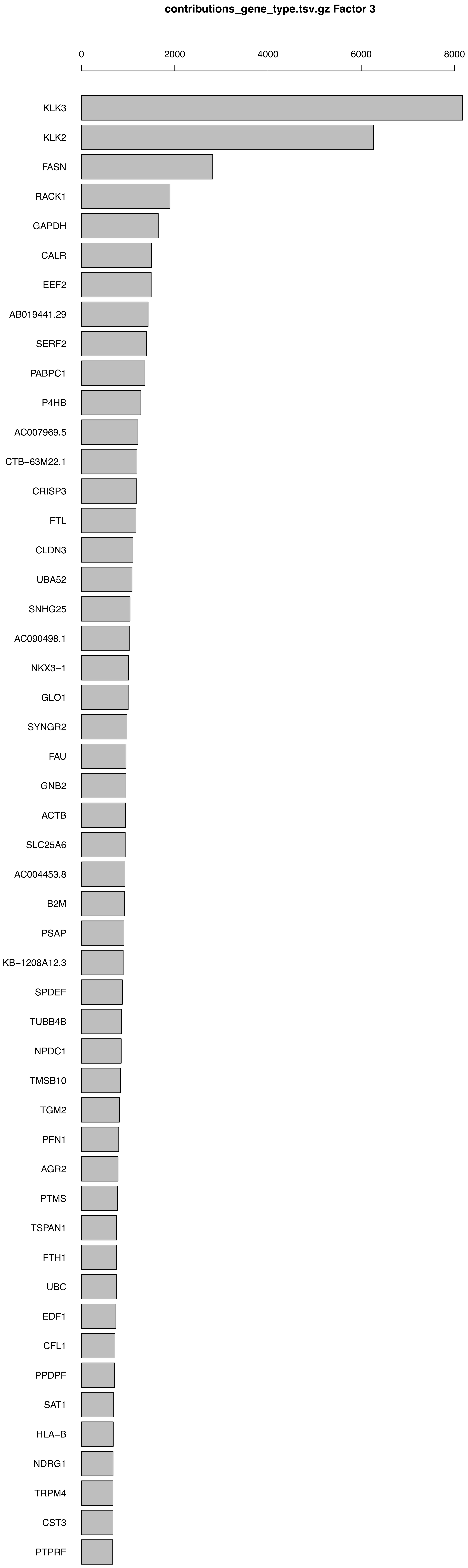
contributions_gene_type.tsv.gz Factor 3
0
2000
4000
6000
8000
KLK3
KLK2
FASN
RACK1
GAPDH
CALR
EEF2
AB019441.29
SERF2
PABPC1
P4HB
AC007969.5
CTB−63M22.1
CRISP3
FTL
CLDN3
UBA52
SNHG25
AC090498.1
NKX3−1
GLO1
SYNGR2
FAU
GNB2
ACTB
SLC25A6
AC004453.8
B2M
PSAP
KB−1208A12.3
SPDEF
TUBB4B
NPDC1
TMSB10
TGM2
PFN1
AGR2
PTMS
TSPAN1
FTH1
UBC
EDF1
CFL1
PPDPF
SAT1
HLA−B
NDRG1
TRPM4
CST3
PTPRF

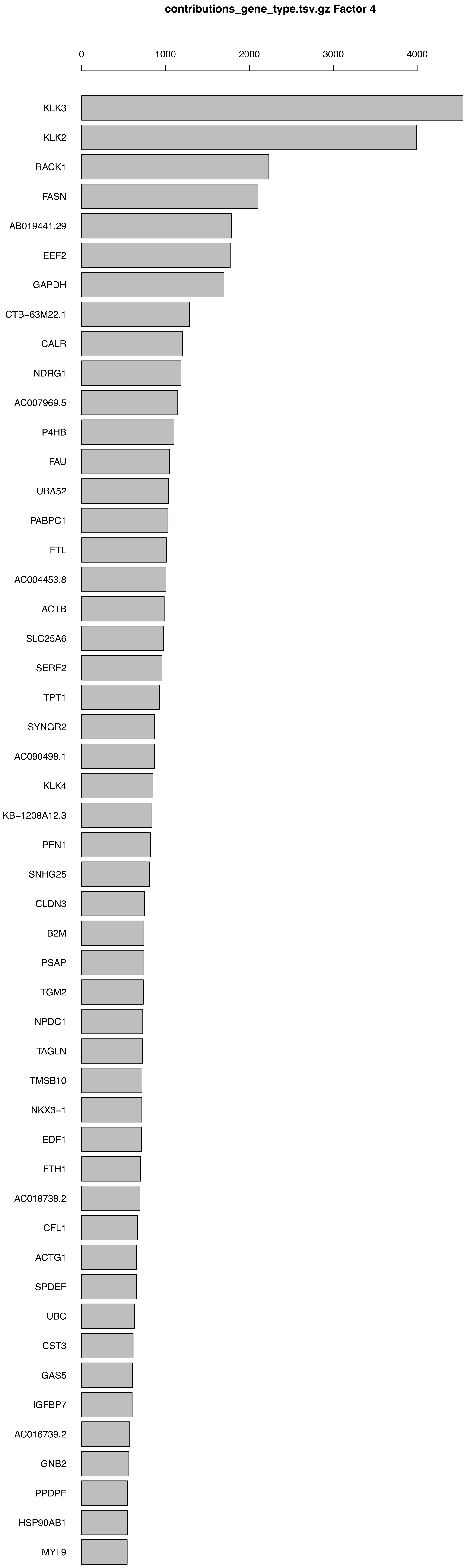
contributions_gene_type.tsv.gz Factor 4
0
1000
2000
3000
4000
KLK3
KLK2
RACK1
FASN
AB019441.29
EEF2
GAPDH
CTB−63M22.1
CALR
NDRG1
AC007969.5
P4HB
FAU
UBA52
PABPC1
FTL
AC004453.8
ACTB
SLC25A6
SERF2
TPT1
SYNGR2
AC090498.1
KLK4
KB−1208A12.3
PFN1
SNHG25
CLDN3
B2M
PSAP
TGM2
NPDC1
TAGLN
TMSB10
NKX3−1
EDF1
FTH1
AC018738.2
CFL1
ACTG1
SPDEF
UBC
CST3
GAS5
IGFBP7
AC016739.2
GNB2
PPDPF
HSP90AB1
MYL9

contributions_gene_type.tsv.gz Factor 5

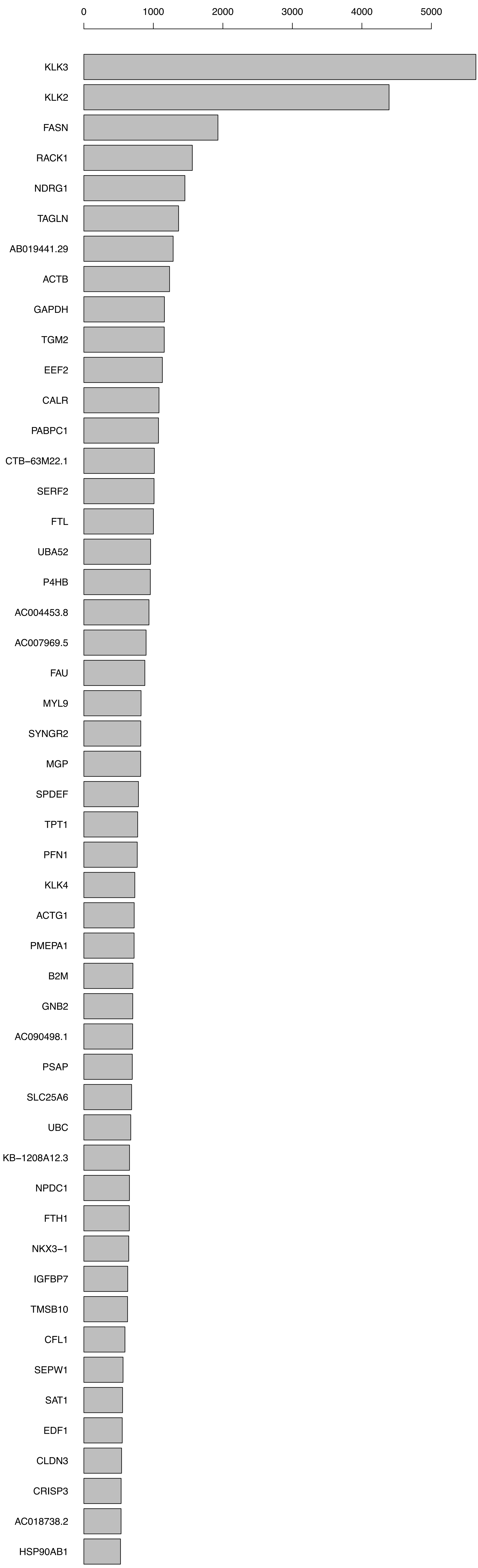

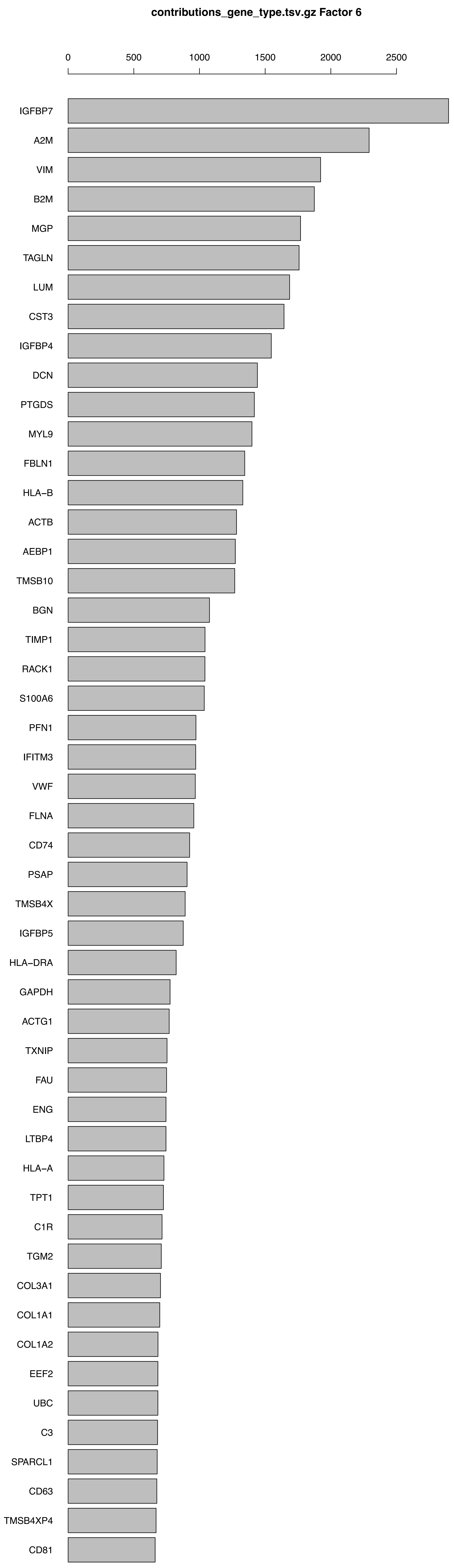
contributions_gene_type.tsv.gz Factor 6
0
500
1000
1500
2000
2500
IGFBP7
A2M
VIM
B2M
MGP
TAGLN
LUM
CST3
IGFBP4
DCN
PTGDS
MYL9
FBLN1
HLA−B
ACTB
AEBP1
TMSB10
BGN
TIMP1
RACK1
S100A6
PFN1
IFITM3
VWF
FLNA
CD74
PSAP
TMSB4X
IGFBP5
HLA−DRA
GAPDH
ACTG1
TXNIP
FAU
ENG
LTBP4
HLA−A
TPT1
C1R
TGM2
COL3A1
COL1A1
COL1A2
EEF2
UBC
C3
SPARCL1
CD63
TMSB4XP4
CD81

contributions_gene_type.tsv.gz Factor 7

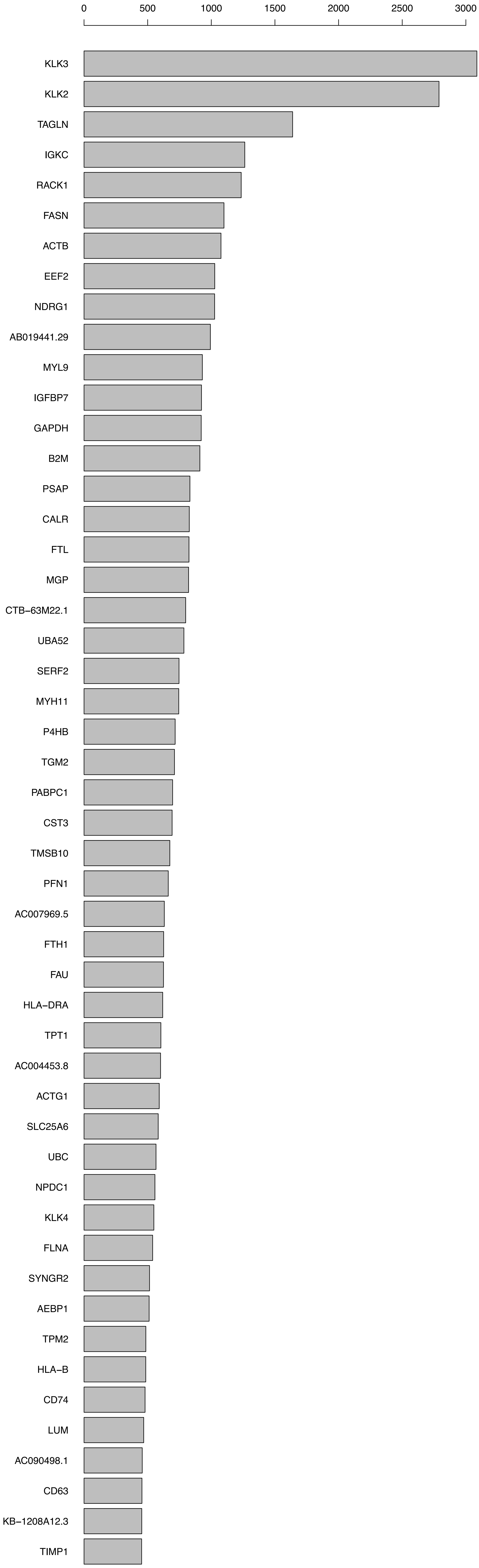

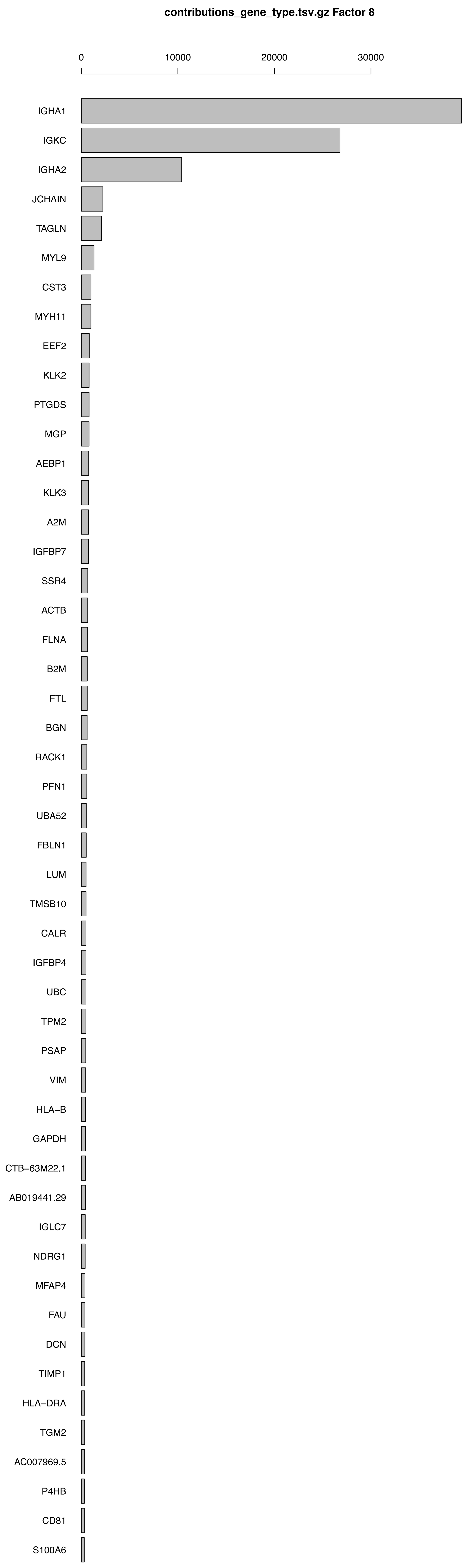
contributions_gene_type.tsv.gz Factor 8
0
10000
20000
30000
IGHA1
IGKC
IGHA2
JCHAIN
TAGLN
MYL9
CST3
MYH11
EEF2
KLK2
PTGDS
MGP
AEBP1
KLK3
A2M
IGFBP7
SSR4
ACTB
FLNA
B2M
FTL
BGN
RACK1
PFN1
UBA52
FBLN1
LUM
TMSB10
CALR
IGFBP4
UBC
TPM2
PSAP
VIM
HLA−B
GAPDH
CTB−63M22.1
AB019441.29
IGLC7
NDRG1
MFAP4
FAU
DCN
TIMP1
HLA−DRA
TGM2
AC007969.5
P4HB
CD81
S100A6

contributions_gene_type.tsv.gz Factor 9

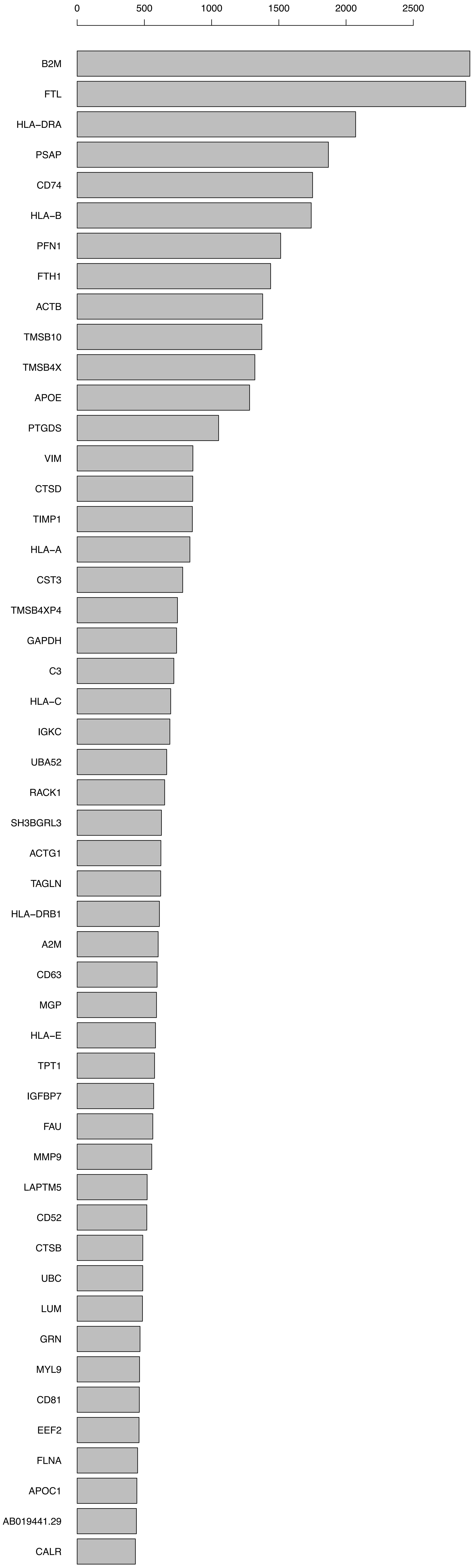

contributions_gene_type.tsv.gz Factor 10

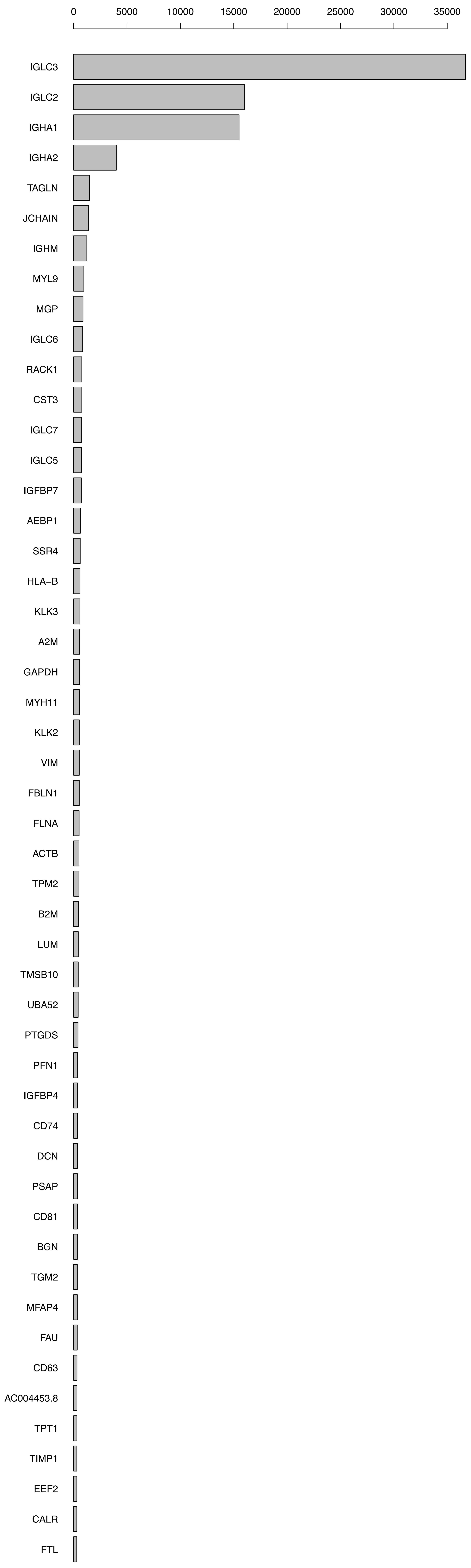

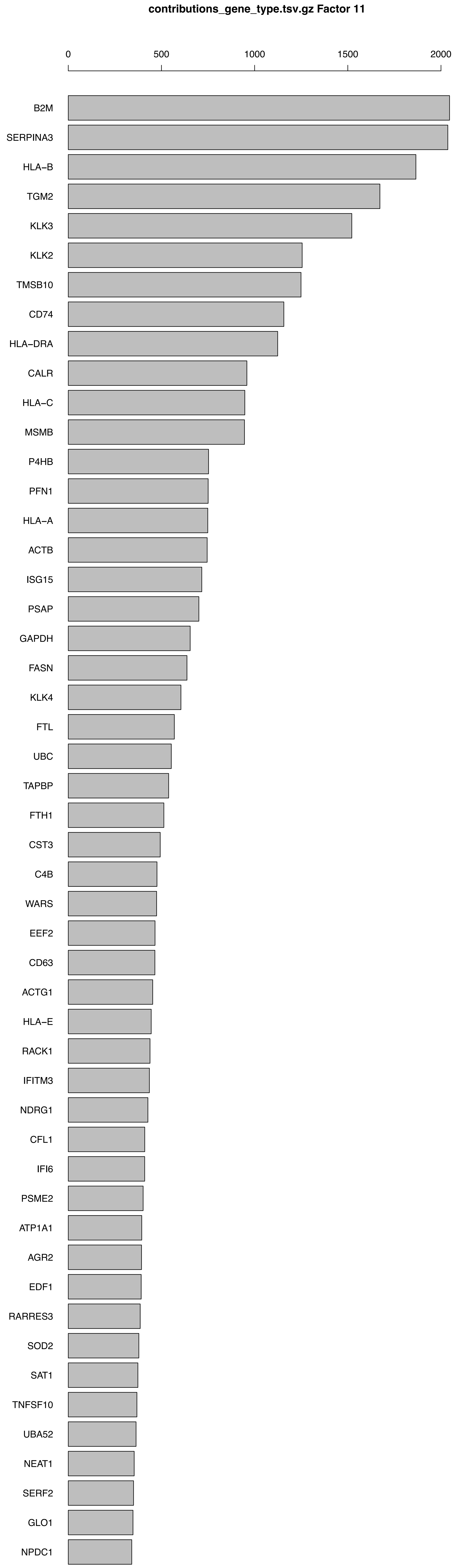
contributions_gene_type.tsv.gz Factor 11
0
500
1000
1500
2000
B2M
SERPINA3
HLA−B
TGM2
KLK3
KLK2
TMSB10
CD74
HLA−DRA
CALR
HLA−C
MSMB
P4HB
PFN1
HLA−A
ACTB
ISG15
PSAP
GAPDH
FASN
KLK4
FTL
UBC
TAPBP
FTH1
CST3
C4B
WARS
EEF2
CD63
ACTG1
HLA−E
RACK1
IFITM3
NDRG1
CFL1
IFI6
PSME2
ATP1A1
AGR2
EDF1
RARRES3
SOD2
SAT1
TNFSF10
UBA52
NEAT1
SERF2
GLO1
NPDC1

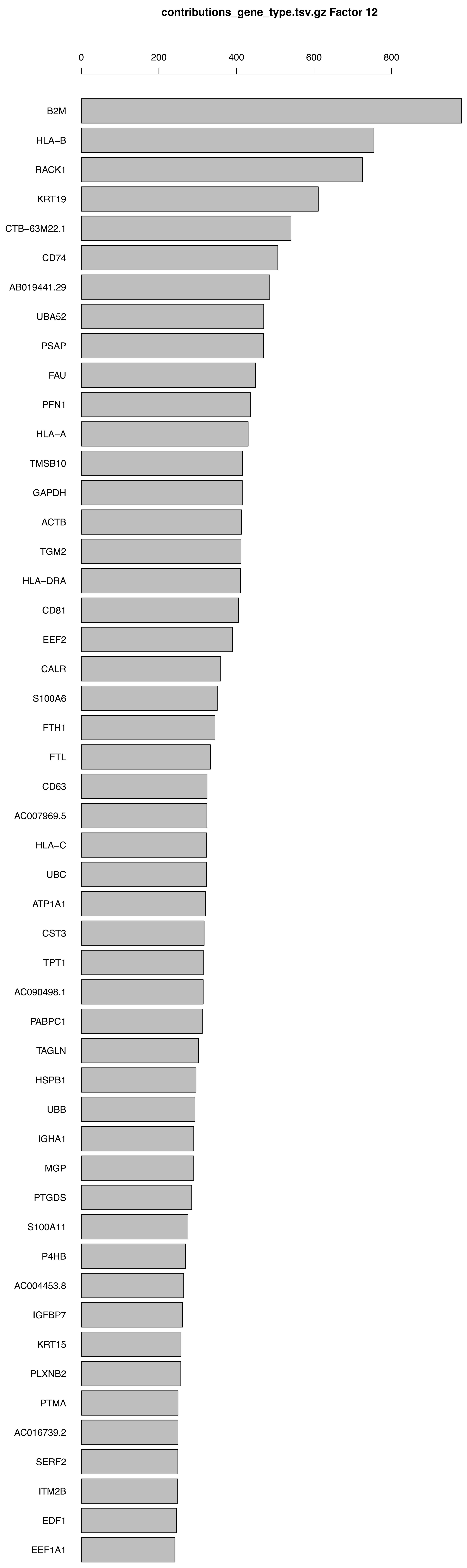
contributions_gene_type.tsv.gz Factor 12
0
200
400
600
800
B2M
HLA−B
RACK1
KRT19
CTB−63M22.1
CD74
AB019441.29
UBA52
PSAP
FAU
PFN1
HLA−A
TMSB10
GAPDH
ACTB
TGM2
HLA−DRA
CD81
EEF2
CALR
S100A6
FTH1
FTL
CD63
AC007969.5
HLA−C
UBC
ATP1A1
CST3
TPT1
AC090498.1
PABPC1
TAGLN
HSPB1
UBB
IGHA1
MGP
PTGDS
S100A11
P4HB
AC004453.8
IGFBP7
KRT15
PLXNB2
PTMA
AC016739.2
SERF2
ITM2B
EDF1
EEF1A1

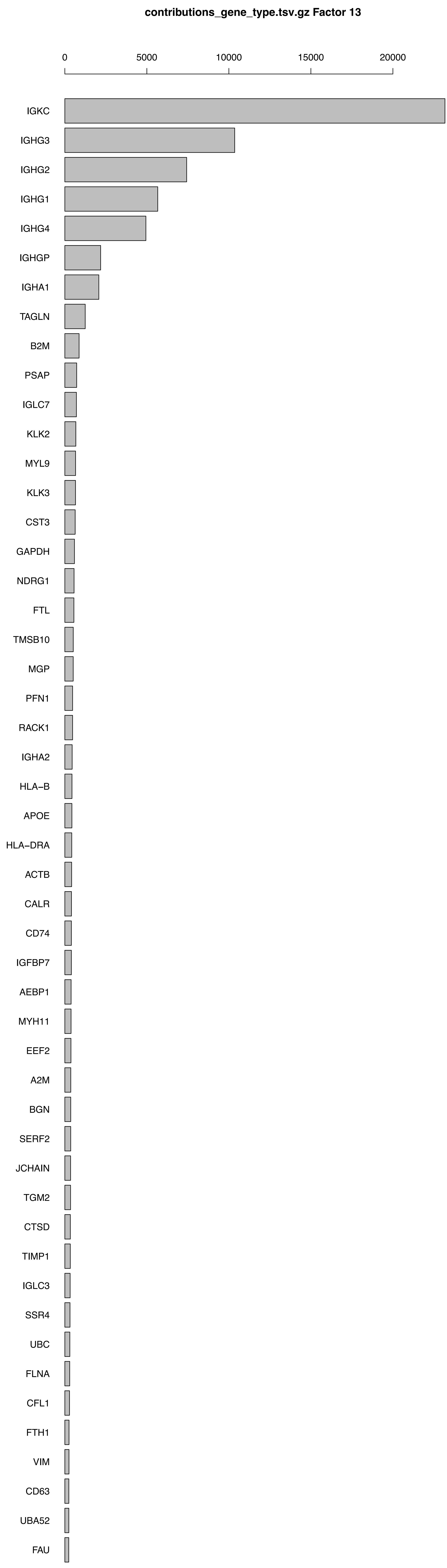
contributions_gene_type.tsv.gz Factor 13
0
5000
10000
15000
20000
IGKC
IGHG3
IGHG2
IGHG1
IGHG4
IGHGP
IGHA1
TAGLN
B2M
PSAP
IGLC7
KLK2
MYL9
KLK3
CST3
GAPDH
NDRG1
FTL
TMSB10
MGP
PFN1
RACK1
IGHA2
HLA−B
APOE
HLA−DRA
ACTB
CALR
CD74
IGFBP7
AEBP1
MYH11
EEF2
A2M
BGN
SERF2
JCHAIN
TGM2
CTSD
TIMP1
IGLC3
SSR4
UBC
FLNA
CFL1
FTH1
VIM
CD63
UBA52
FAU
