# Supplementary material for: Spatio-temporal analysis of prostate tumors in situ suggests pre-existence of treatment-resistant clones
Source: Nat Commun. 2022 Sep 17;13:5475. doi: 10.1038/s41467-022-33069-3 (PMC9482614; doi:10.1038/s41467-022-33069-3)
Supplement: Supplementary file 4 — Supplementary Dataset [file 41467_2022_33069_MOESM4_ESM.zip › Supplementary Data Files Nature Communications (Marklund et al. 2022)/Supplementary_Data_File6_STD_Patient3_Genes_10_Factors.pdf]

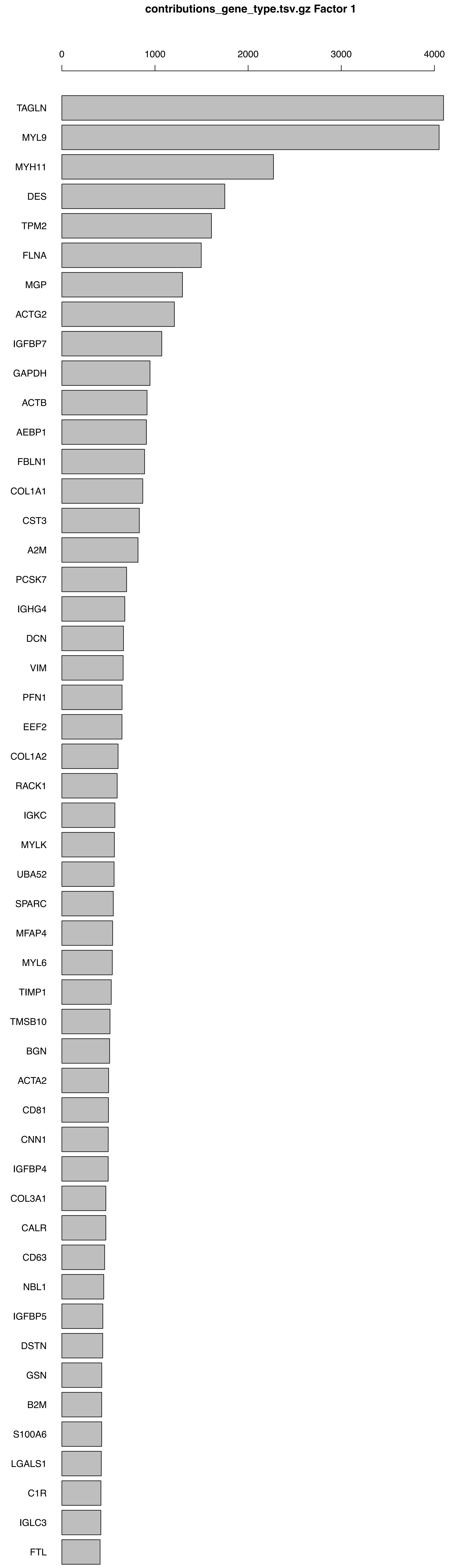
contributions_gene_type.tsv.gz Factor 1
0
1000
2000
3000
4000
TAGLN
MYL9
MYH11
DES
TPM2
FLNA
MGP
ACTG2
IGFBP7
GAPDH
ACTB
AEBP1
FBLN1
COL1A1
CST3
A2M
PCSK7
IGHG4
DCN
VIM
PFN1
EEF2
COL1A2
RACK1
IGKC
MYLK
UBA52
SPARC
MFAP4
MYL6
TIMP1
TMSB10
BGN
ACTA2
CD81
CNN1
IGFBP4
COL3A1
CALR
CD63
NBL1
IGFBP5
DSTN
GSN
B2M
S100A6
LGALS1
C1R
IGLC3
FTL

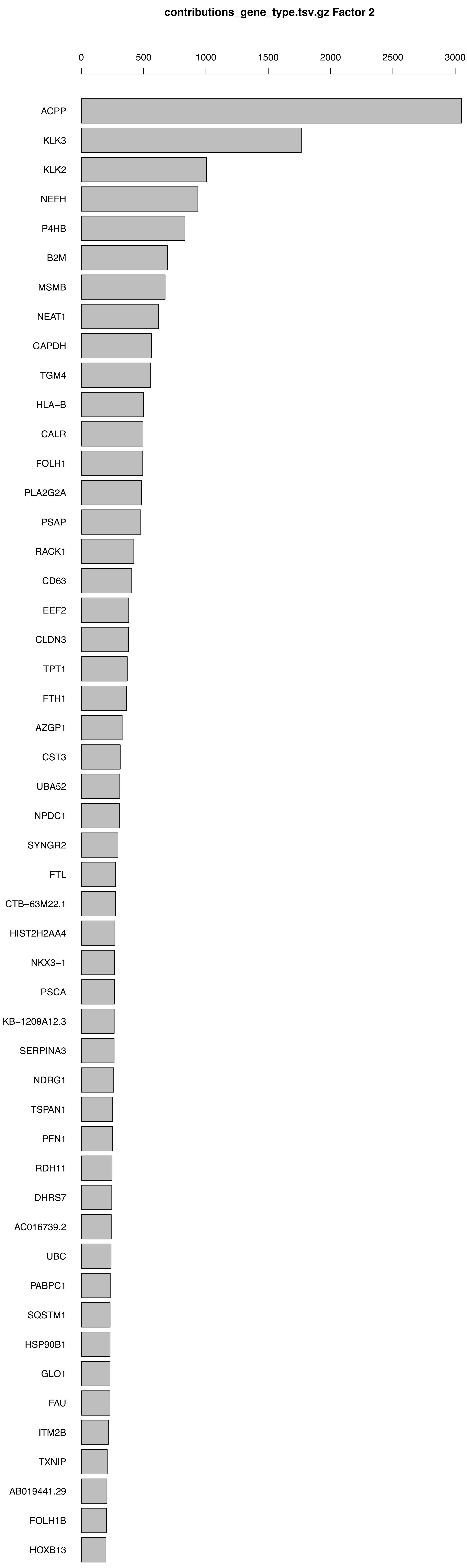
contributions_gene_type.tsv.gz Factor 2
0
500
1000
1500
2000
2500
3000
ACPP
KLK3
KLK2
NEFH
P4HB
B2M
MSMB
NEAT1
GAPDH
TGM4
HLA−B
CALR
FOLH1
PLA2G2A
PSAP
RACK1
CD63
EEF2
CLDN3
TPT1
FTH1
AZGP1
CST3
UBA52
NPDC1
SYNGR2
FTL
CTB−63M22.1
HIST2H2AA4
NKX3−1
PSCA
KB−1208A12.3
SERPINA3
NDRG1
TSPAN1
PFN1
RDH11
DHRS7
AC016739.2
UBC
PABPC1
SQSTM1
HSP90B1
GLO1
FAU
ITM2B
TXNIP
AB019441.29
FOLH1B
HOXB13

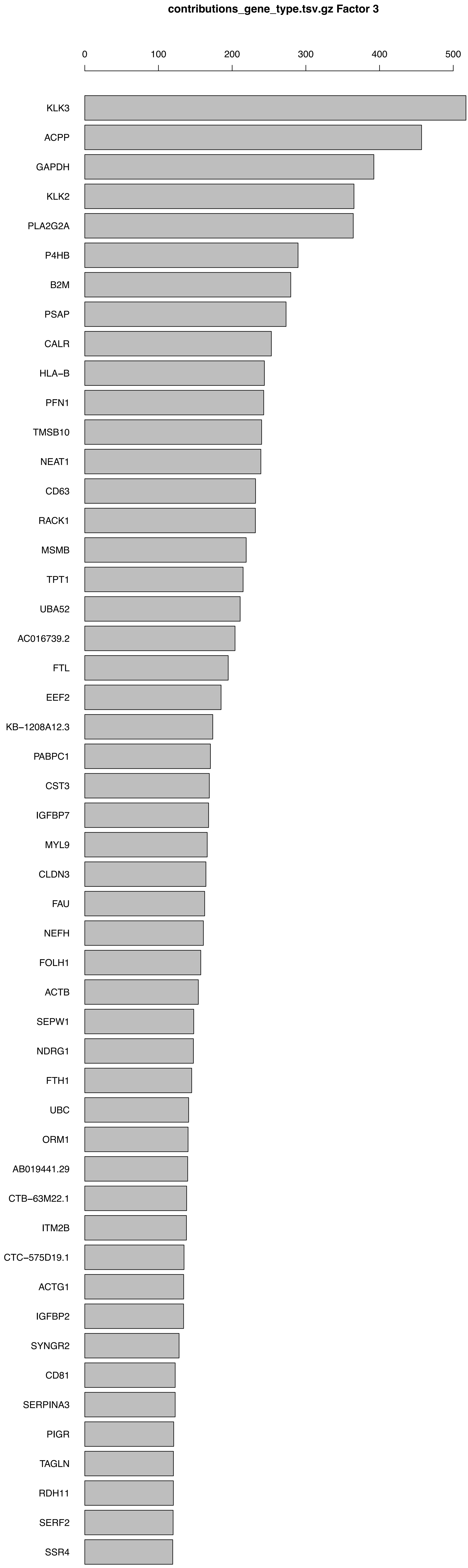
contributions_gene_type.tsv.gz Factor 3
0
100
200
300
400
500
KLK3
ACPP
GAPDH
KLK2
PLA2G2A
P4HB
B2M
PSAP
CALR
HLA−B
PFN1
TMSB10
NEAT1
CD63
RACK1
MSMB
TPT1
UBA52
AC016739.2
FTL
EEF2
KB−1208A12.3
PABPC1
CST3
IGFBP7
MYL9
CLDN3
FAU
NEFH
FOLH1
ACTB
SEPW1
NDRG1
FTH1
UBC
ORM1
AB019441.29
CTB−63M22.1
ITM2B
CTC−575D19.1
ACTG1
IGFBP2
SYNGR2
CD81
SERPINA3
PIGR
TAGLN
RDH11
SERF2
SSR4

contributions_gene_type.tsv.gz Factor 4

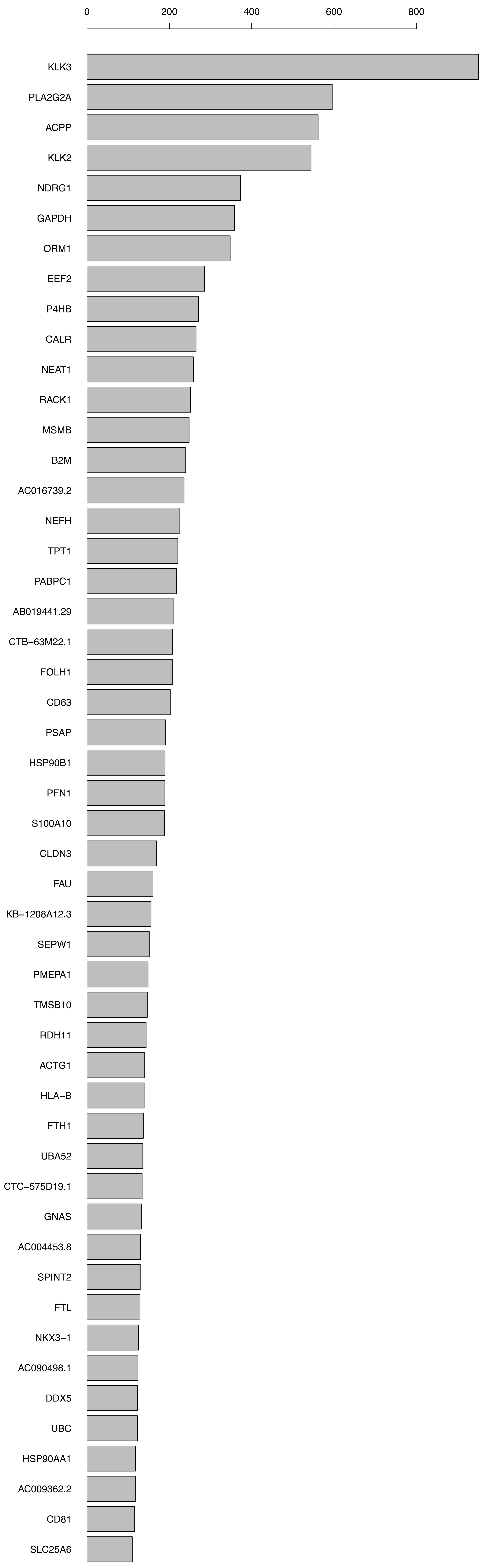

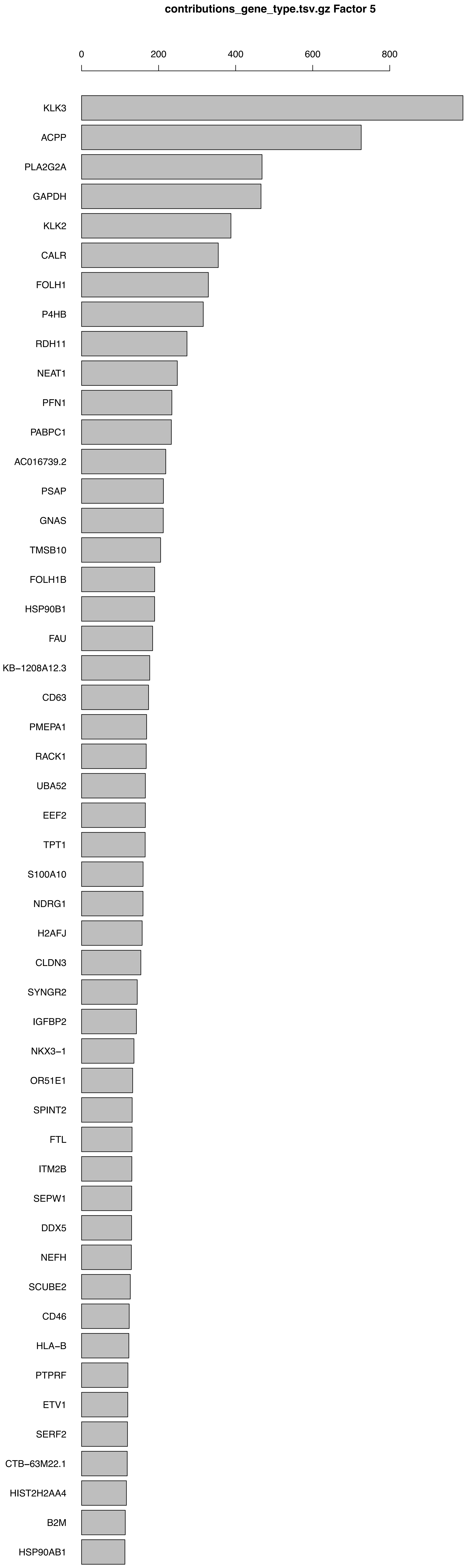
contributions_gene_type.tsv.gz Factor 5
0
200
400
600
800
KLK3
ACPP
PLA2G2A
GAPDH
KLK2
CALR
FOLH1
P4HB
RDH11
NEAT1
PFN1
PABPC1
AC016739.2
PSAP
GNAS
TMSB10
FOLH1B
HSP90B1
FAU
KB−1208A12.3
CD63
PMEPA1
RACK1
UBA52
EEF2
TPT1
S100A10
NDRG1
H2AFJ
CLDN3
SYNGR2
IGFBP2
NKX3−1
OR51E1
SPINT2
FTL
ITM2B
SEPW1
DDX5
NEFH
SCUBE2
CD46
HLA−B
PTPRF
ETV1
SERF2
CTB−63M22.1
HIST2H2AA4
B2M
HSP90AB1

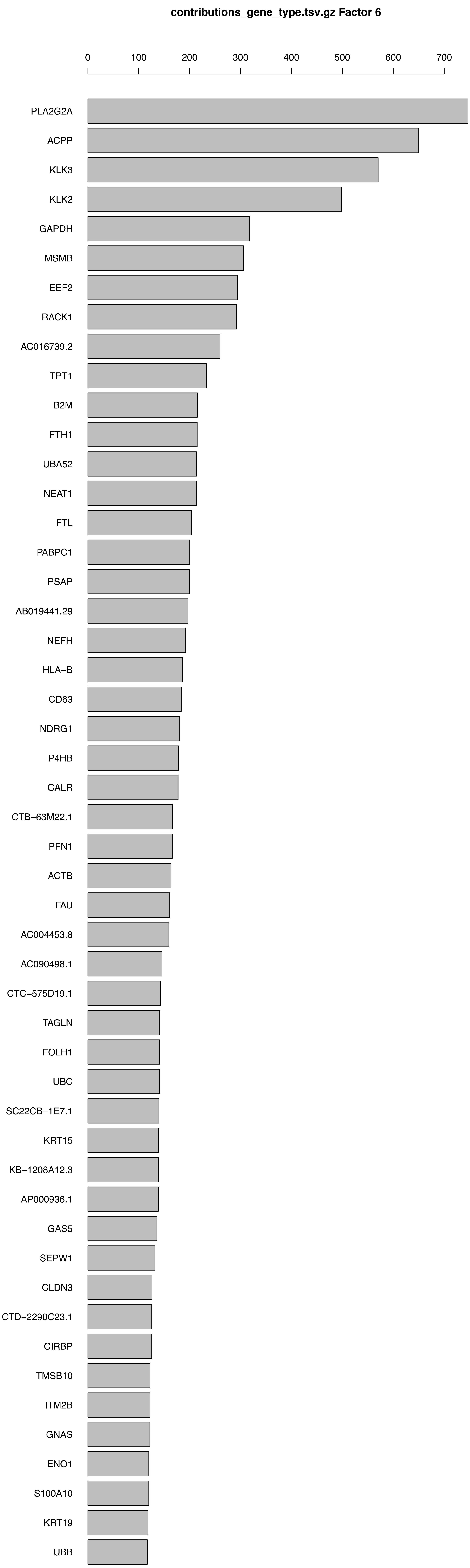
contributions_gene_type.tsv.gz Factor 6
0
100
200
300
400
500
600
700
PLA2G2A
ACPP
KLK3
KLK2
GAPDH
MSMB
EEF2
RACK1
AC016739.2
TPT1
B2M
FTH1
UBA52
NEAT1
FTL
PABPC1
PSAP
AB019441.29
NEFH
HLA−B
CD63
NDRG1
P4HB
CALR
CTB−63M22.1
PFN1
ACTB
FAU
AC004453.8
AC090498.1
CTC−575D19.1
TAGLN
FOLH1
UBC
SC22CB−1E7.1
KRT15
KB−1208A12.3
AP000936.1
GAS5
SEPW1
CLDN3
CTD−2290C23.1
CIRBP
TMSB10
ITM2B
GNAS
ENO1
S100A10
KRT19
UBB

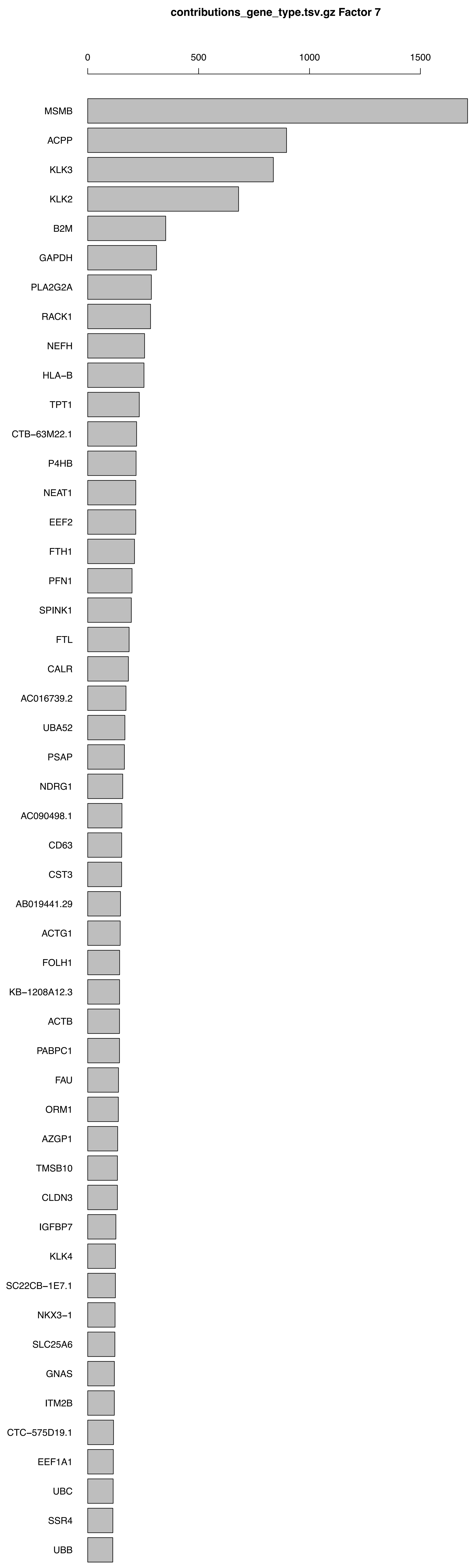
contributions_gene_type.tsv.gz Factor 7
0
500
1000
1500
MSMB
ACPP
KLK3
KLK2
B2M
GAPDH
PLA2G2A
RACK1
NEFH
HLA−B
TPT1
CTB−63M22.1
P4HB
NEAT1
EEF2
FTH1
PFN1
SPINK1
FTL
CALR
AC016739.2
UBA52
PSAP
NDRG1
AC090498.1
CD63
CST3
AB019441.29
ACTG1
FOLH1
KB−1208A12.3
ACTB
PABPC1
FAU
ORM1
AZGP1
TMSB10
CLDN3
IGFBP7
KLK4
SC22CB−1E7.1
NKX3−1
SLC25A6
GNAS
ITM2B
CTC−575D19.1
EEF1A1
UBC
SSR4
UBB

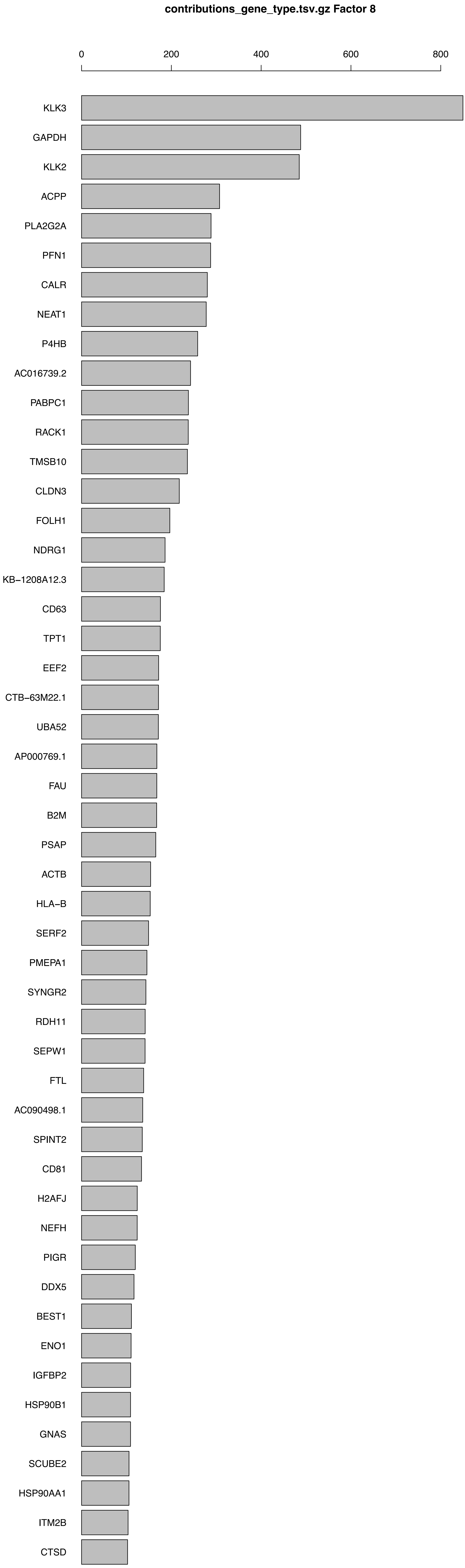

contributions_gene_type.tsv.gz Factor 8
0
200
400
600
800
KLK3
GAPDH
KLK2
ACPP
PLA2G2A
PFN1
CALR
NEAT1
P4HB
AC016739.2
PABPC1
RACK1
TMSB10
CLDN3
FOLH1
NDRG1
KB−1208A12.3
CD63
TPT1
EEF2
CTB−63M22.1
UBA52
AP000769.1
FAU
B2M
PSAP
ACTB
HLA−B
SERF2
PMEPA1
SYNGR2
RDH11
SEPW1
FTL
AC090498.1
SPINT2
CD81
H2AFJ
NEFH
PIGR
DDX5
BEST1
ENO1
IGFBP2
HSP90B1
GNAS
SCUBE2
HSP90AA1
ITM2B
CTSD

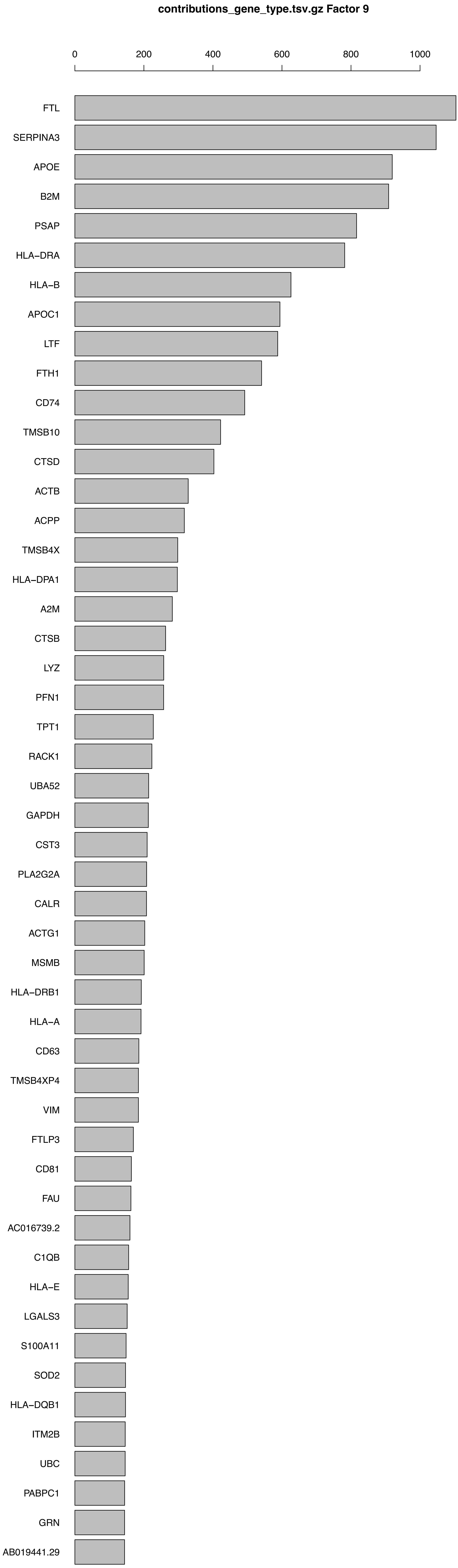
contributions_gene_type.tsv.gz Factor 9
0
200
400
600
800
1000
FTL
SERPINA3
APOE
B2M
PSAP
HLA−DRA
HLA−B
APOC1
LTF
FTH1
CD74
TMSB10
CTSD
ACTB
ACPP
TMSB4X
HLA−DPA1
A2M
CTSB
LYZ
PFN1
TPT1
RACK1
UBA52
GAPDH
CST3
PLA2G2A
CALR
ACTG1
MSMB
HLA−DRB1
HLA−A
CD63
TMSB4XP4
VIM
FTLP3
CD81
FAU
AC016739.2
C1QB
HLA−E
LGALS3
S100A11
SOD2
HLA−DQB1
ITM2B
UBC
PABPC1
GRN
AB019441.29

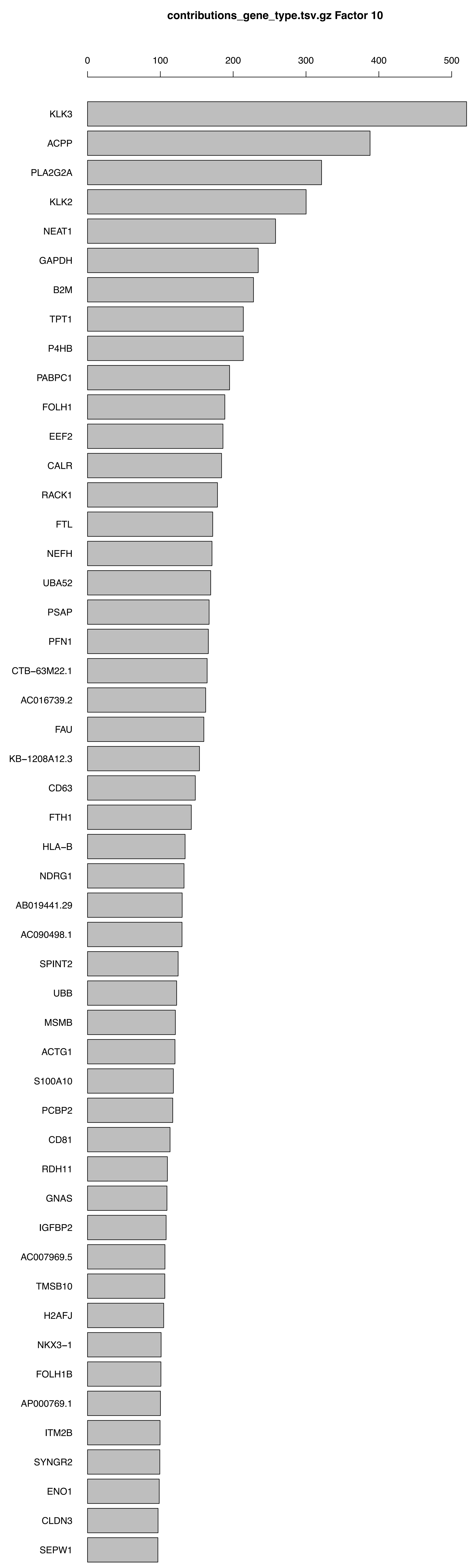
contributions_gene_type.tsv.gz Factor 10
0
100
200
300
400
500
KLK3
ACPP
PLA2G2A
KLK2
NEAT1
GAPDH
B2M
TPT1
P4HB
PABPC1
FOLH1
EEF2
CALR
RACK1
FTL
NEFH
UBA52
PSAP
PFN1
CTB−63M22.1
AC016739.2
FAU
KB−1208A12.3
CD63
FTH1
HLA−B
NDRG1
AB019441.29
AC090498.1
SPINT2
UBB
MSMB
ACTG1
S100A10
PCBP2
CD81
RDH11
GNAS
IGFBP2
AC007969.5
TMSB10
H2AFJ
NKX3−1
FOLH1B
AP000769.1
ITM2B
SYNGR2
ENO1
CLDN3
SEPW1
