# Supplementary figures and images for: Spatio-temporal analysis of prostate tumors in situ suggests pre-existence of treatment-resistant clones
Source: Nat Commun. 2022 Sep 17;13:5475. doi: 10.1038/s41467-022-33069-3 (PMC9482614; doi:10.1038/s41467-022-33069-3)

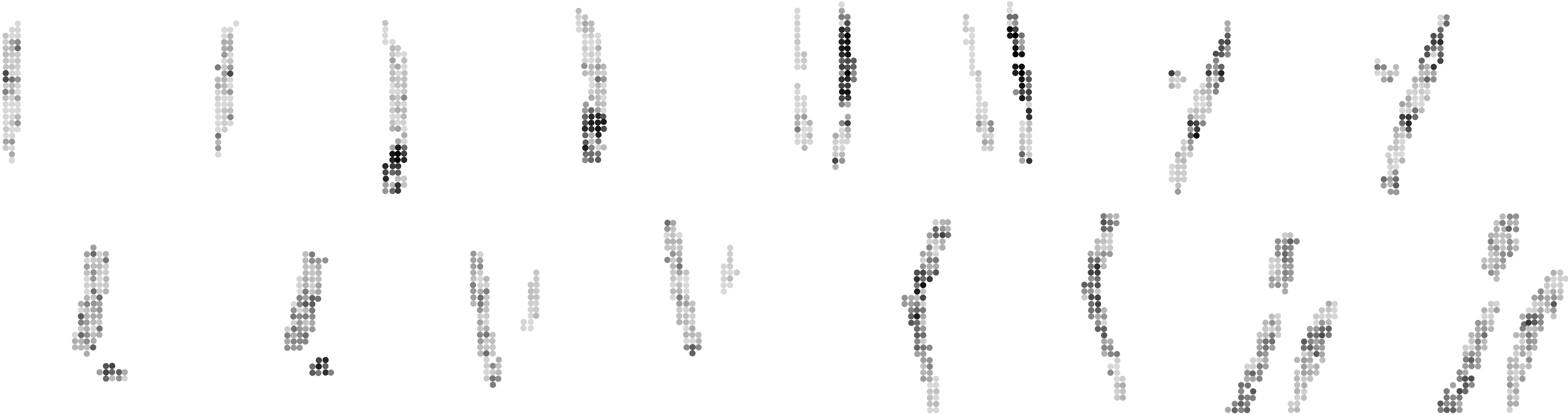

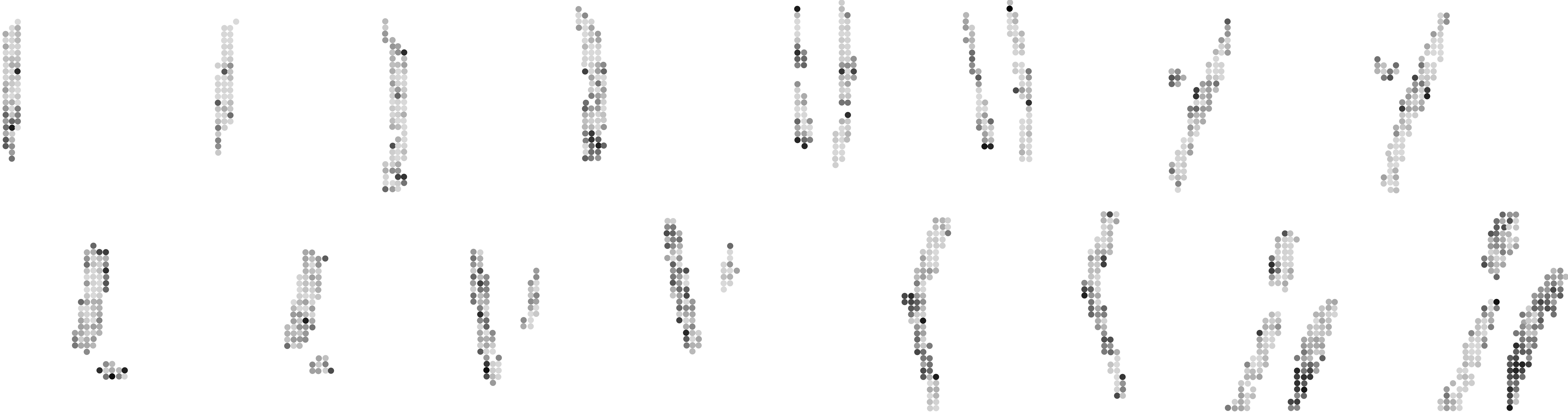

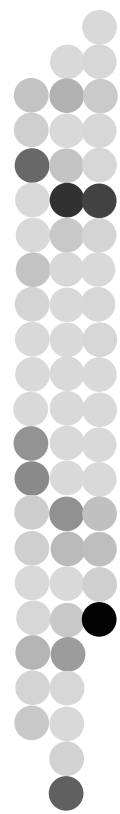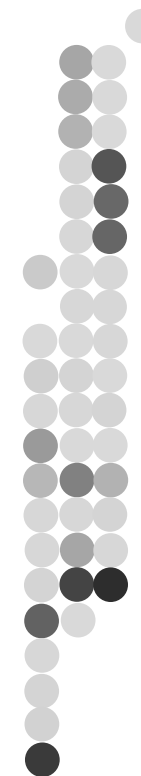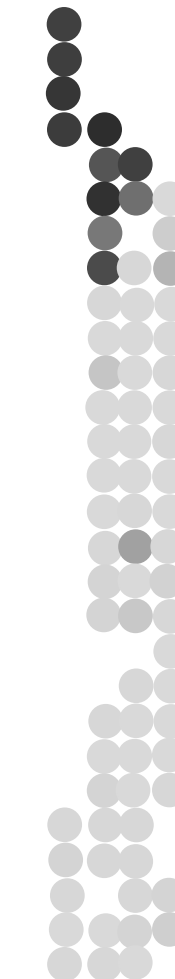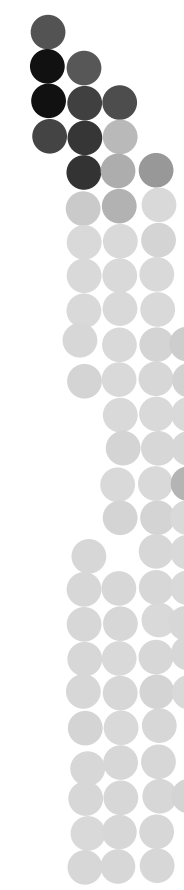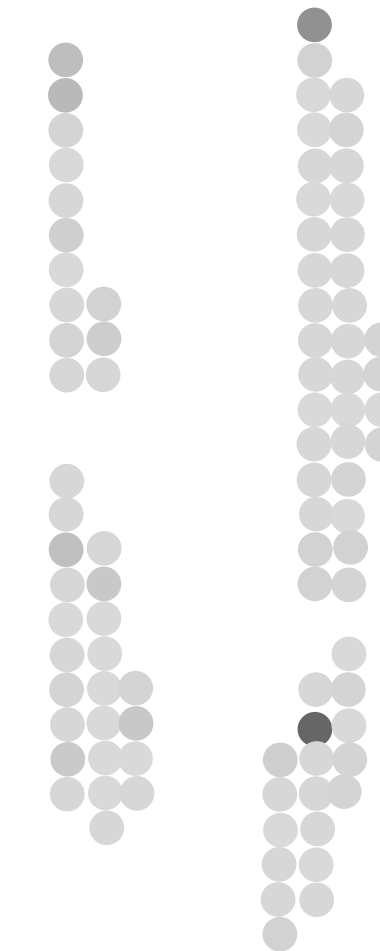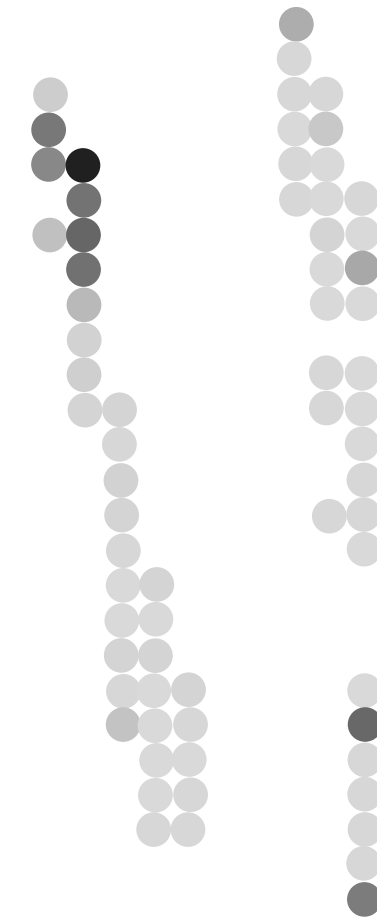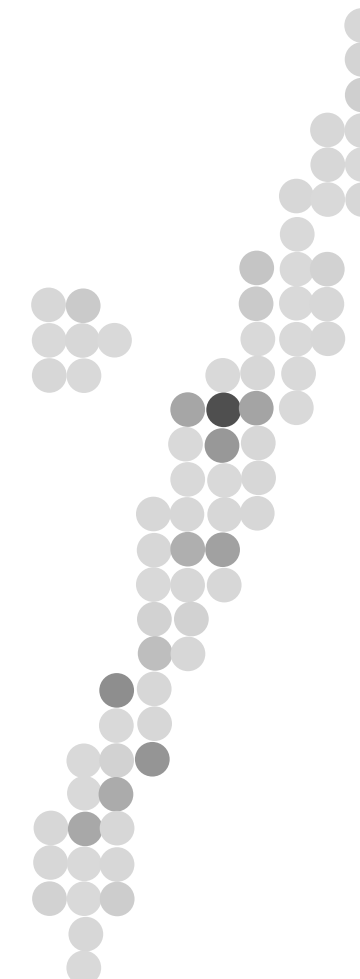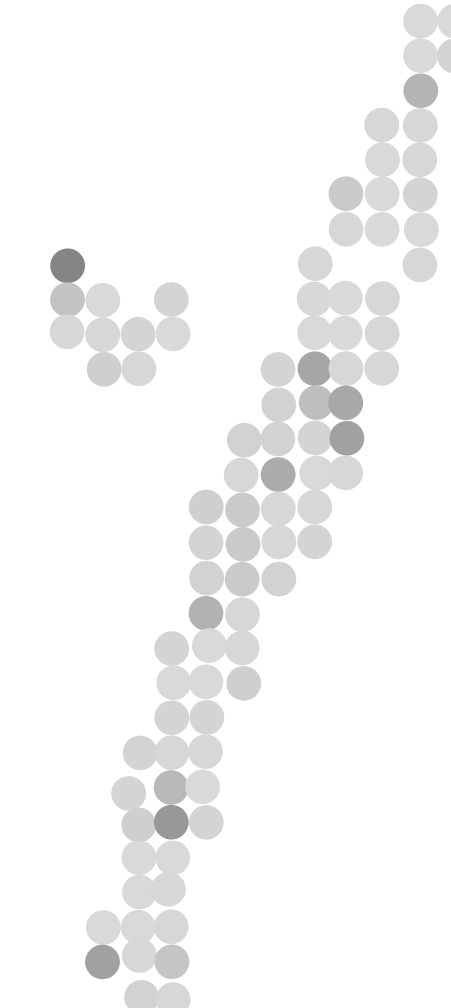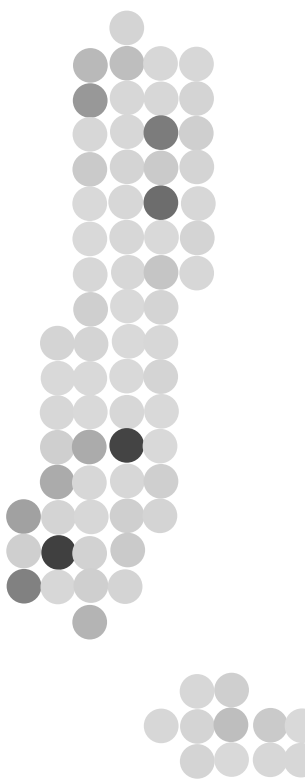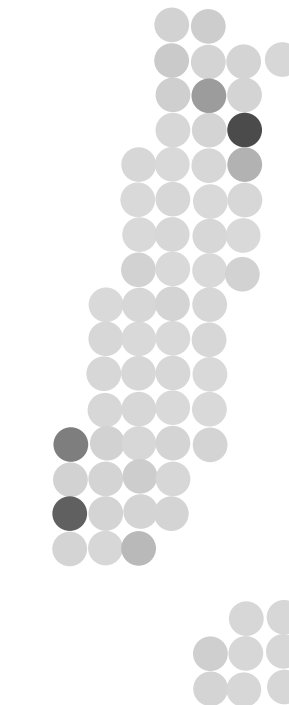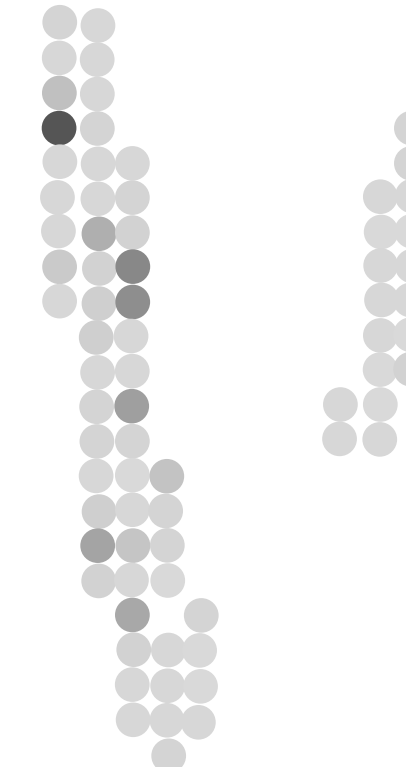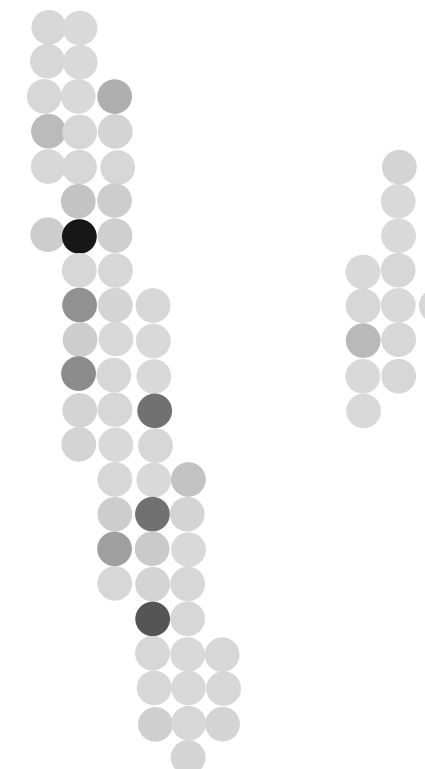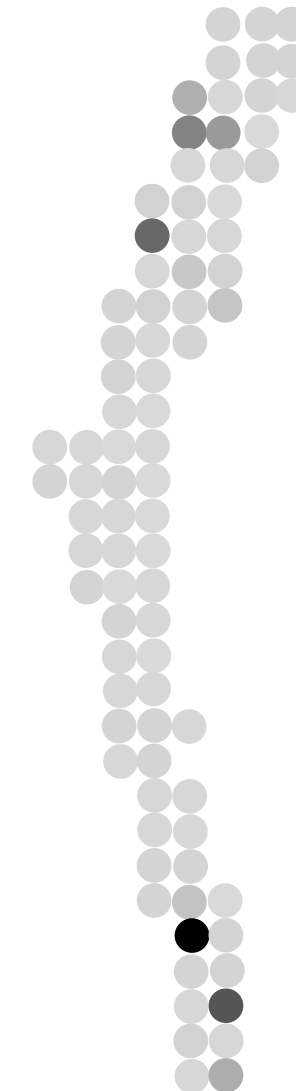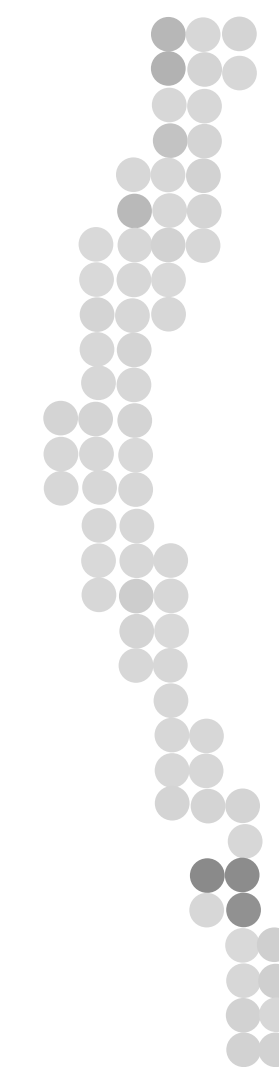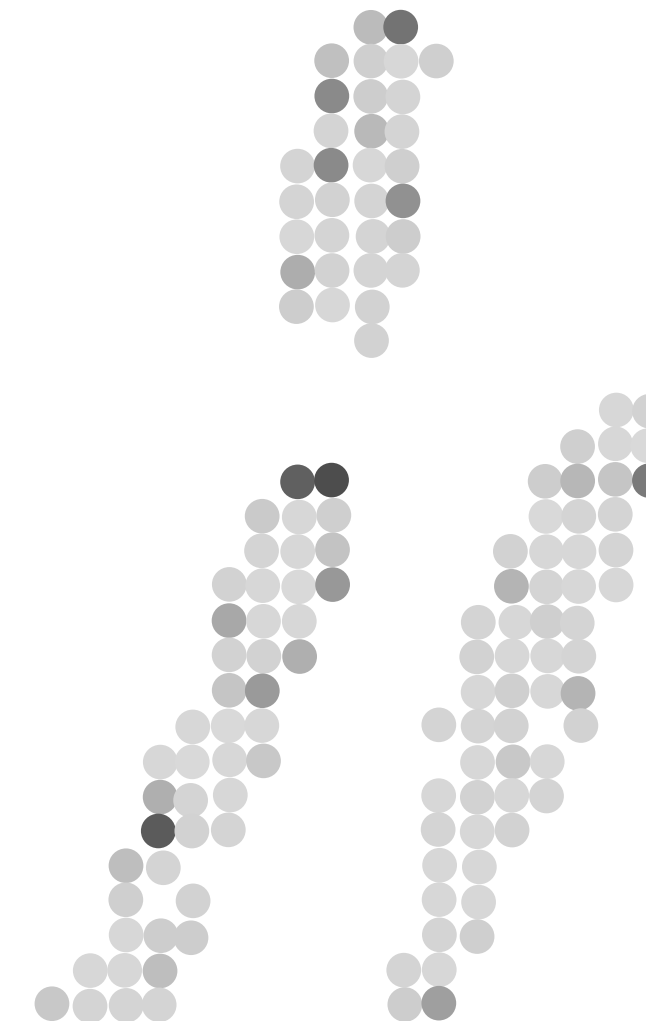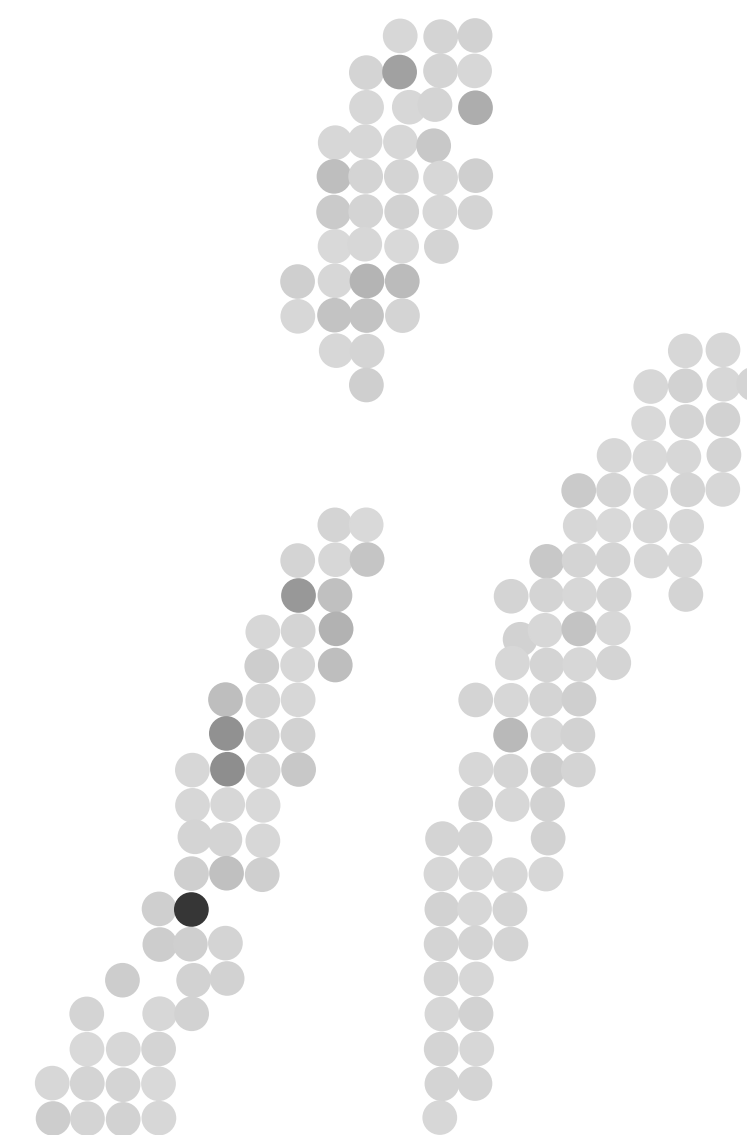

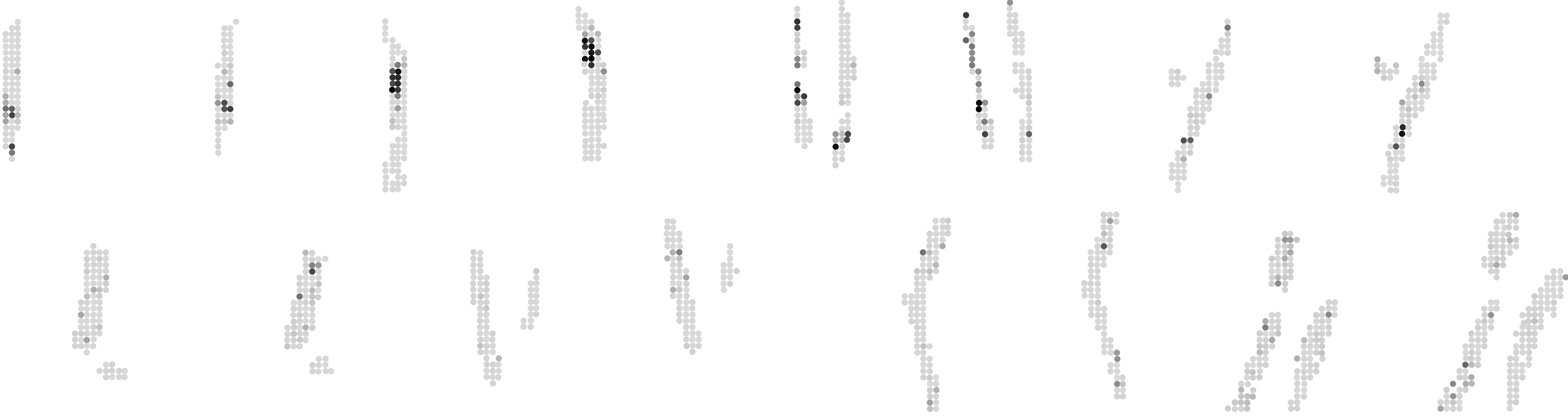

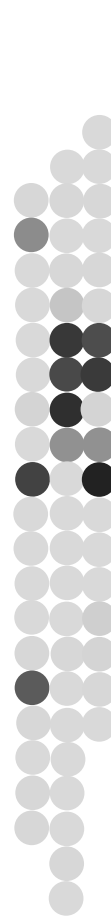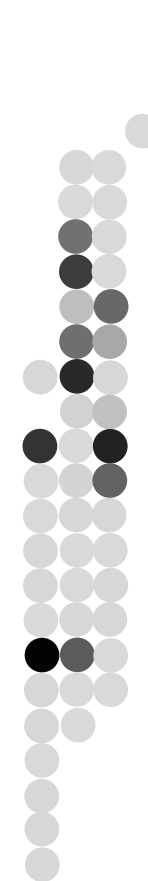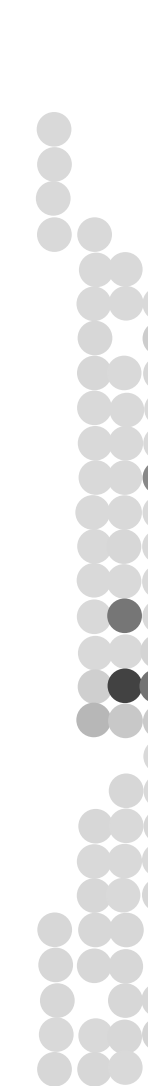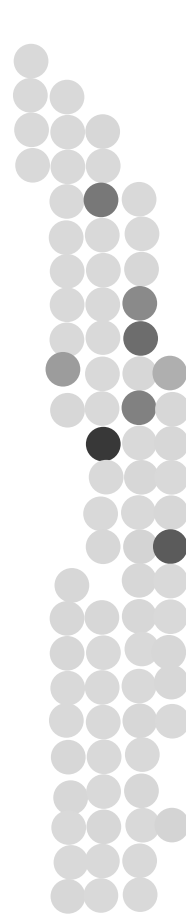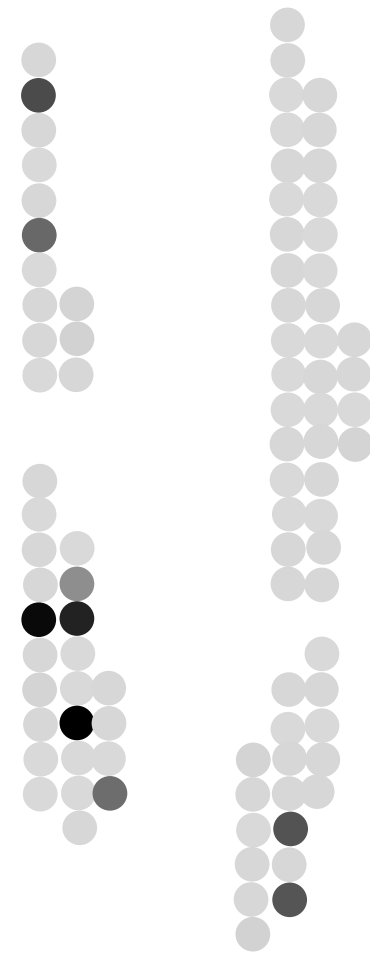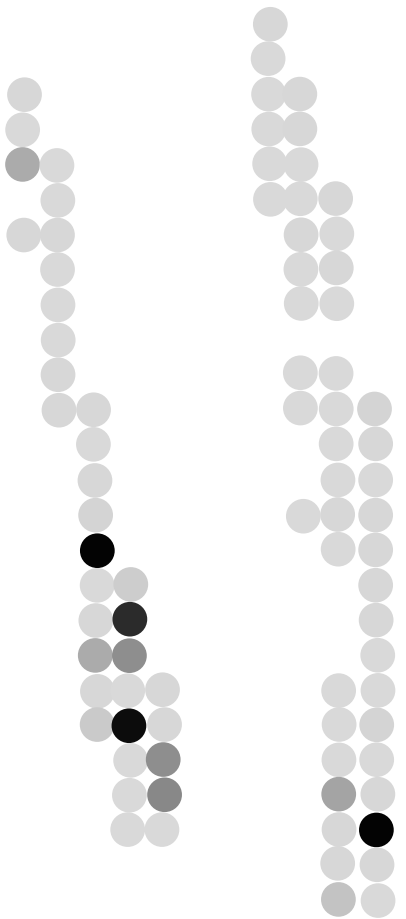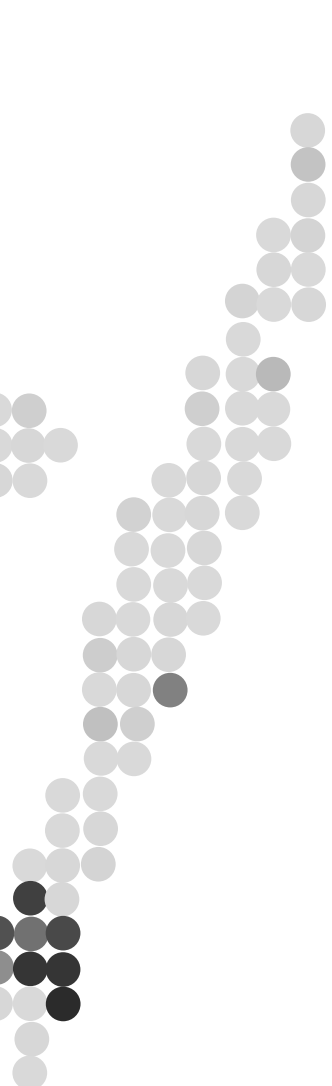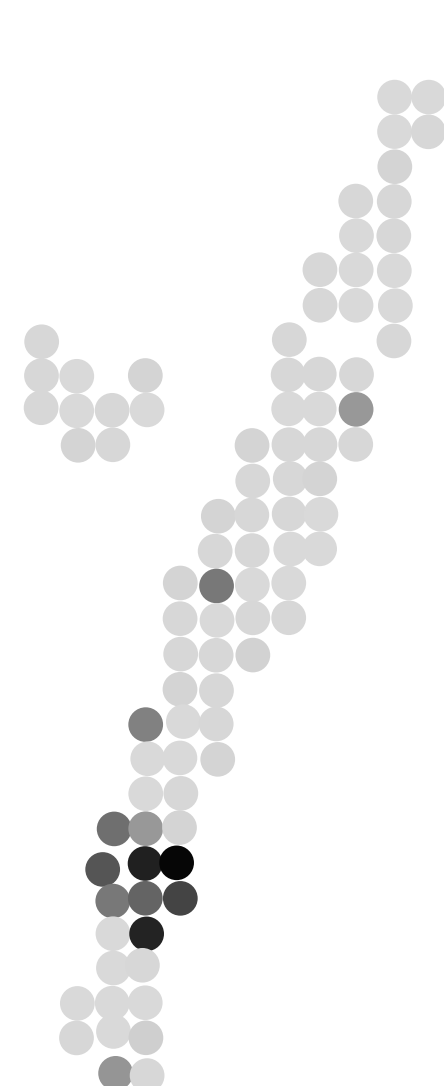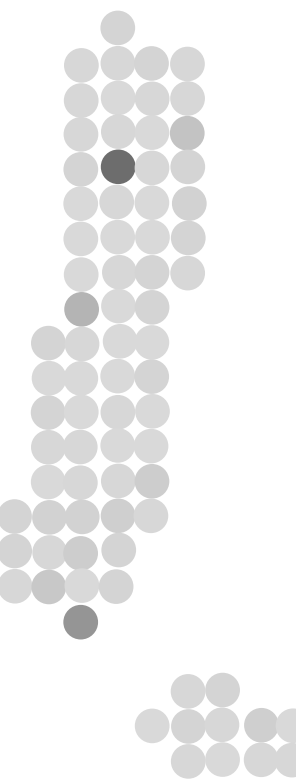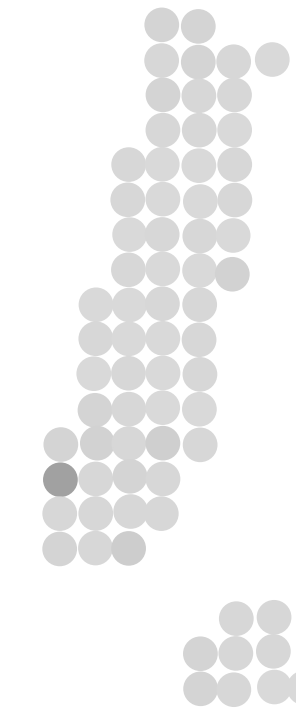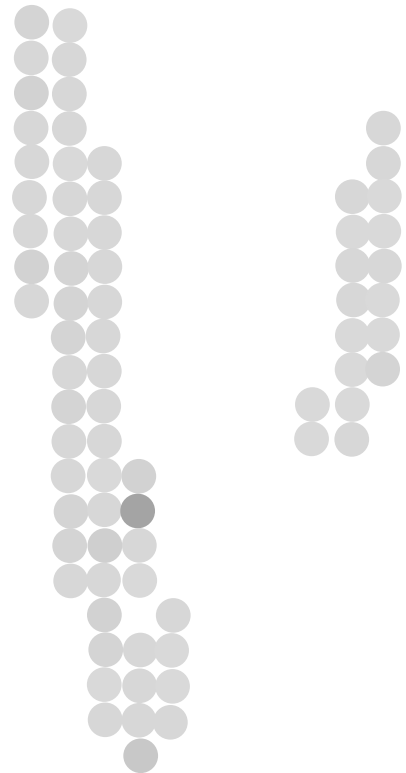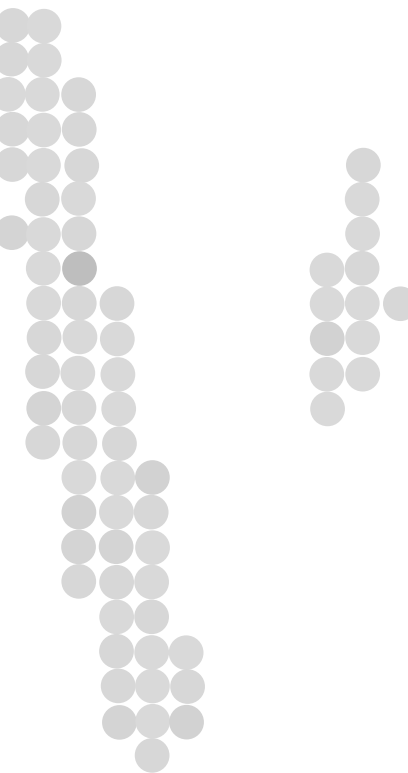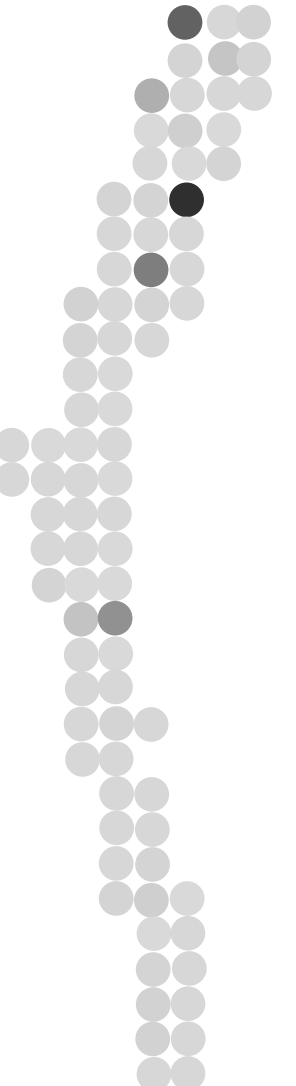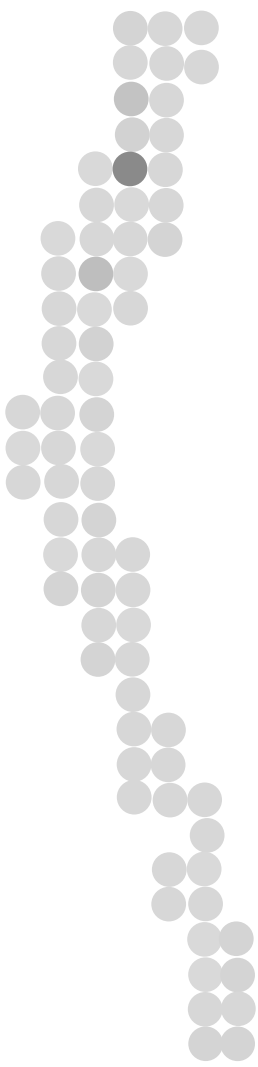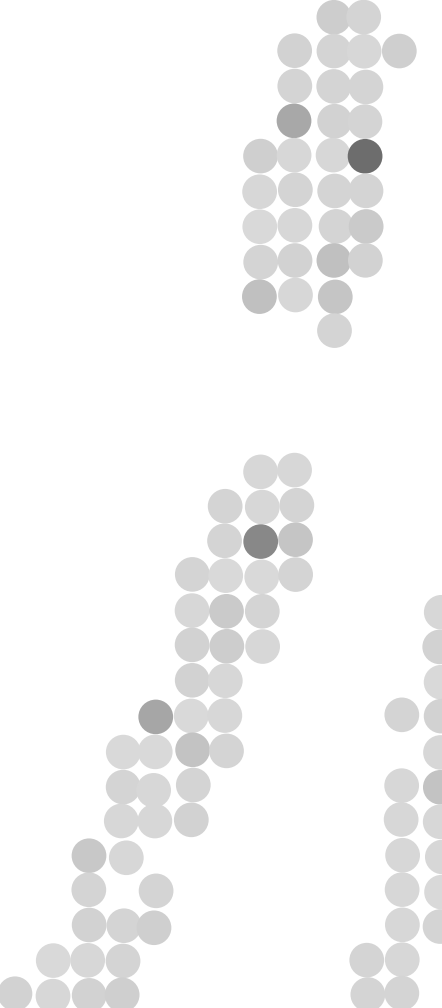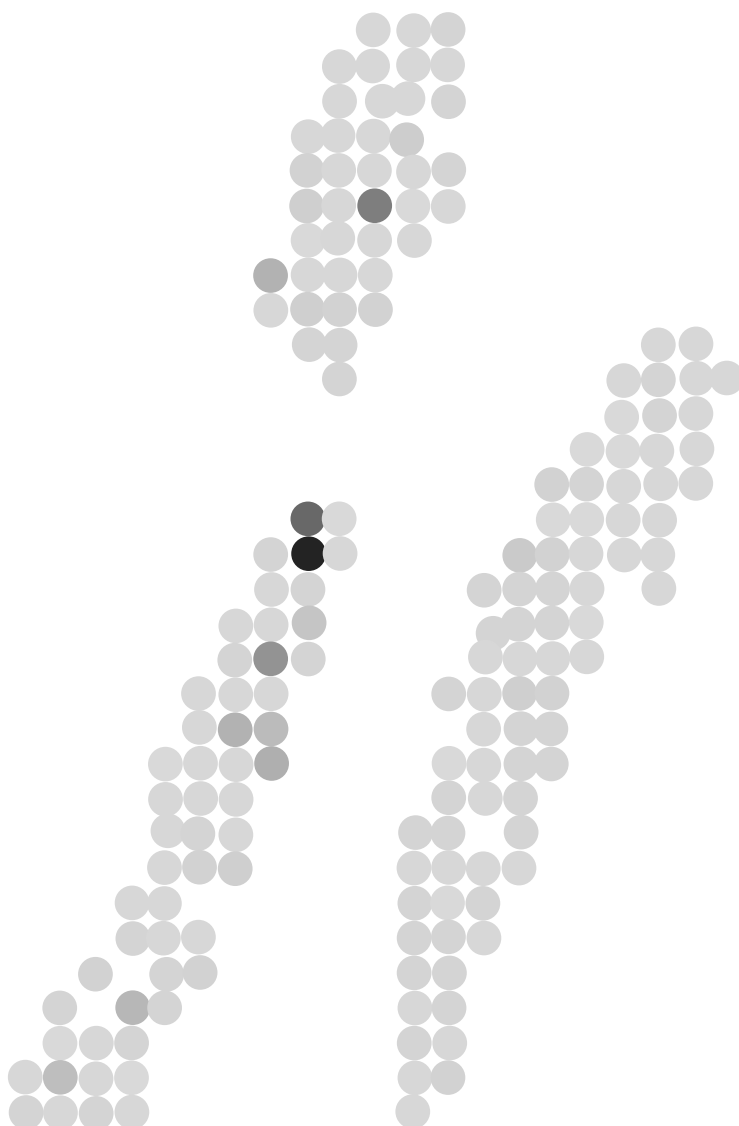

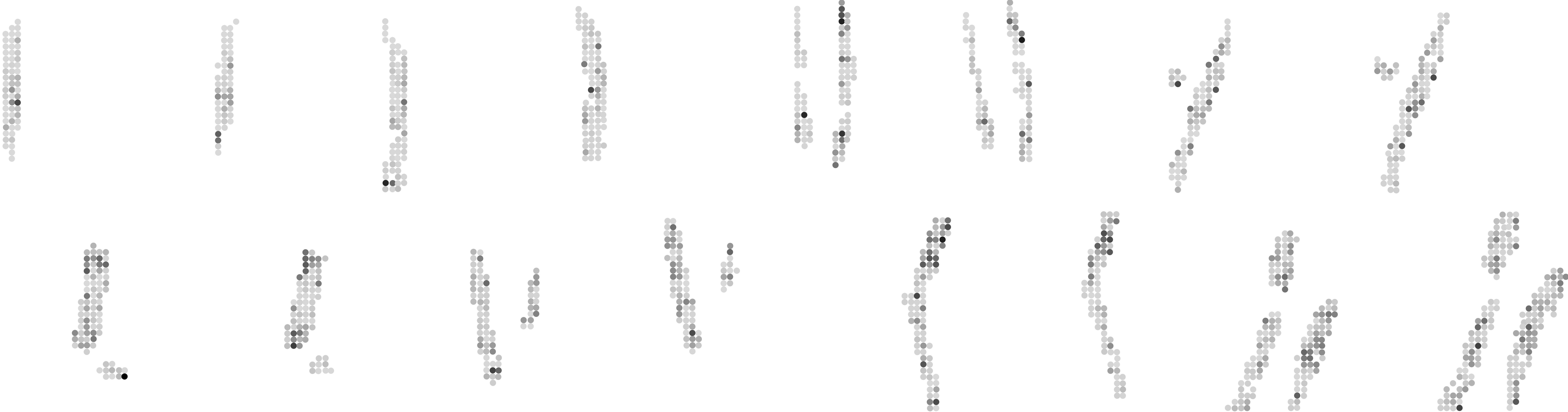

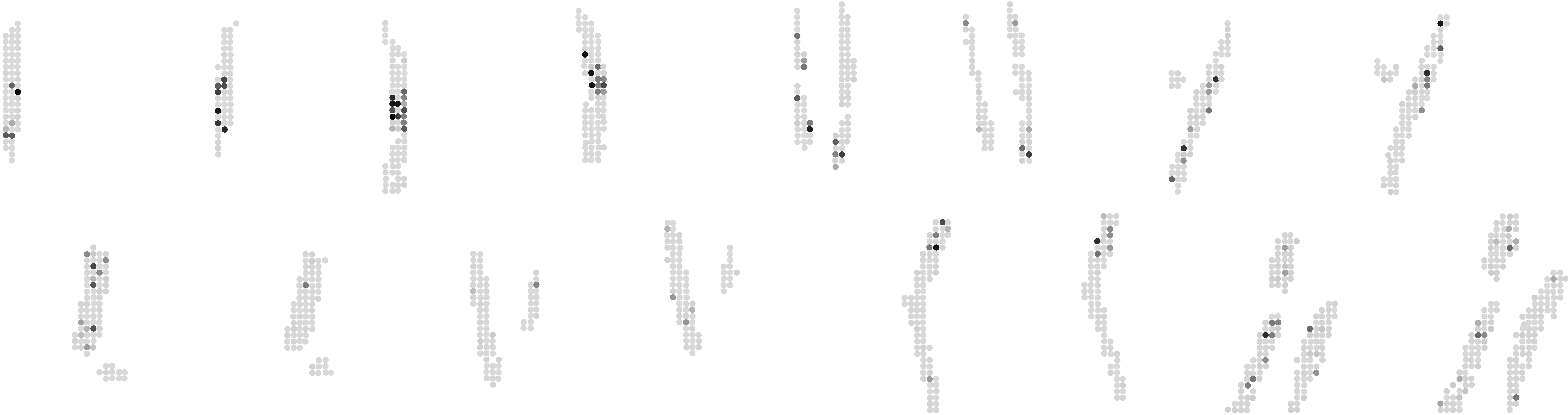

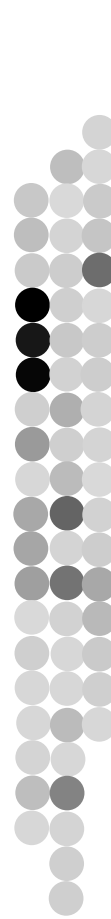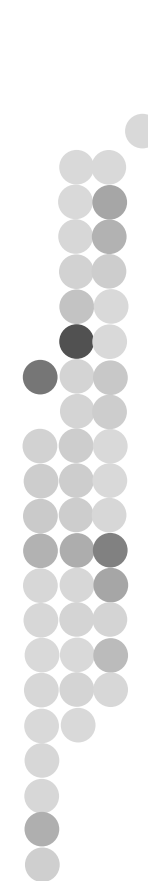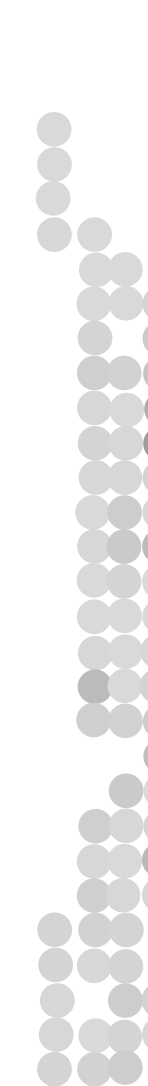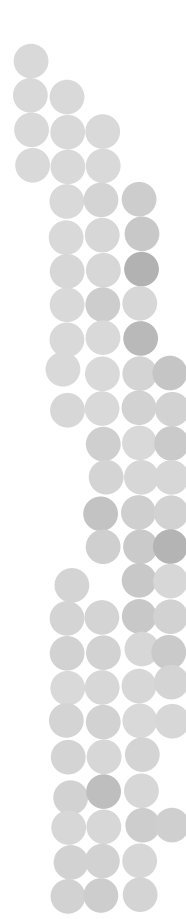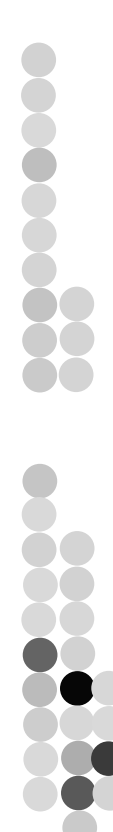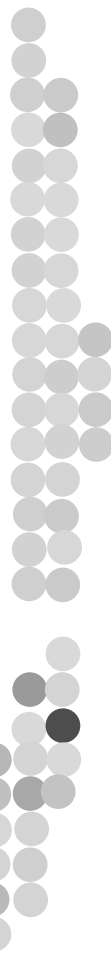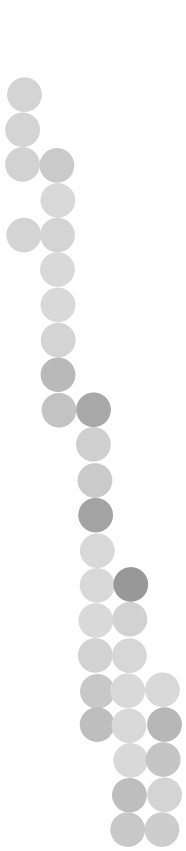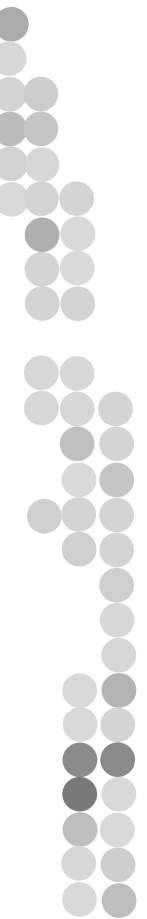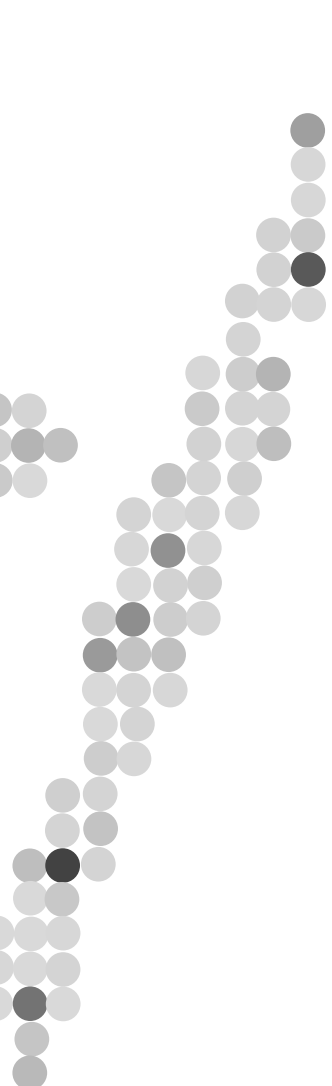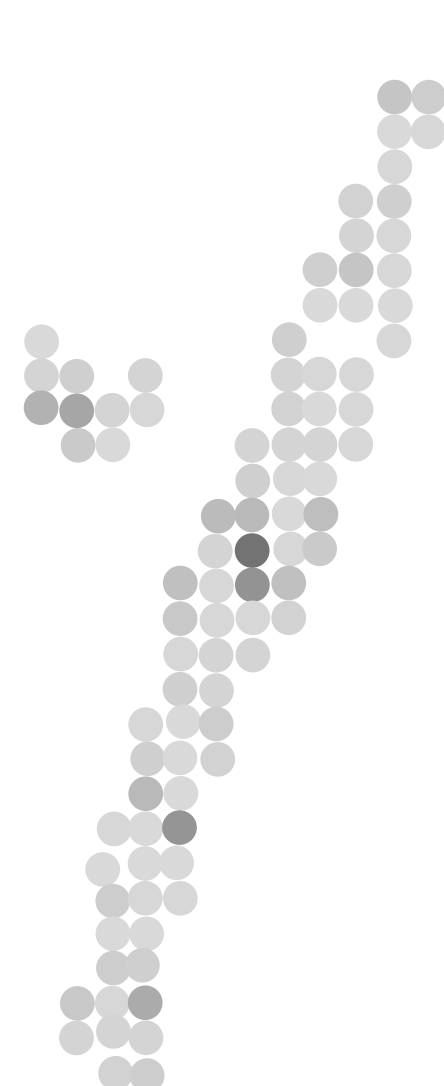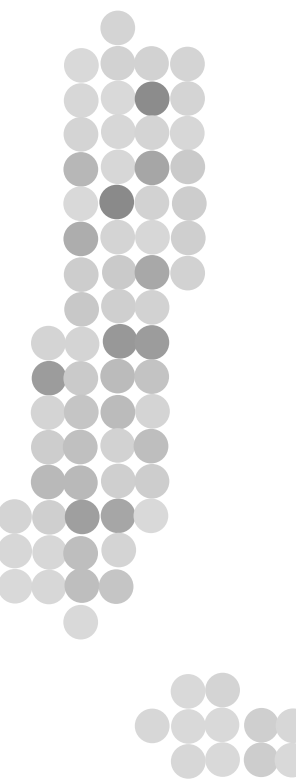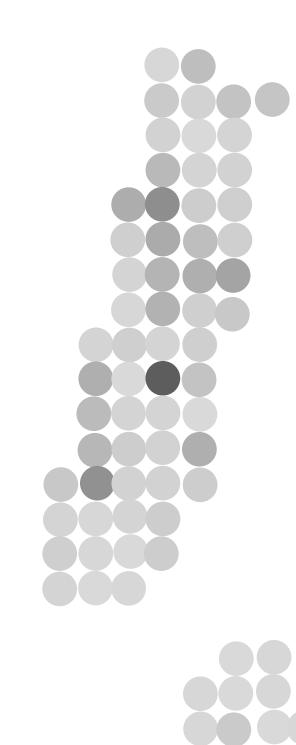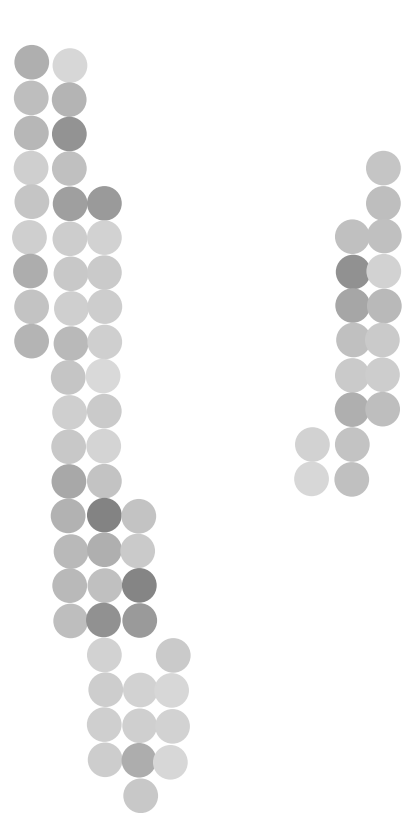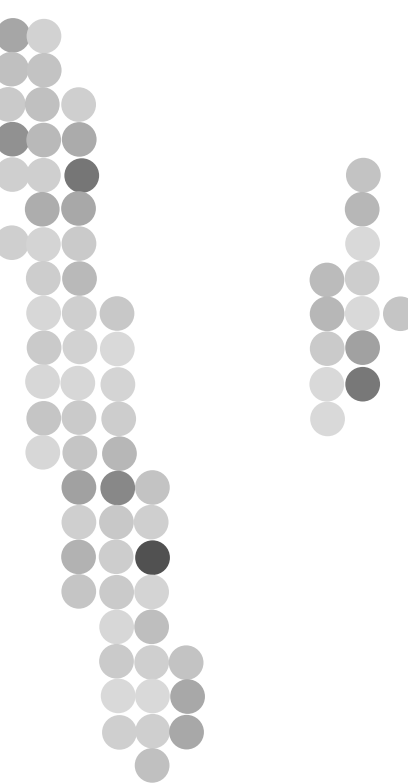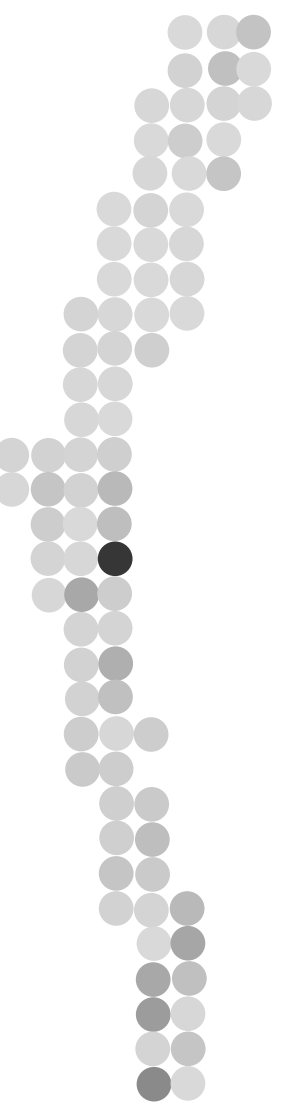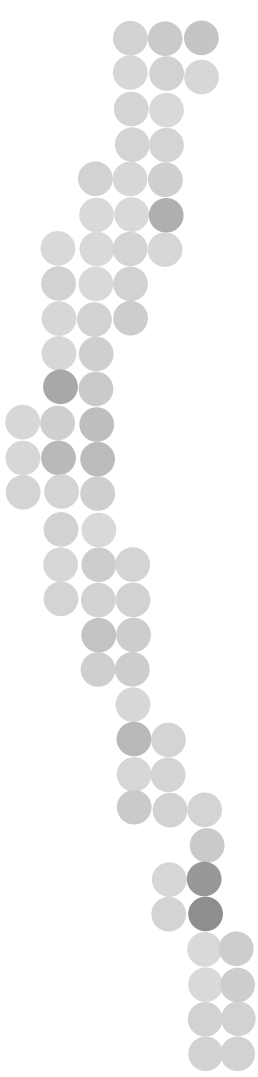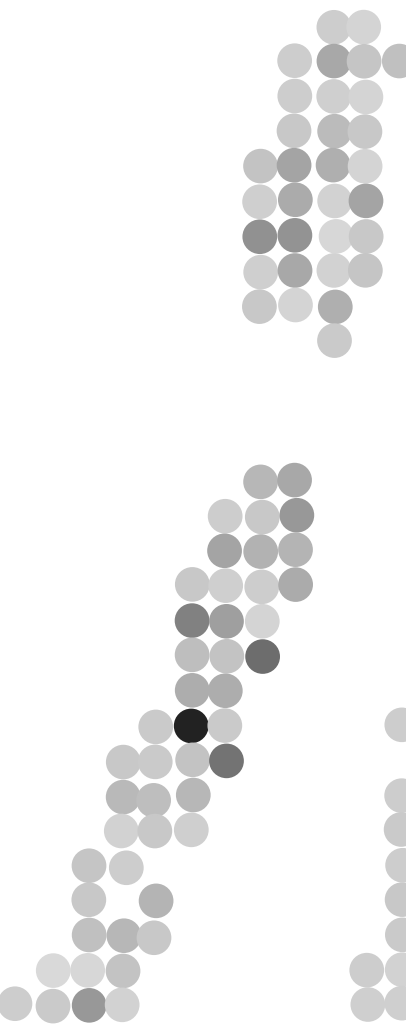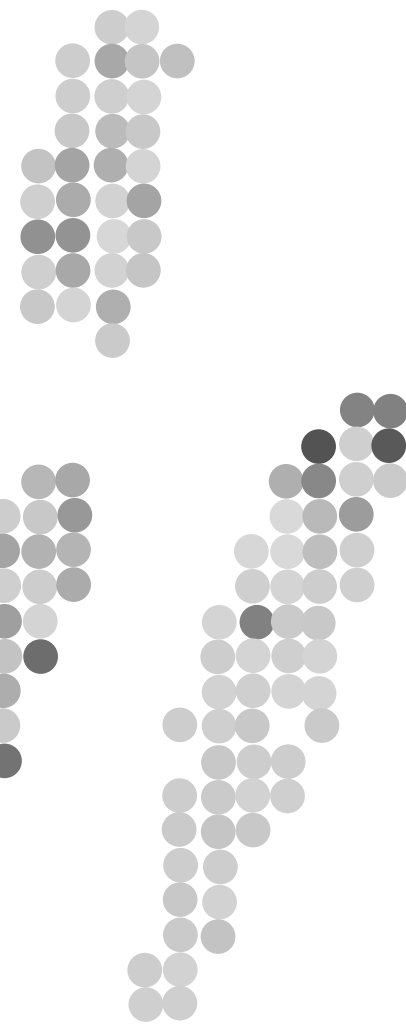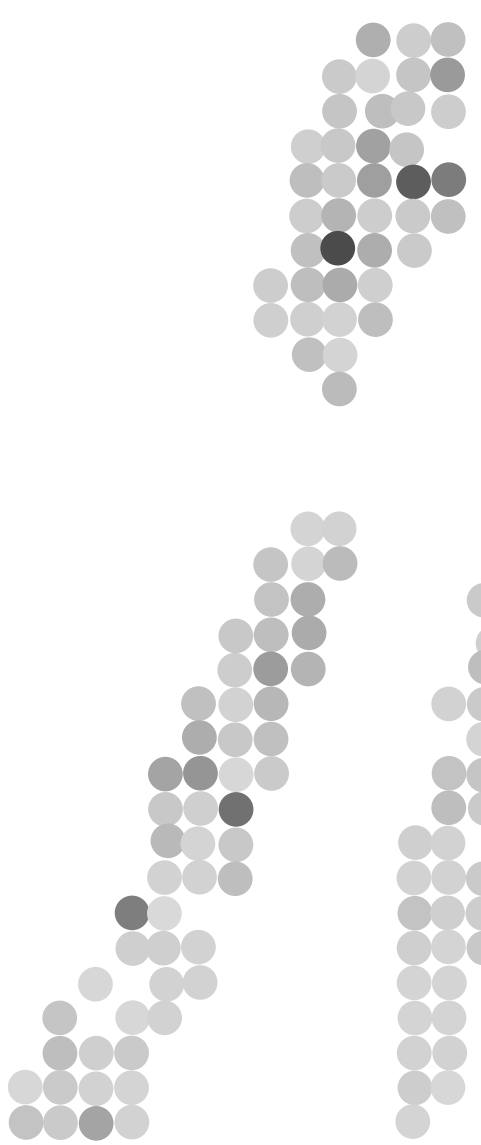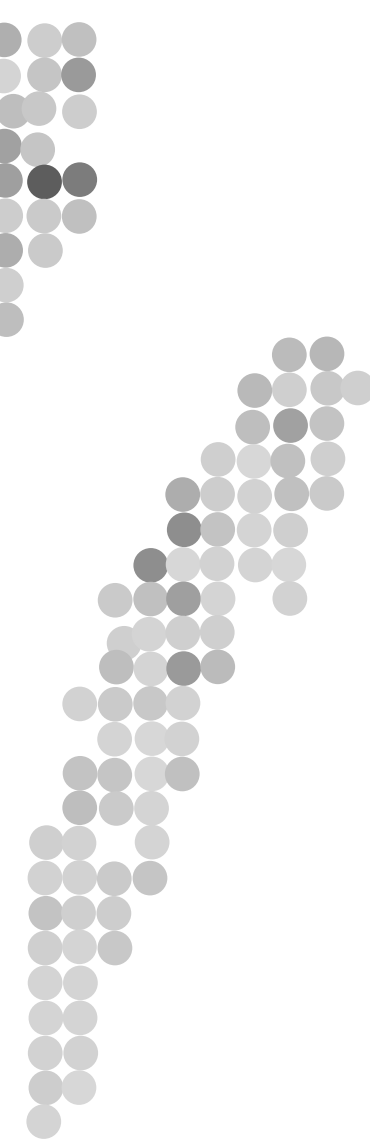

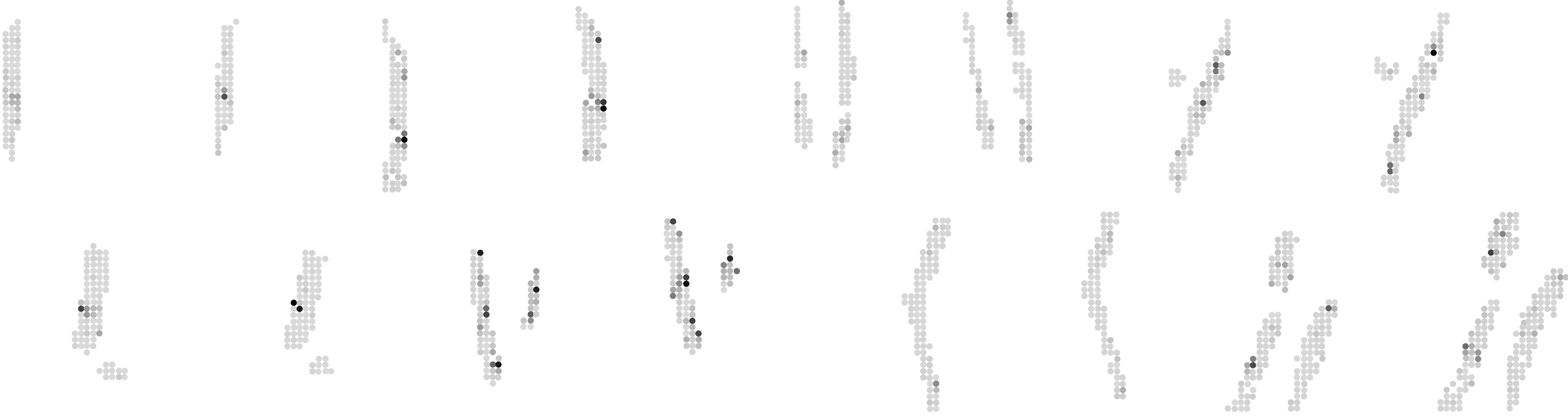

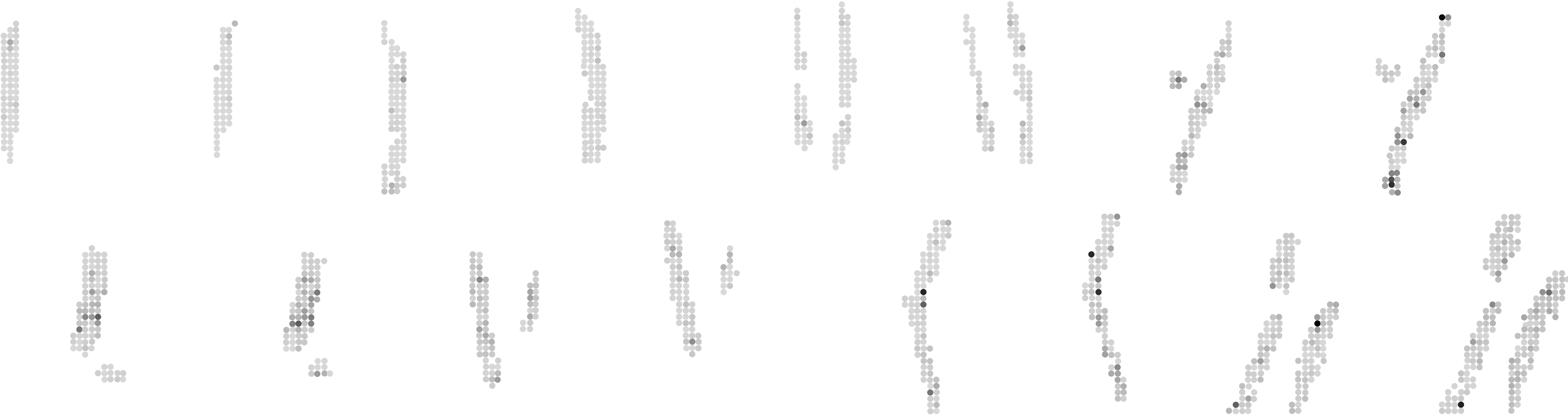

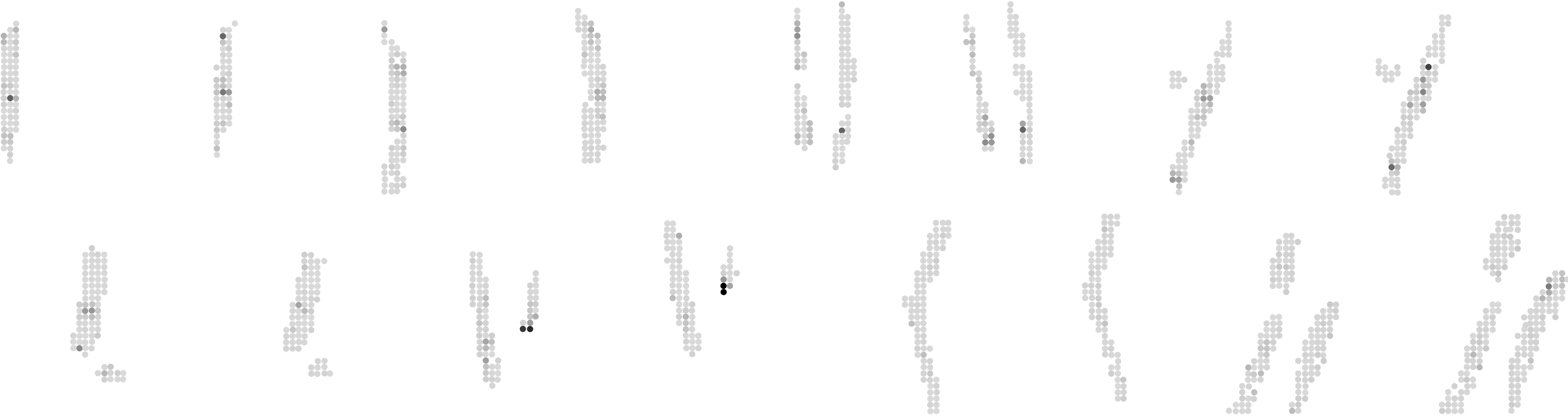

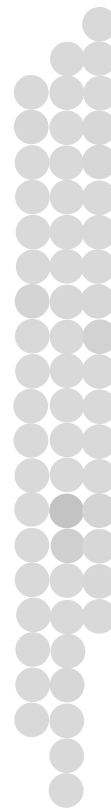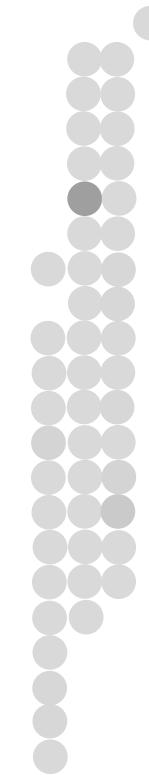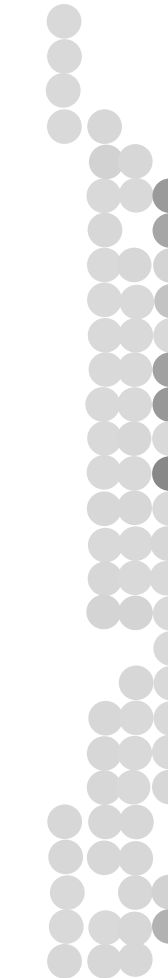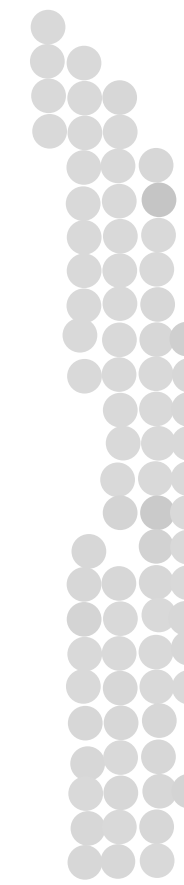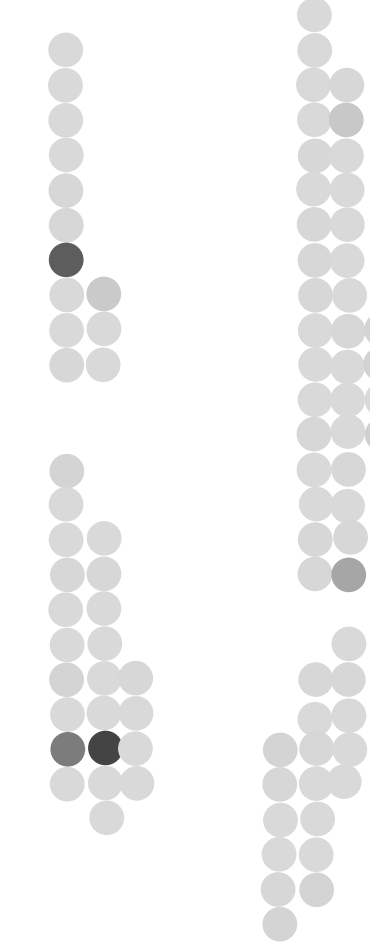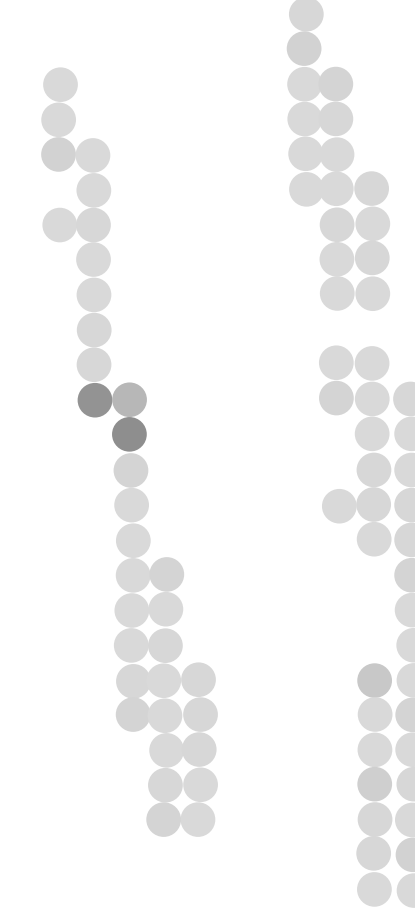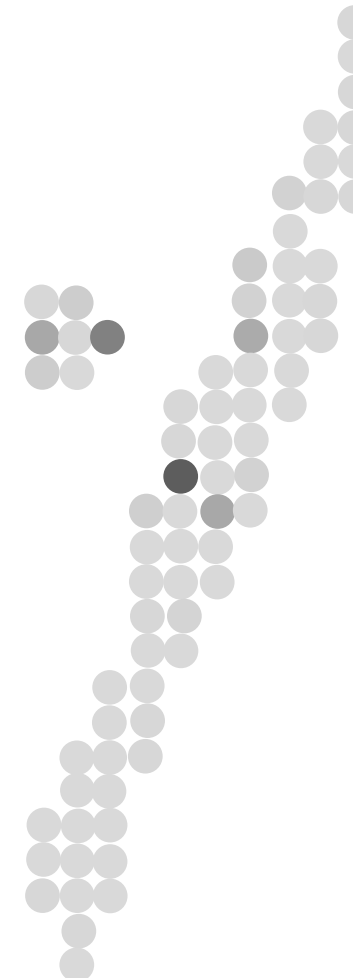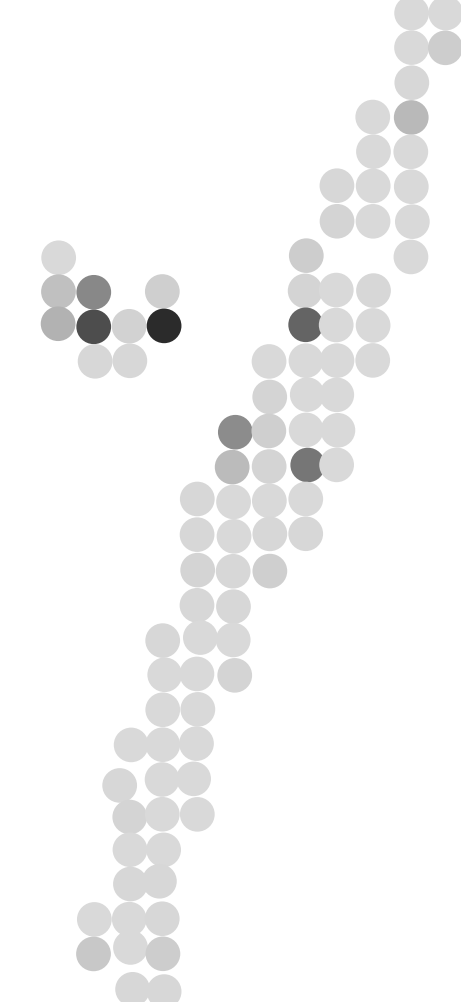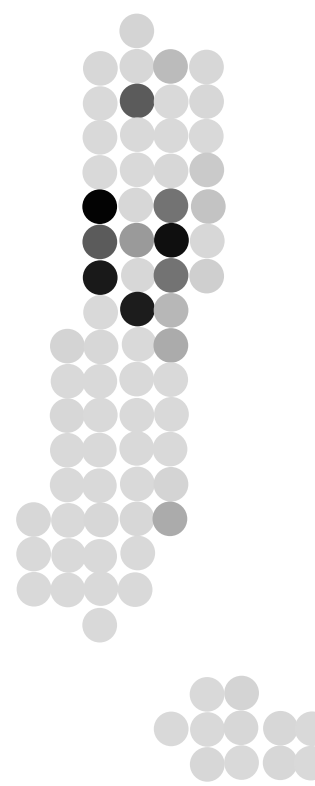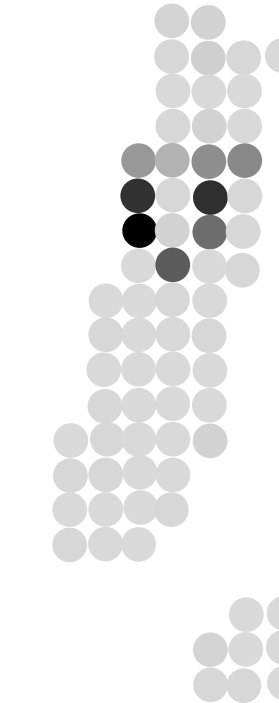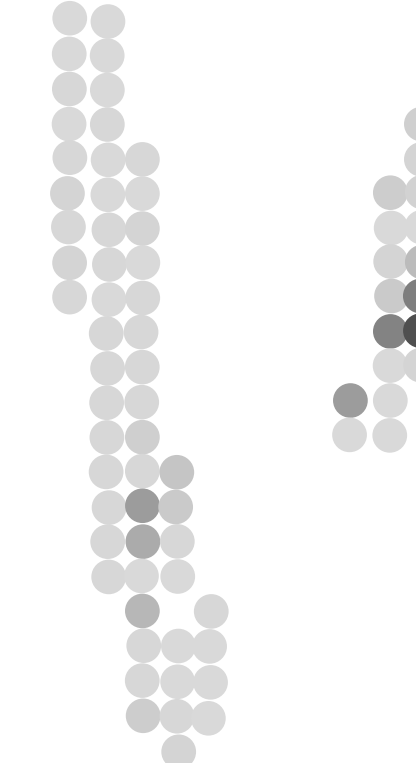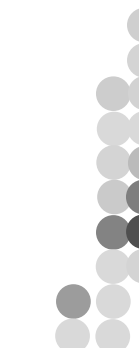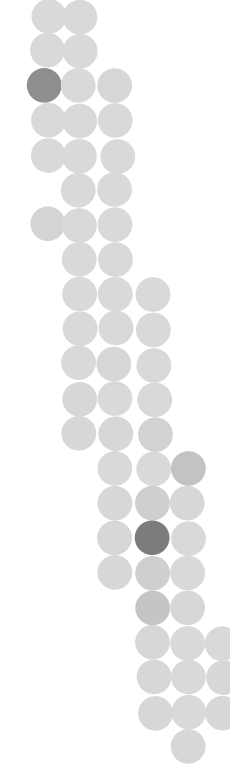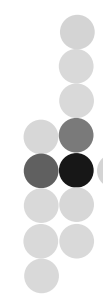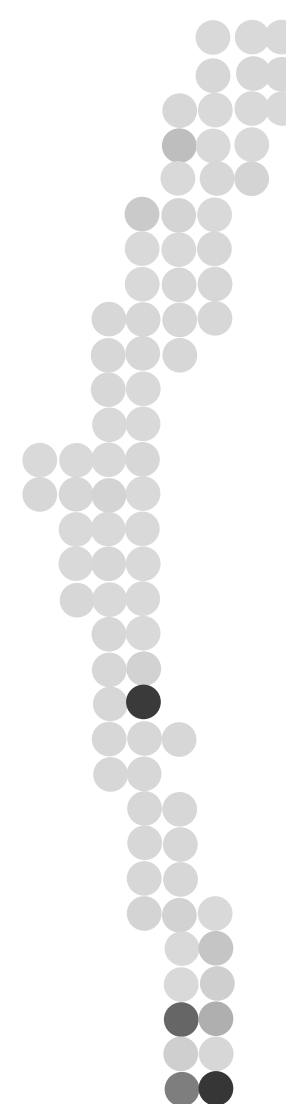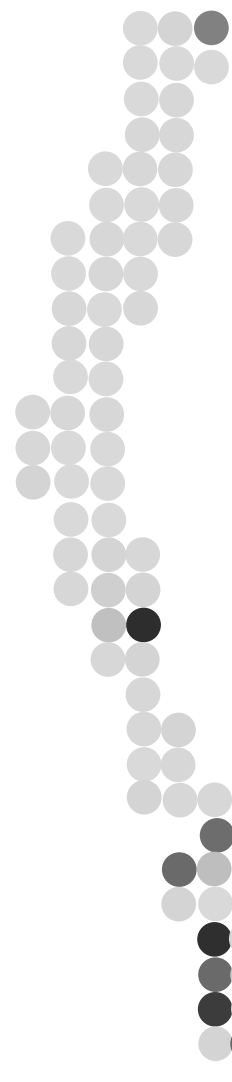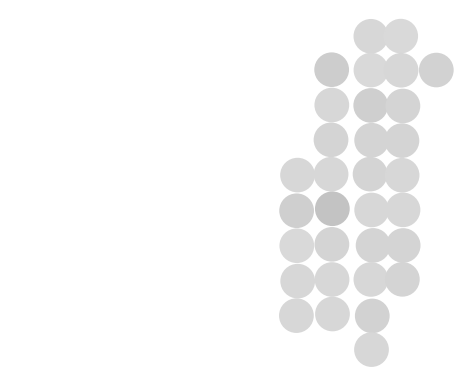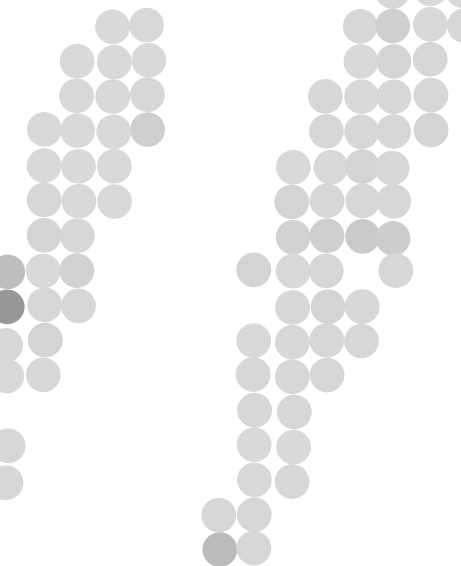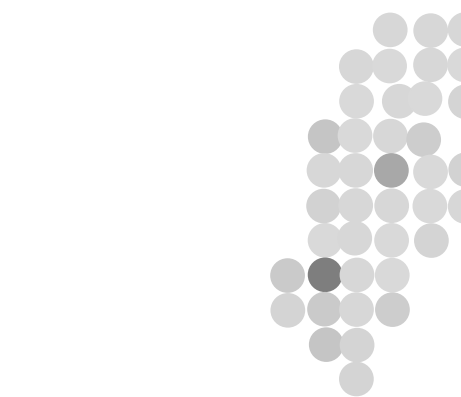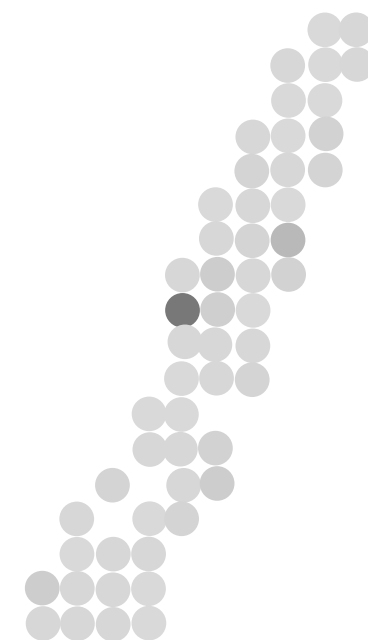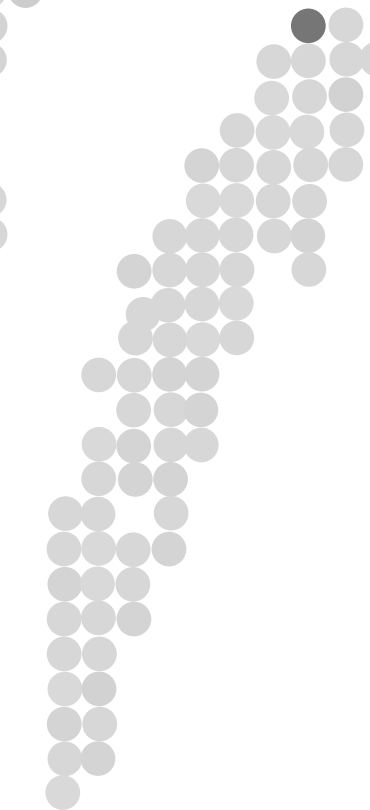

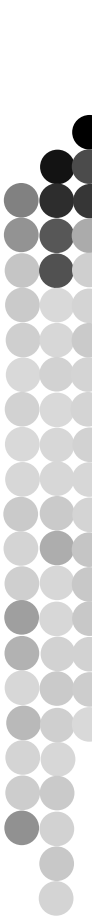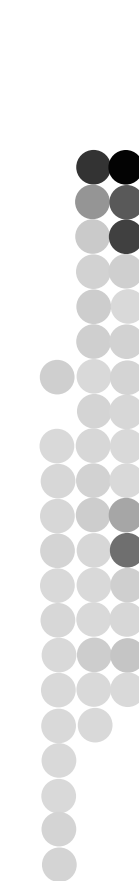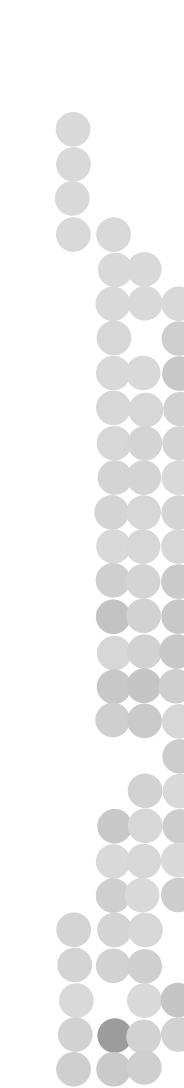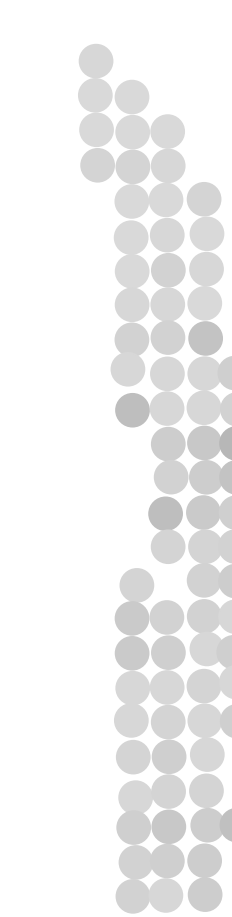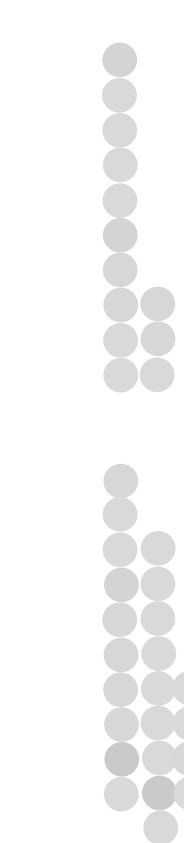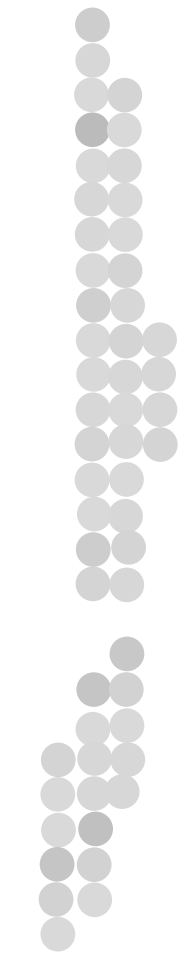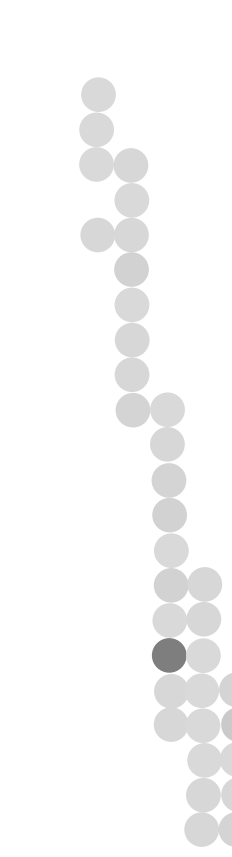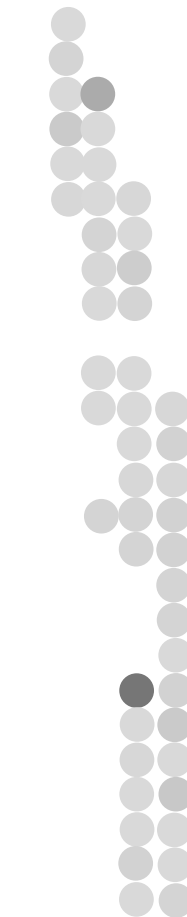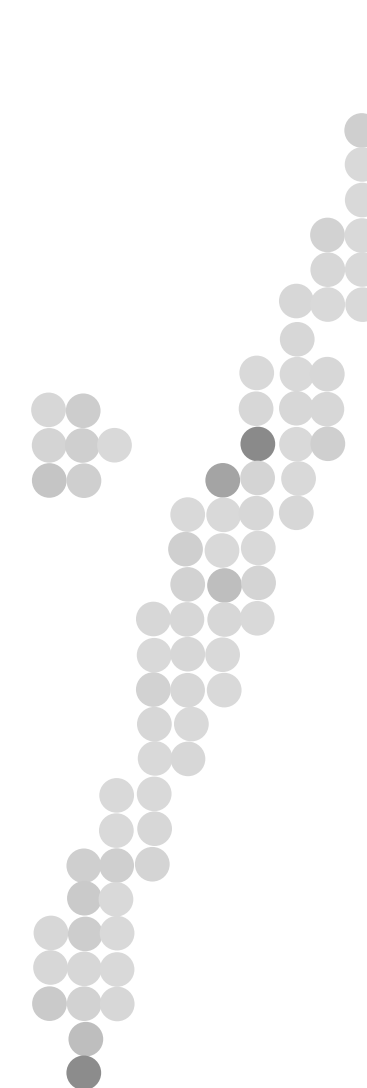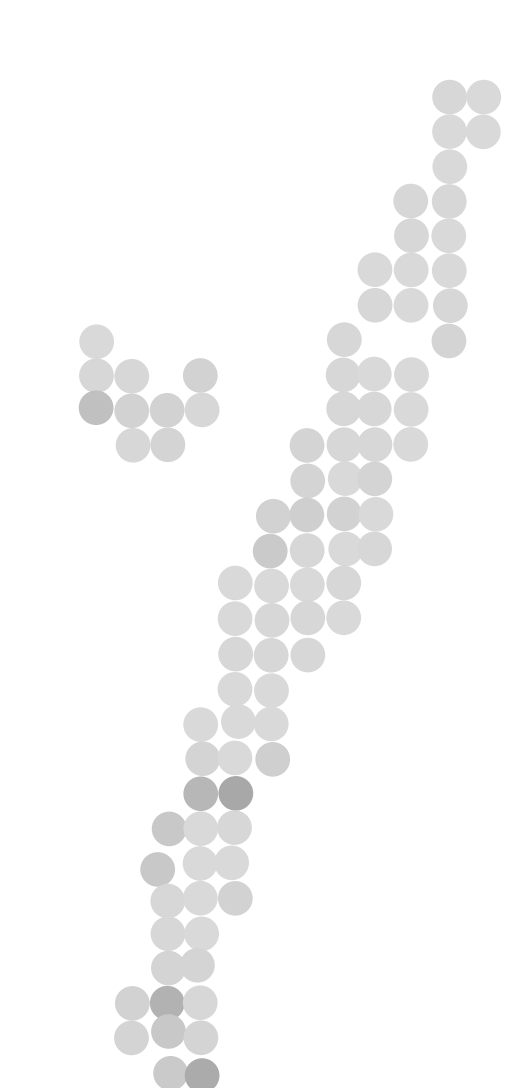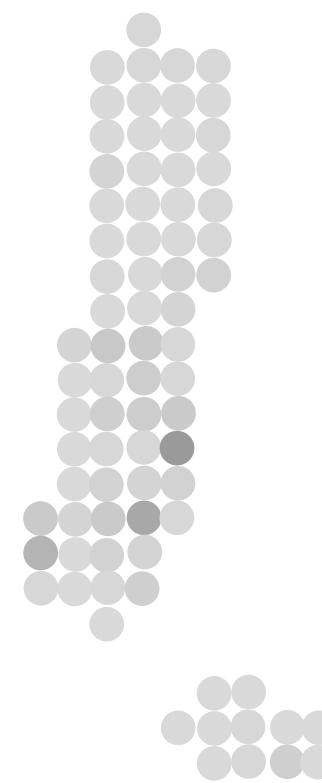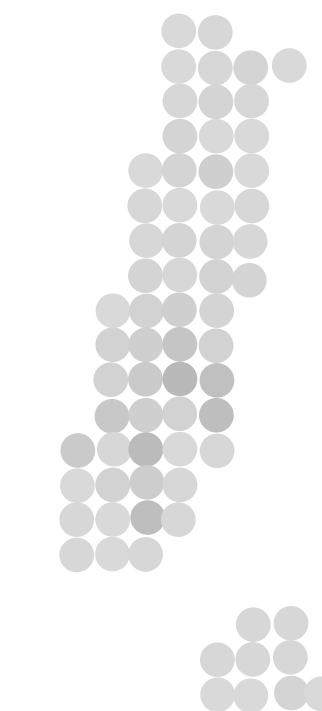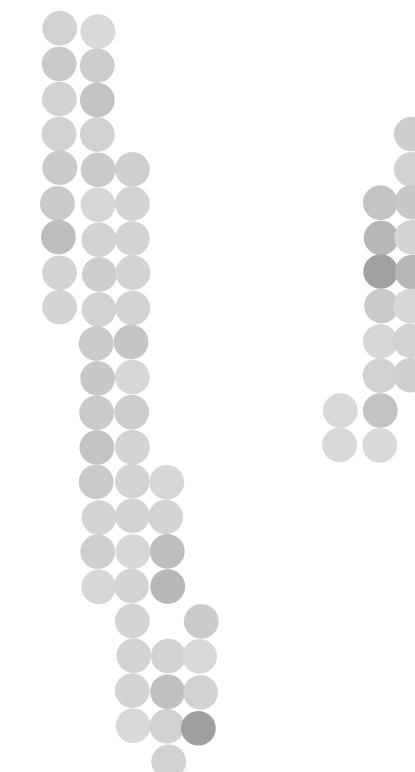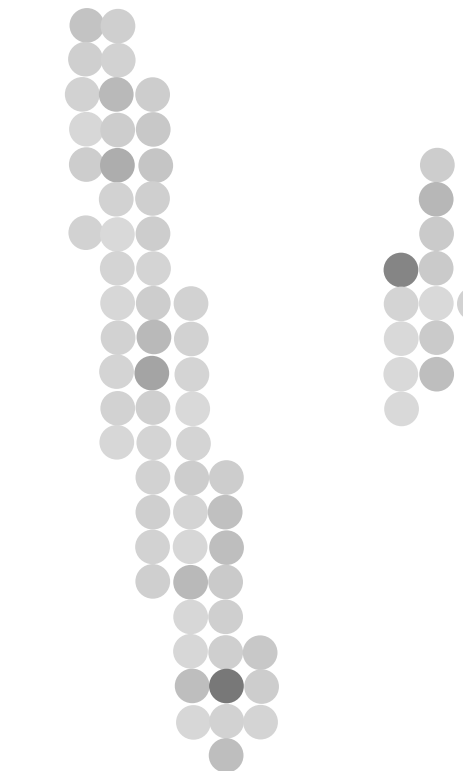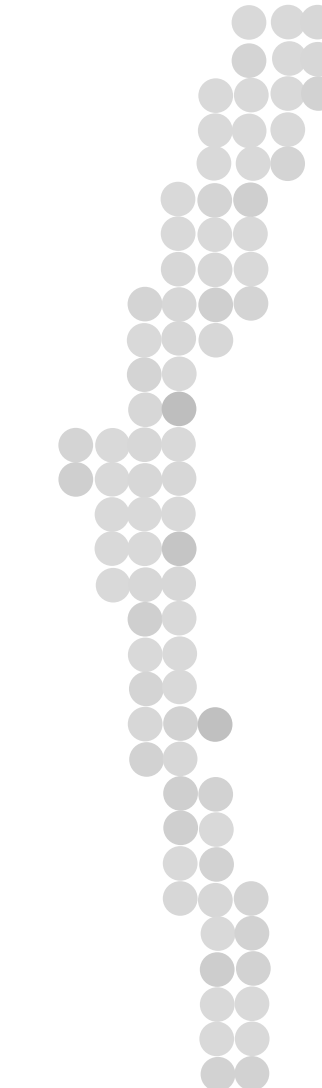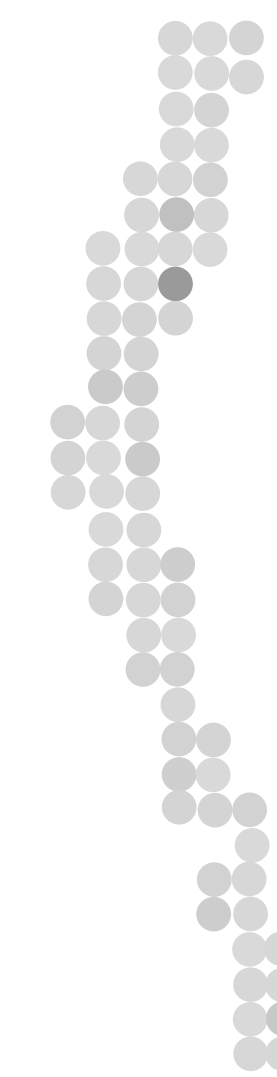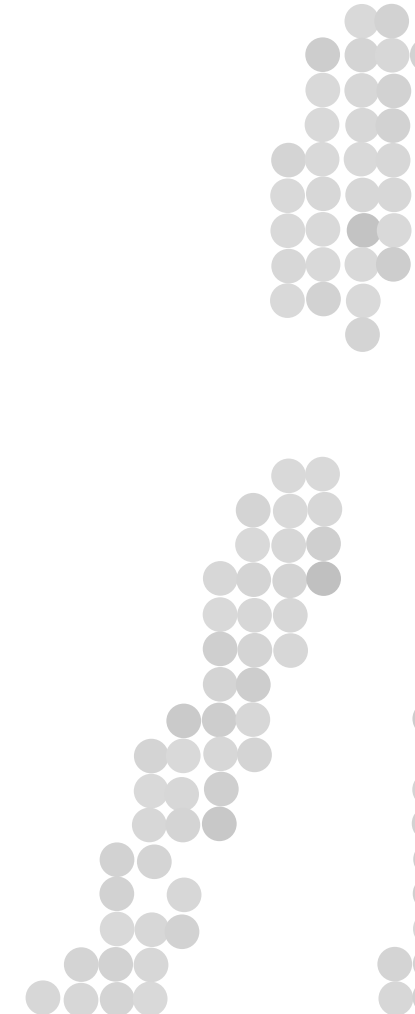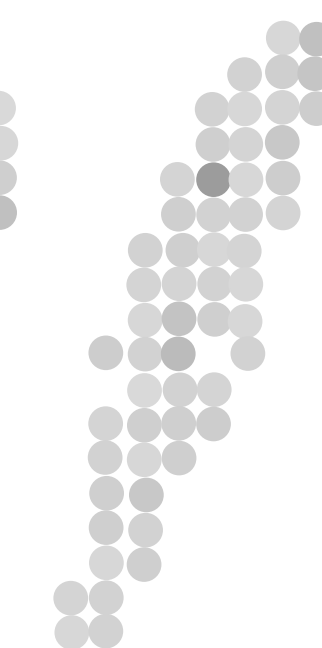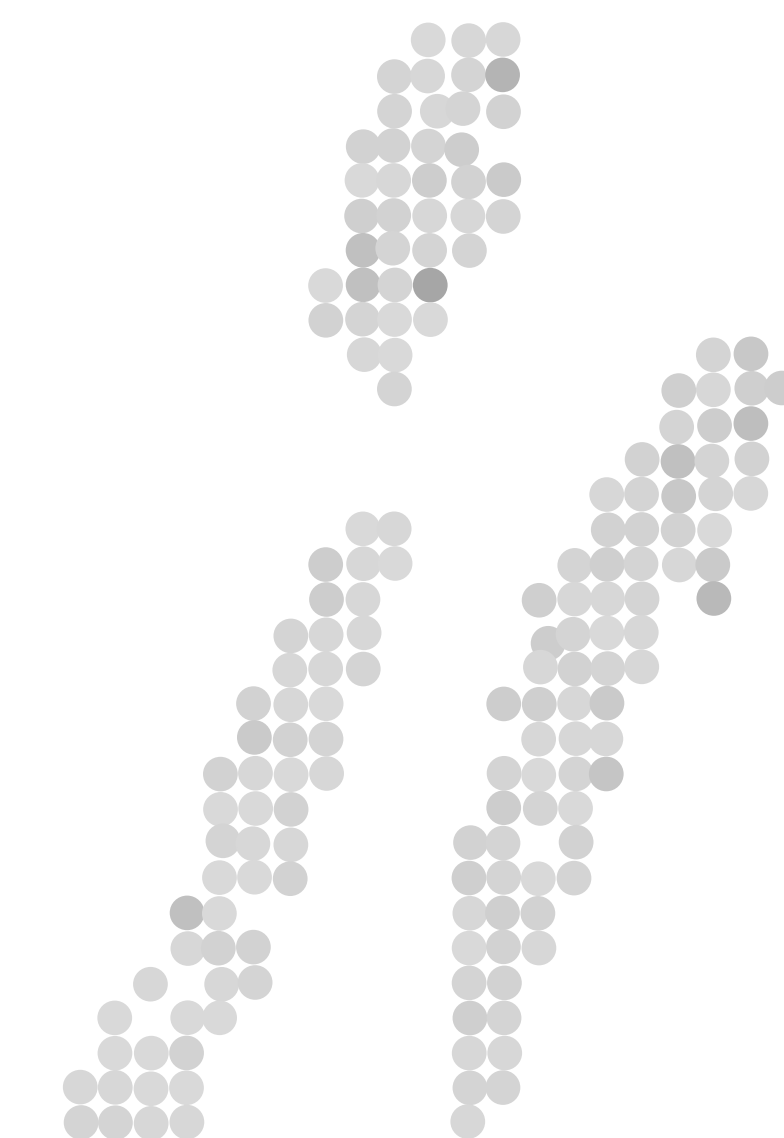

Supplement: Supplementary file 4 — Supplementary Dataset [file 41467_2022_33069_MOESM4_ESM.zip › Supplementary Data Files Nature Communications (Marklund et al. 2022)/Supplementary_Data_File1_STD_Patient1_ActivityMap_13_Factors.pdf]

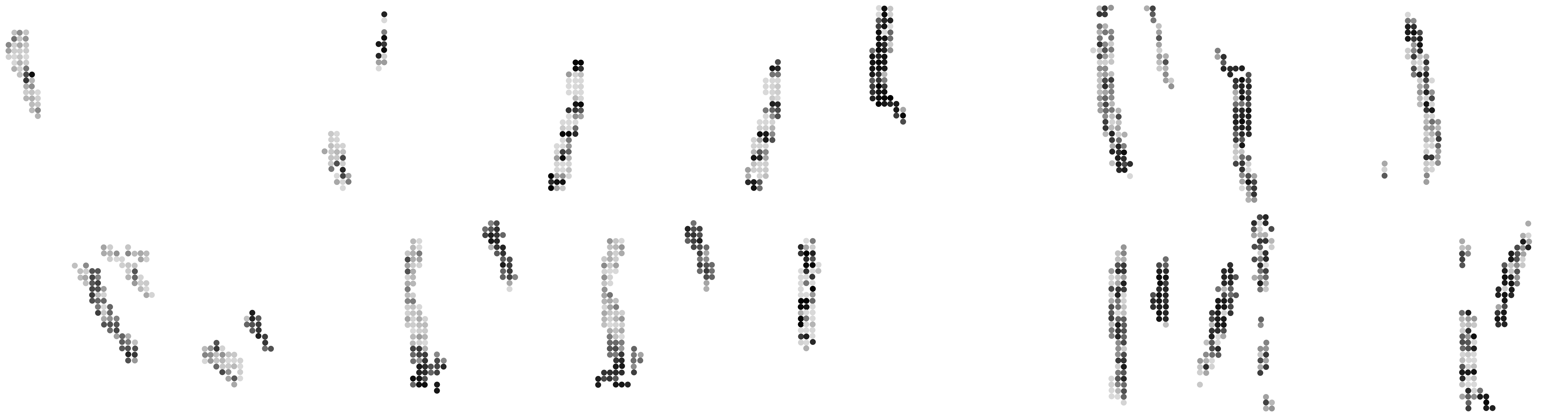

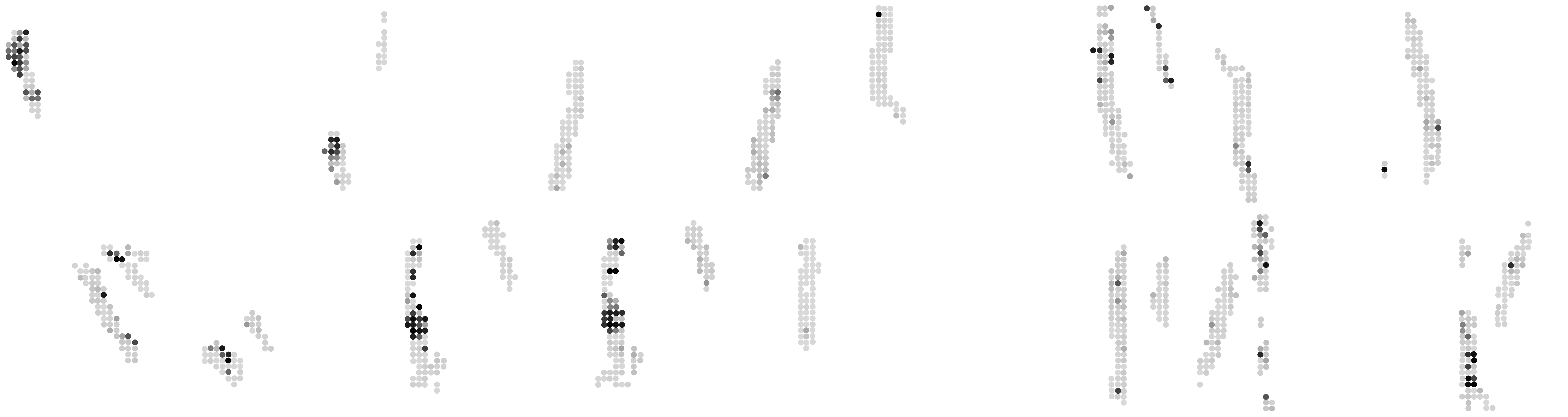

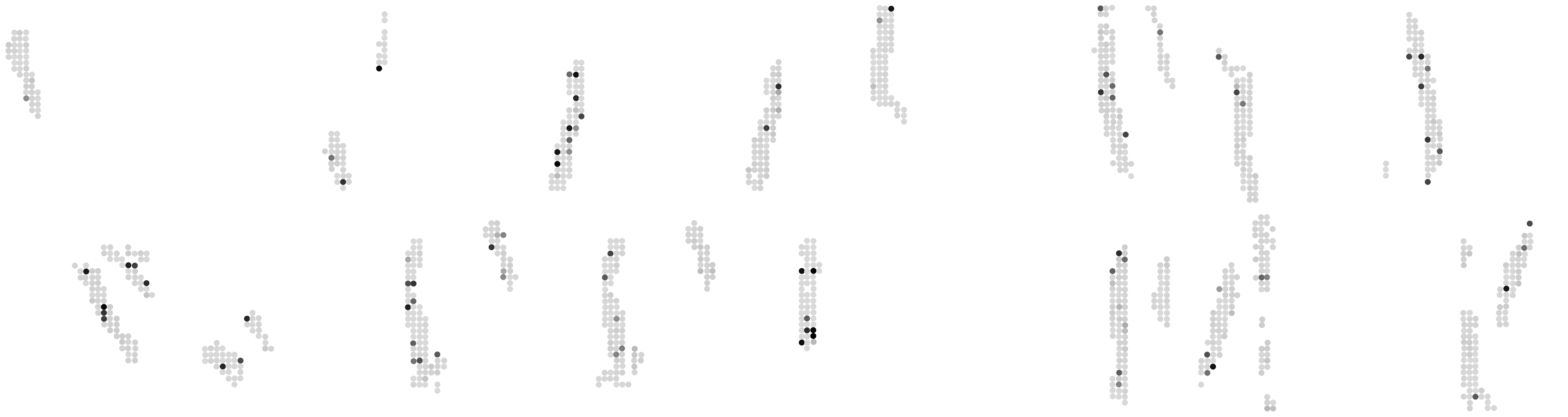

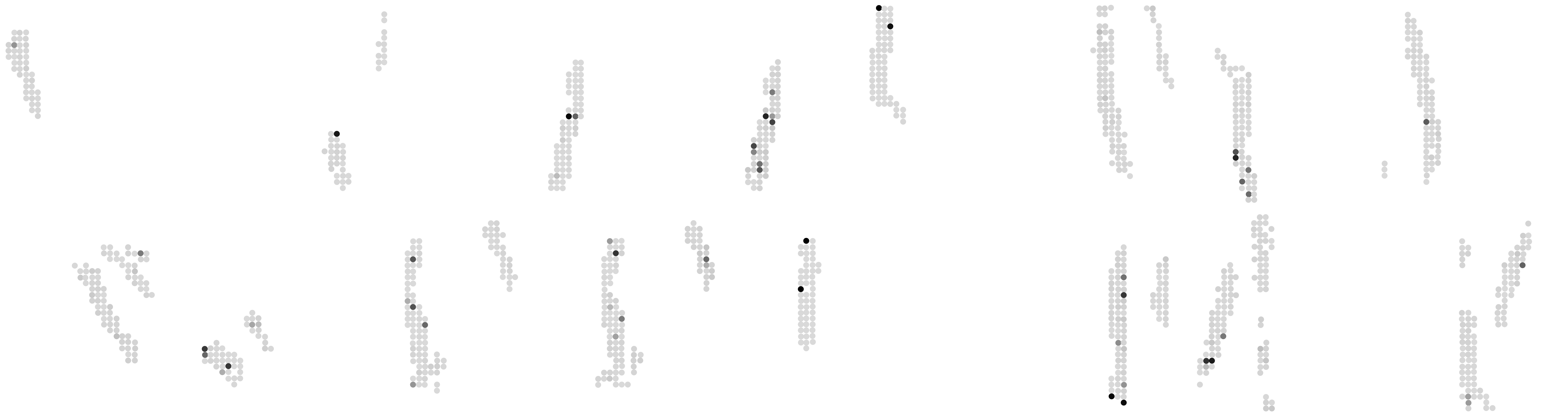

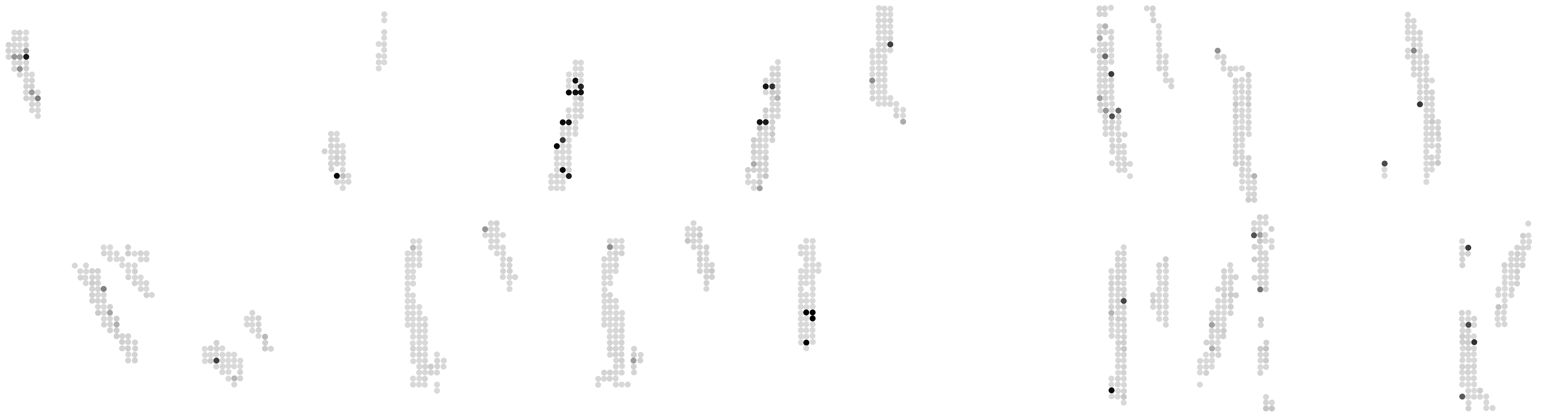

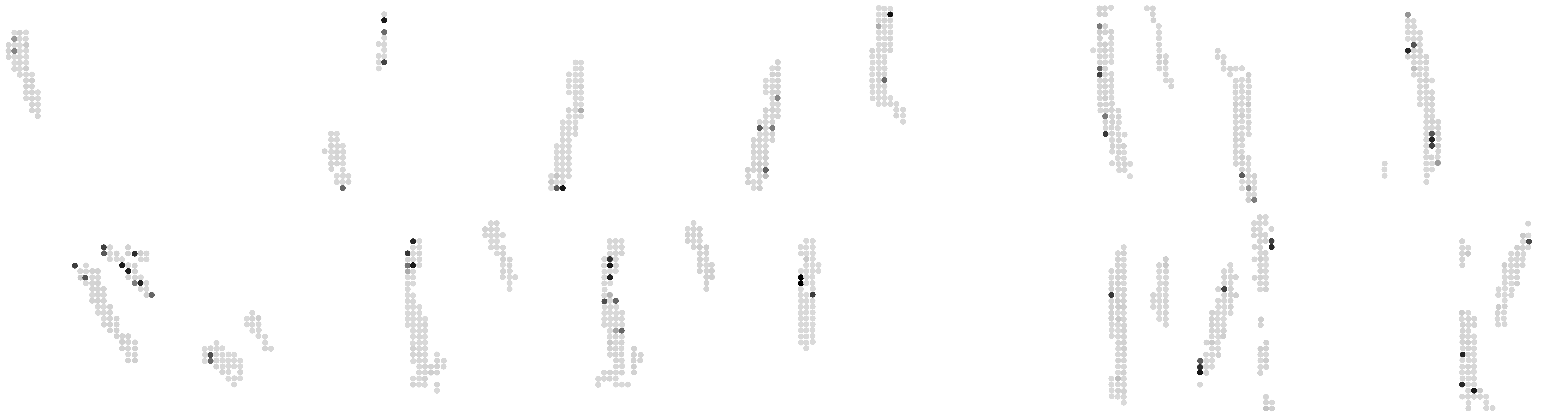

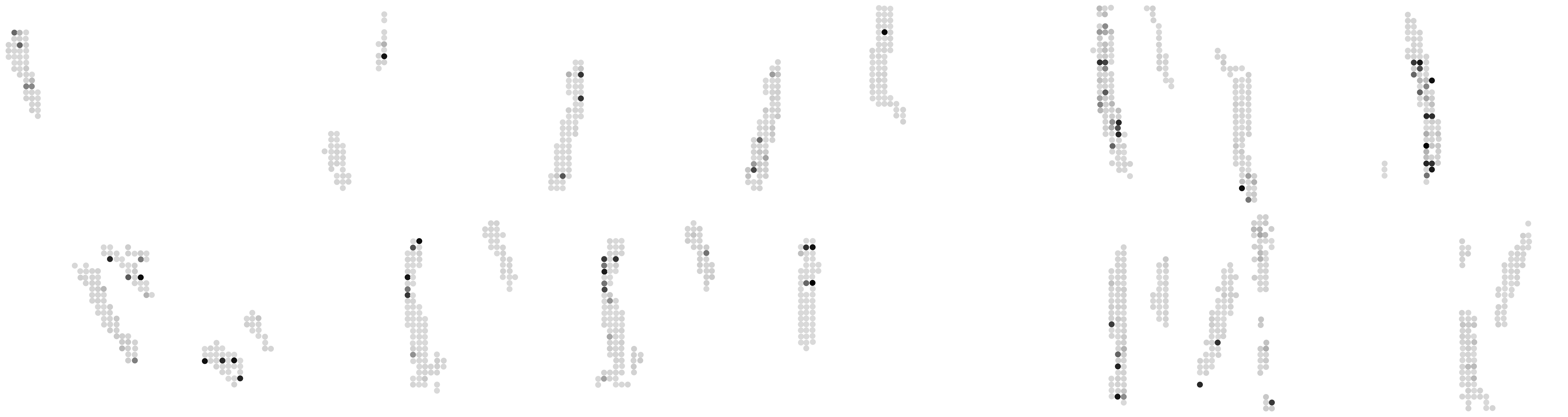

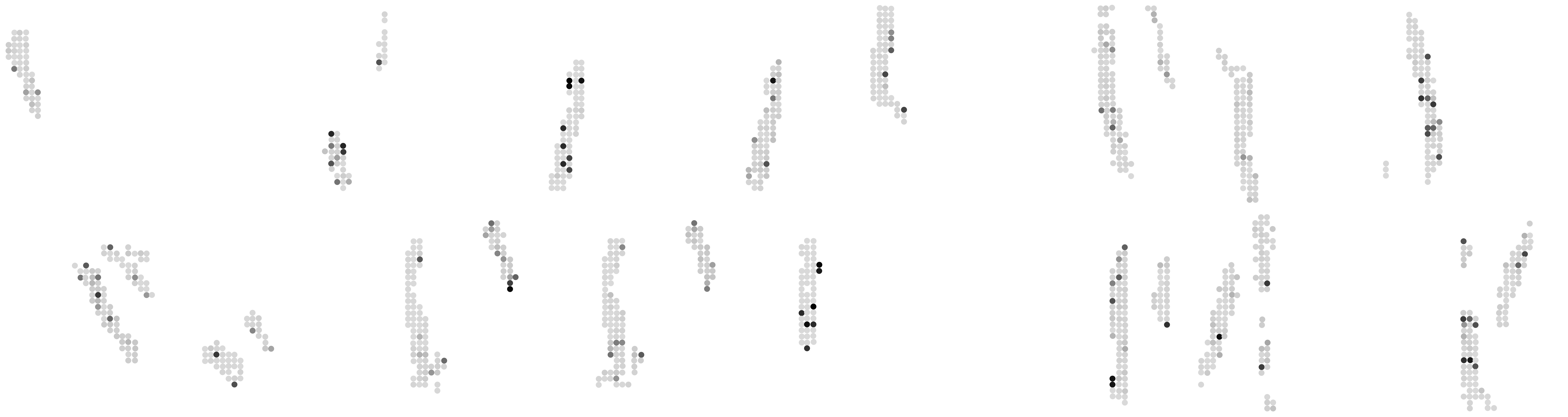

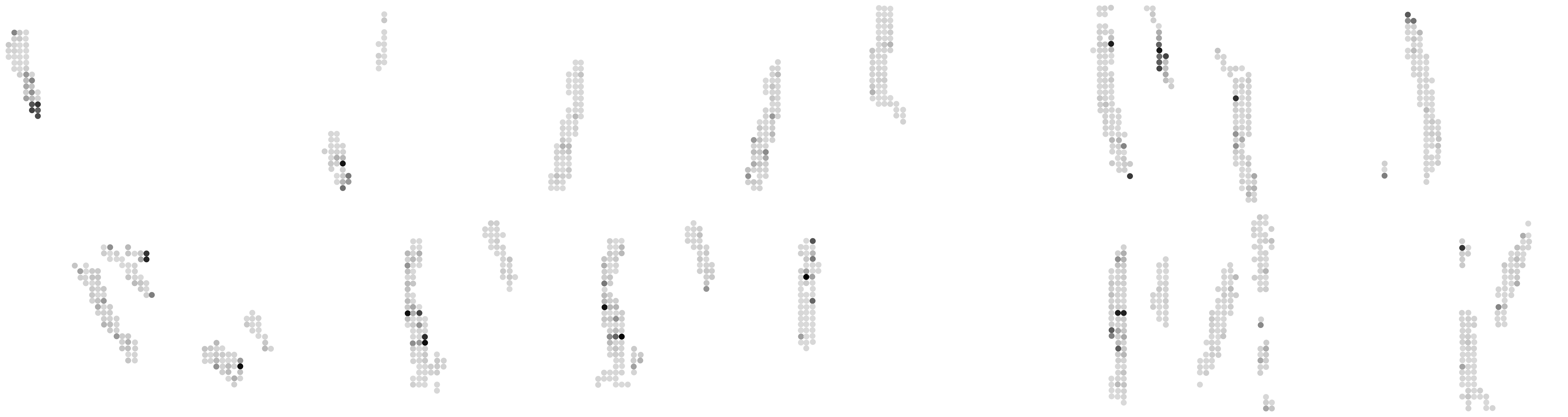

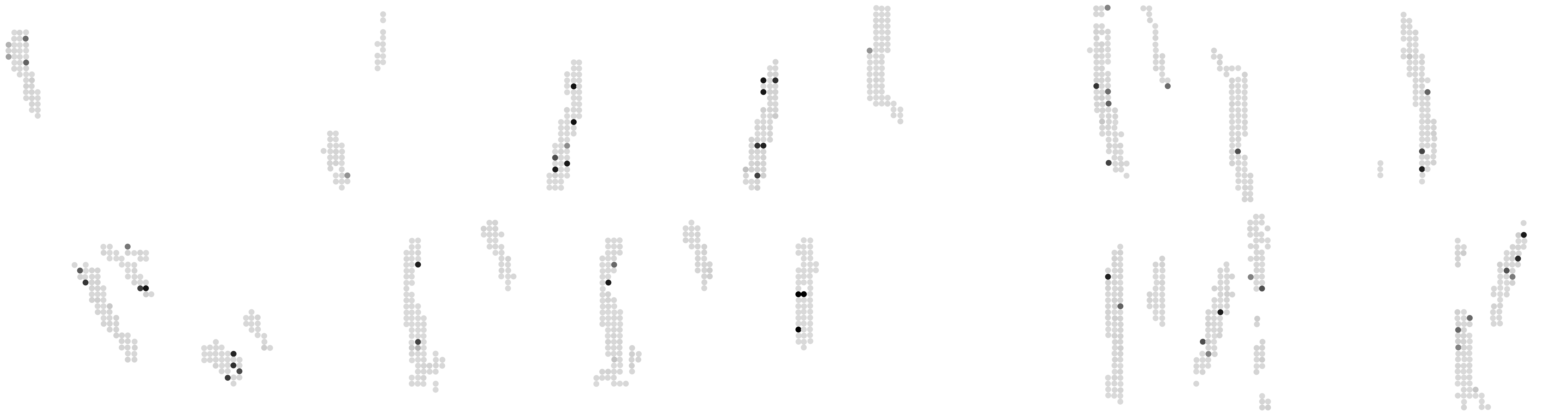

Supplement: Supplementary file 4 — Supplementary Dataset [file 41467_2022_33069_MOESM4_ESM.zip › Supplementary Data Files Nature Communications (Marklund et al. 2022)/Supplementary_Data_File5_STD_Patient3_ActivityMap_10_Factors.pdf]

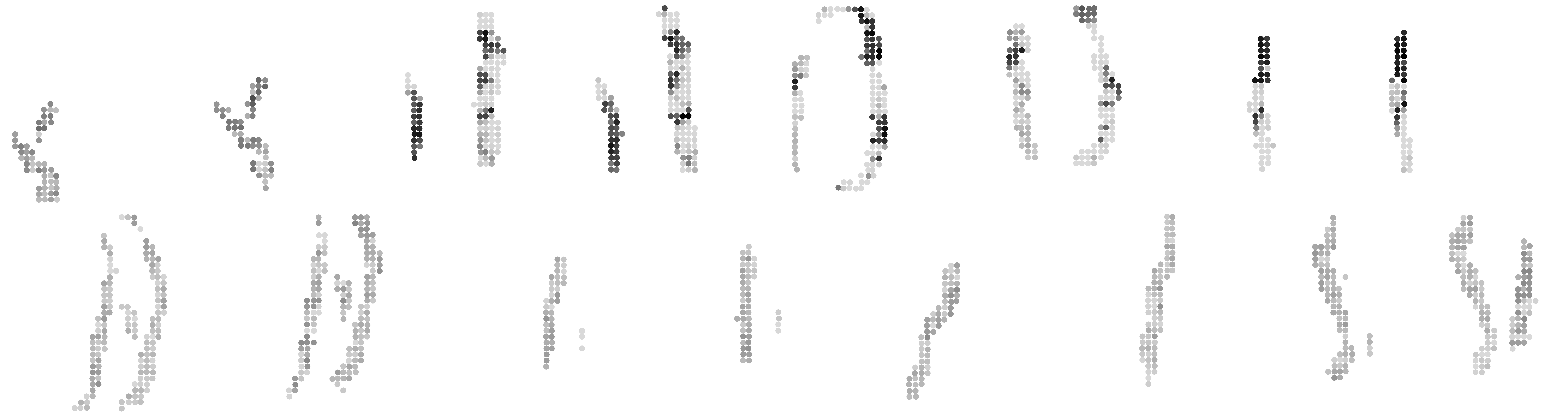

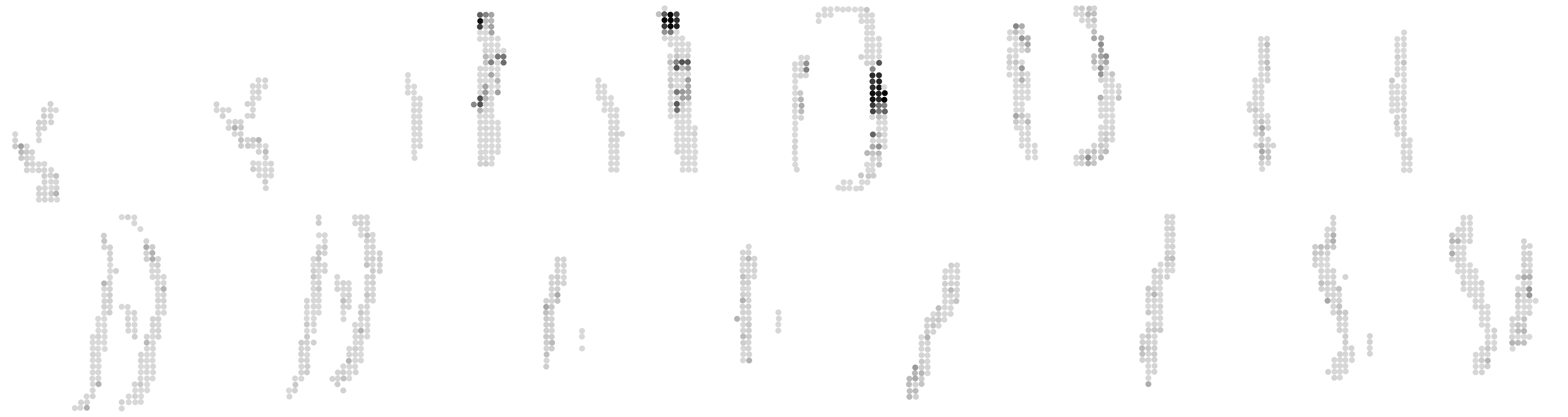

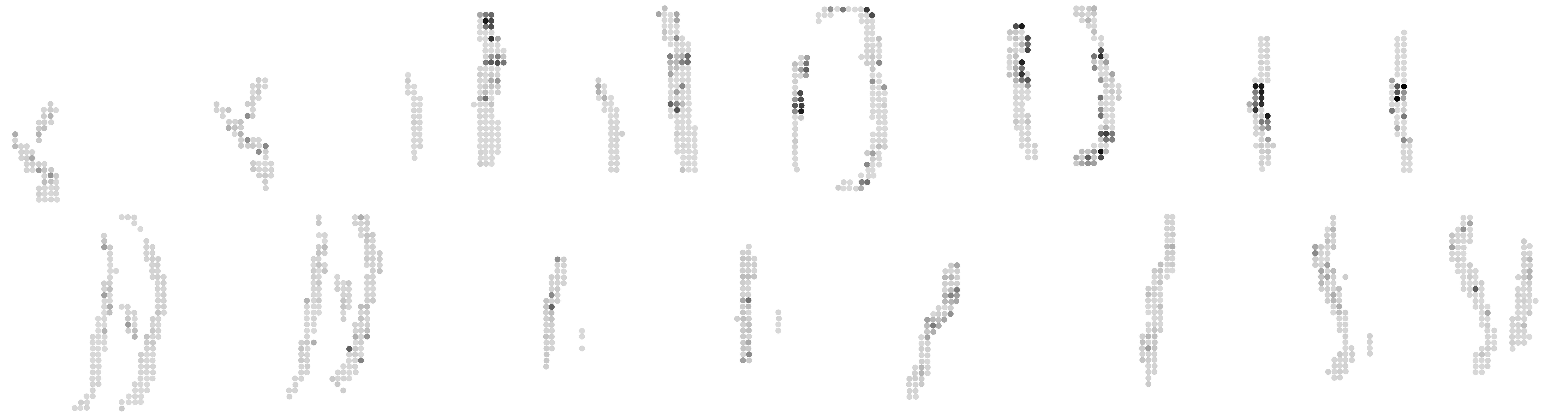

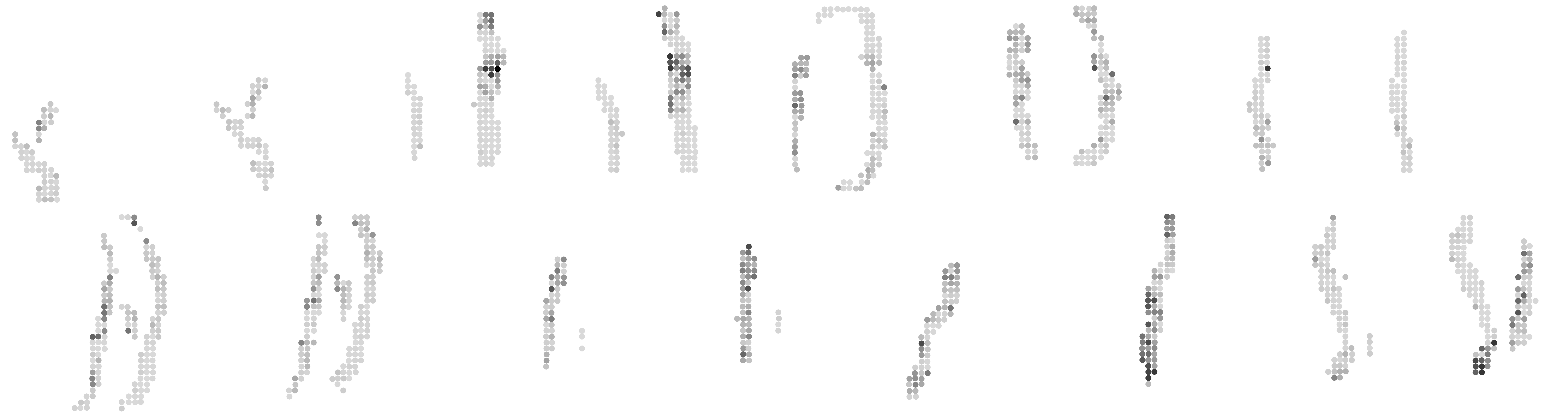

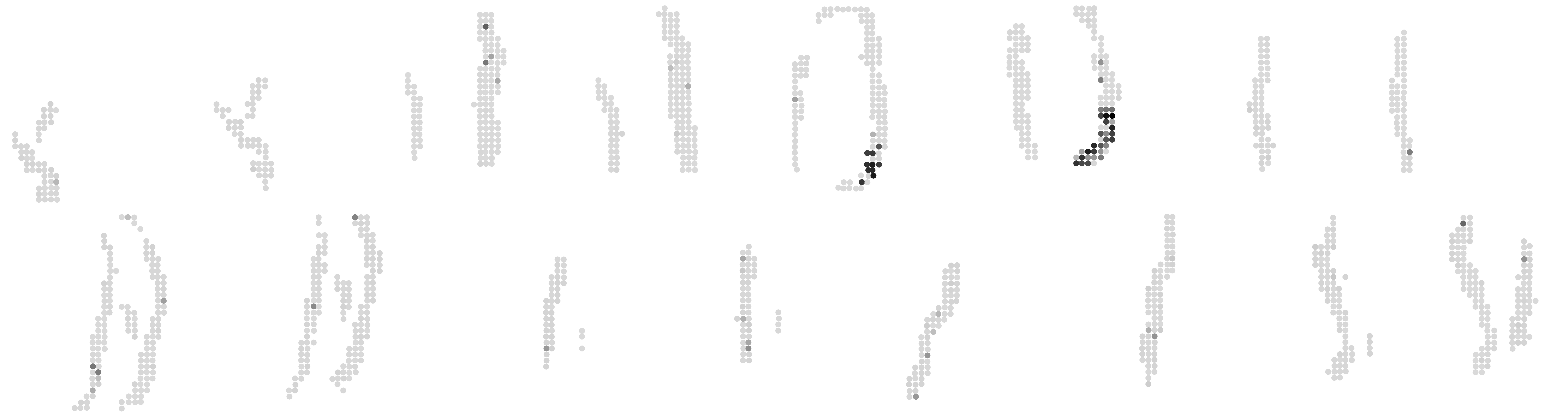

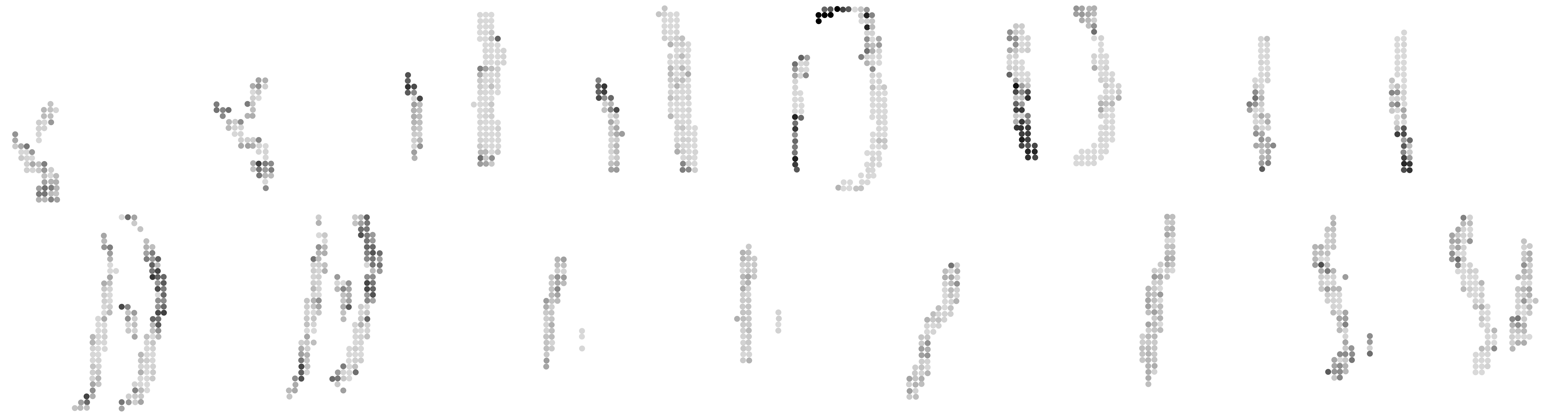

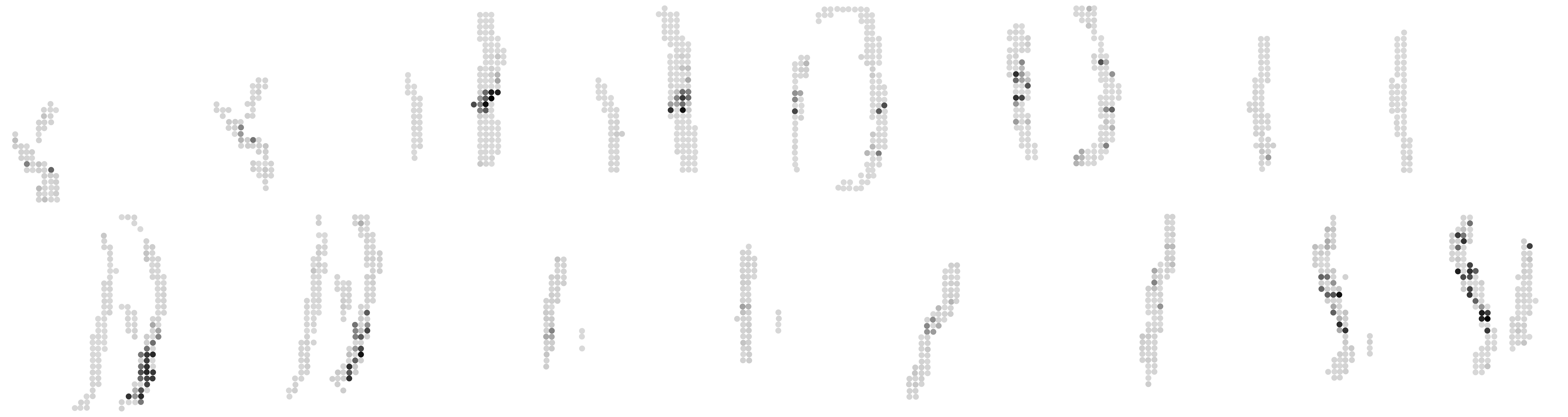

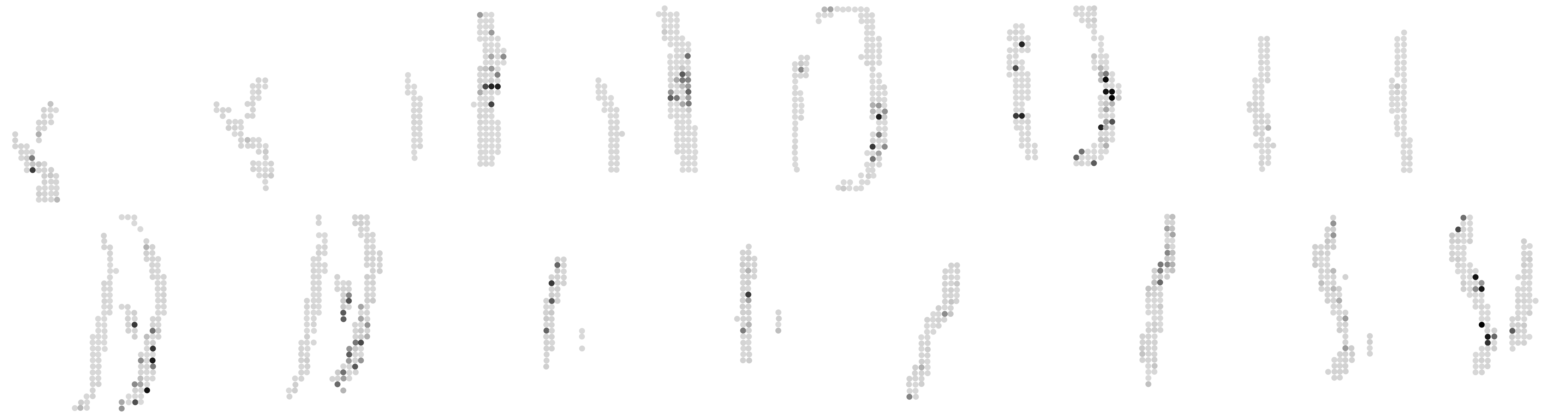

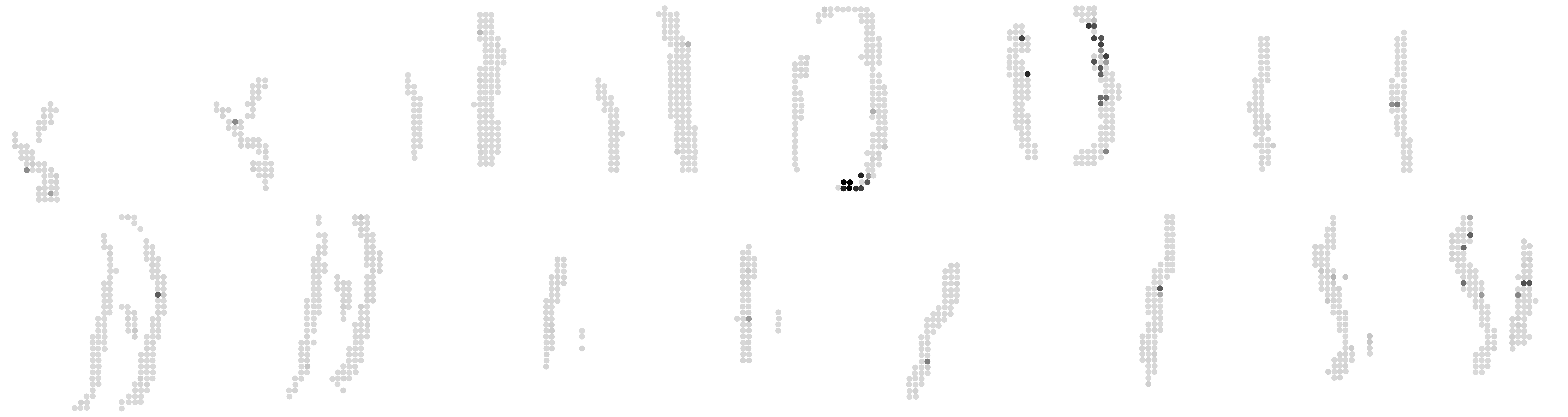

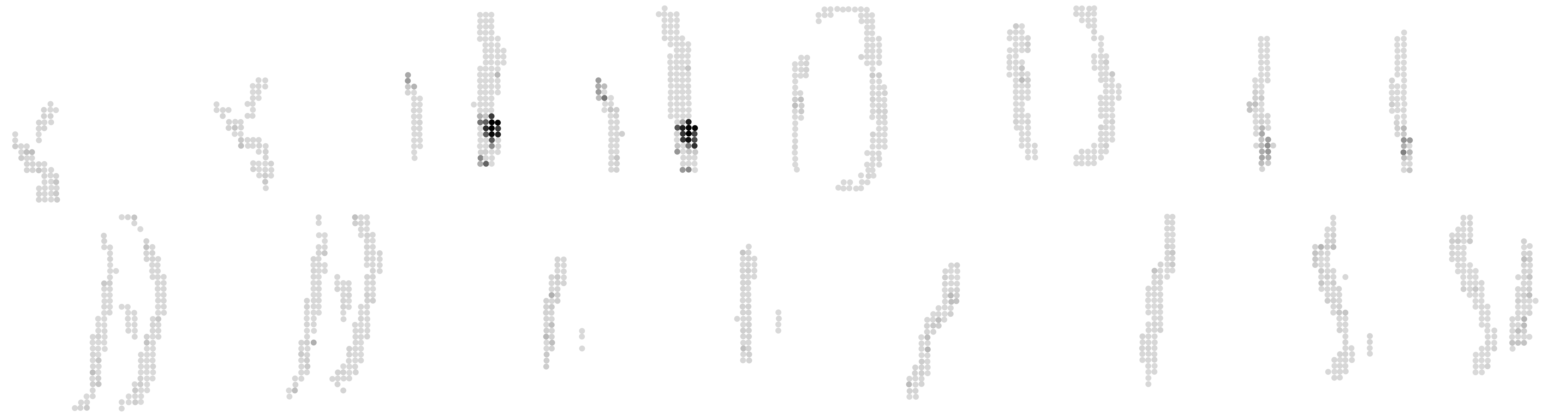

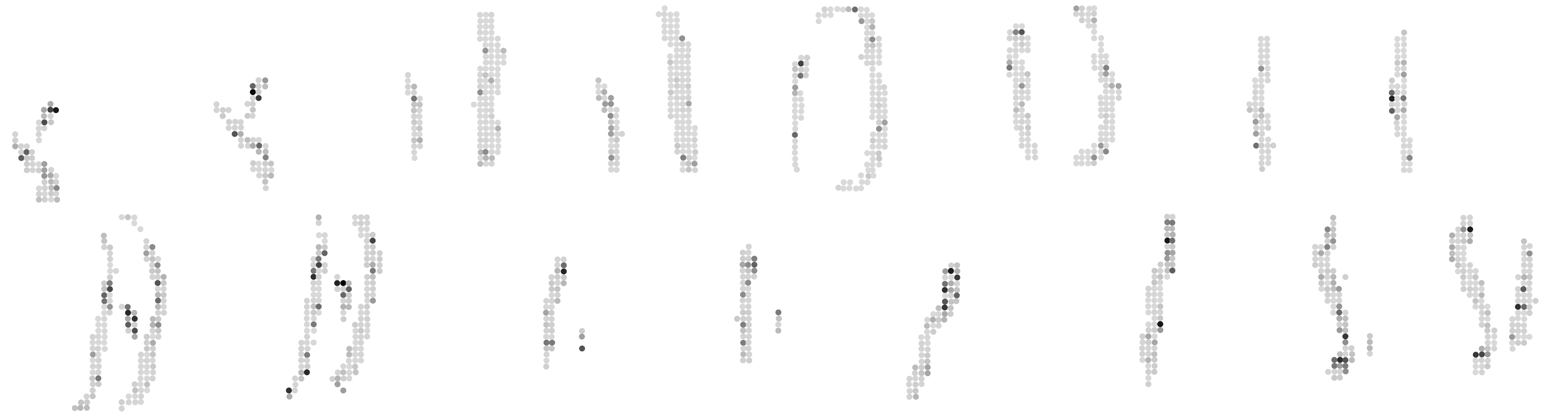

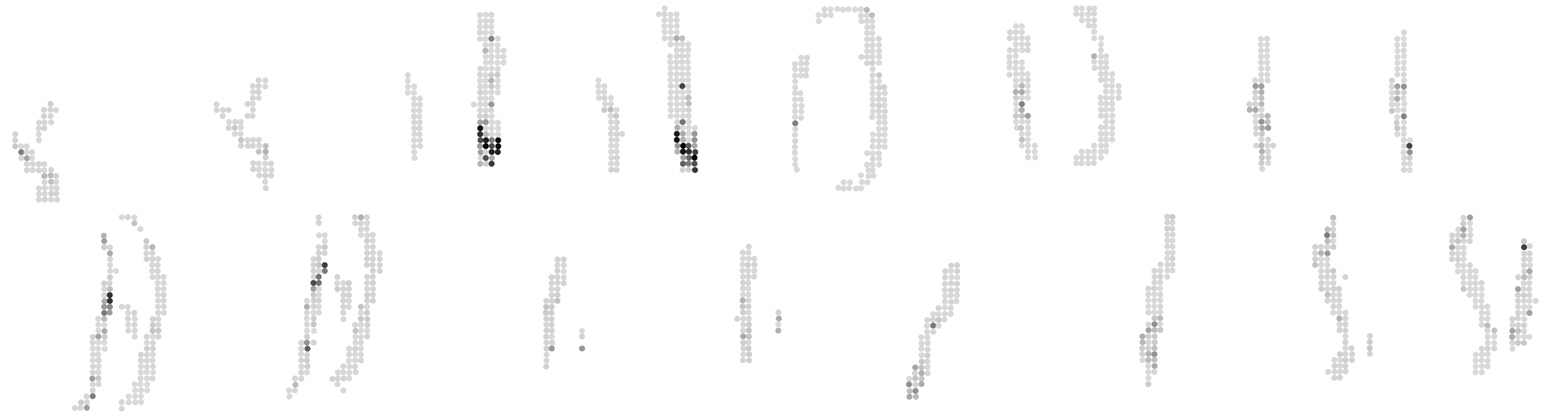

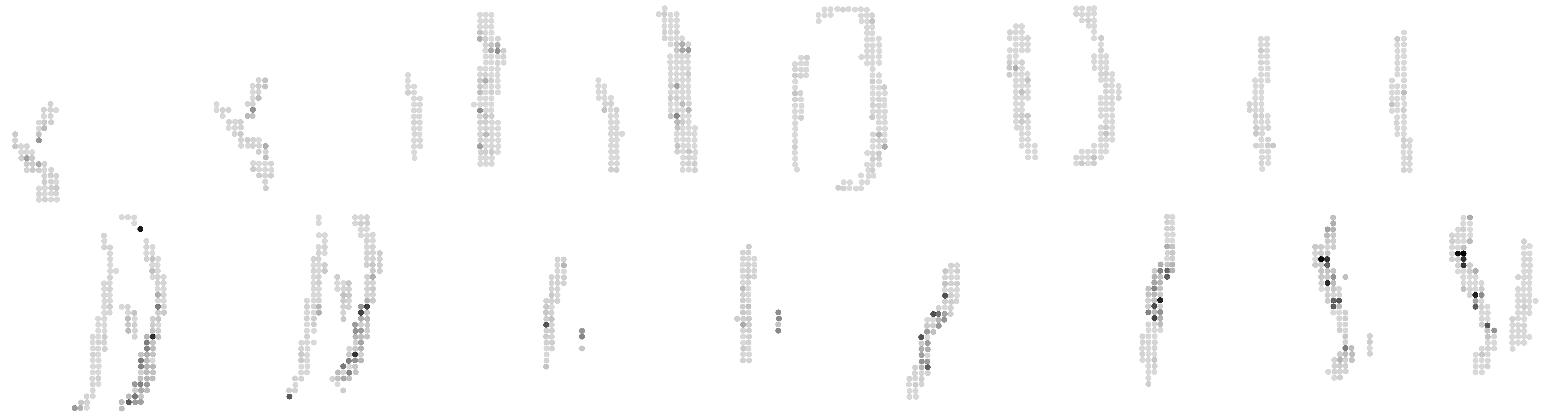

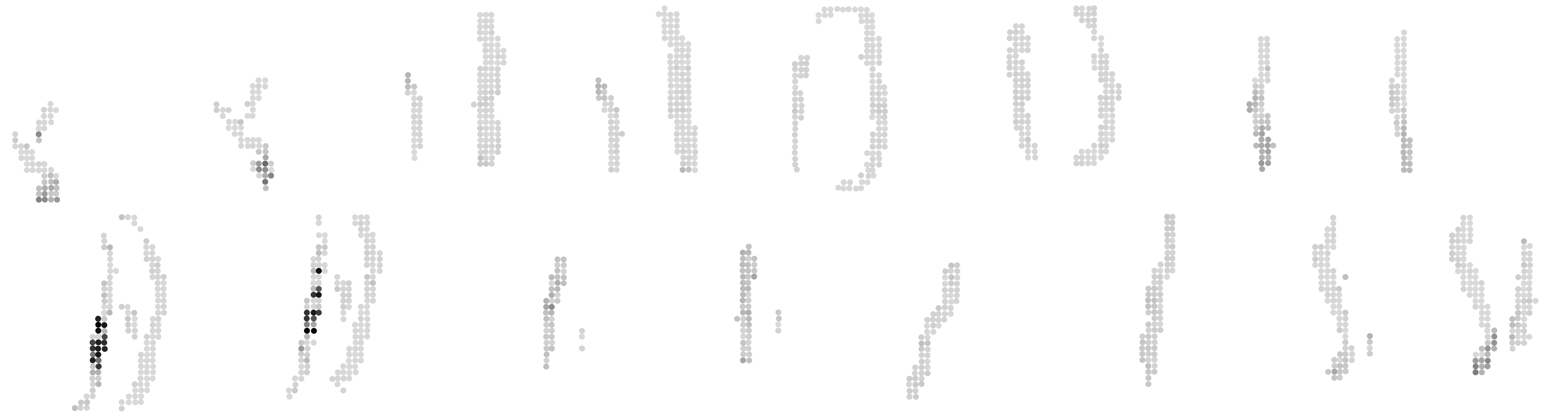

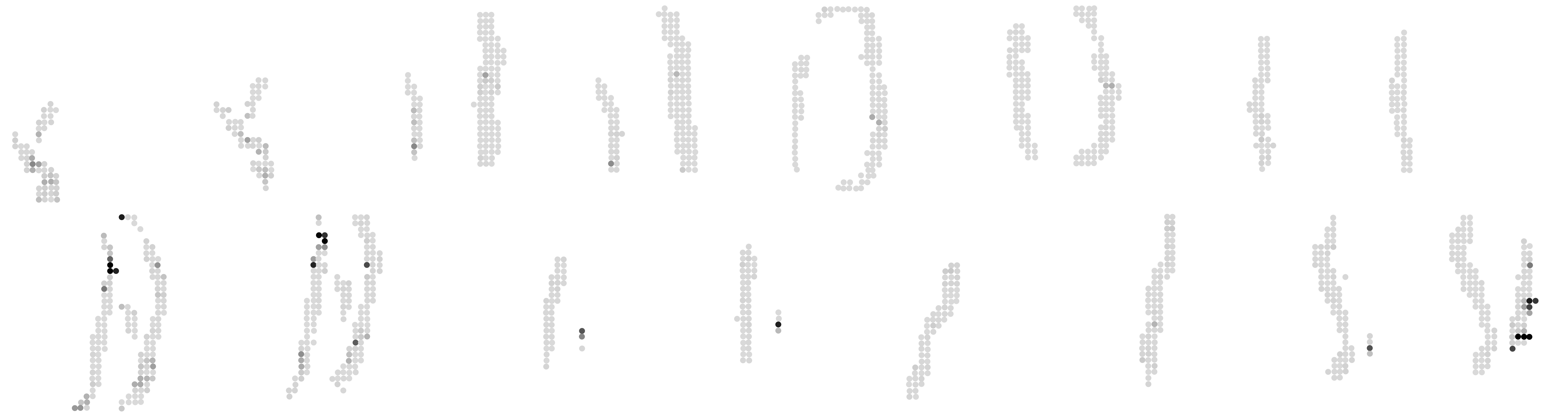

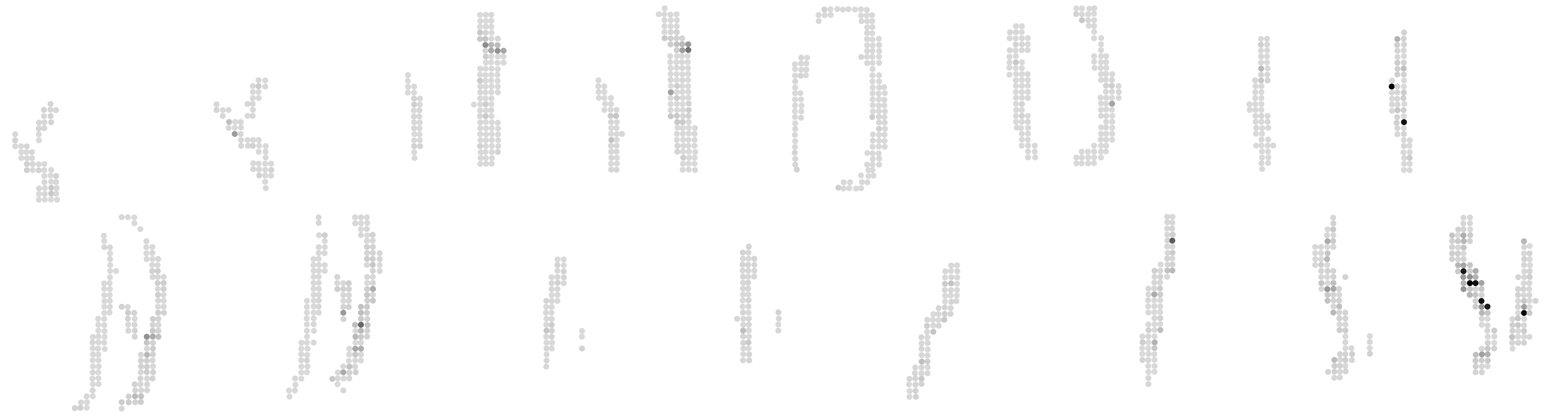

Supplement: Supplementary file 4 — Supplementary Dataset [file 41467_2022_33069_MOESM4_ESM.zip › Supplementary Data Files Nature Communications (Marklund et al. 2022)/Supplementary_Data_File3_STD Patient2_ActivityMap_16_Factors.pdf]
