# Supplementary material for: Spatio-temporal analysis of prostate tumors in situ suggests pre-existence of treatment-resistant clones
Source: Nat Commun. 2022 Sep 17;13:5475. doi: 10.1038/s41467-022-33069-3 (PMC9482614; doi:10.1038/s41467-022-33069-3)
Supplement: Supplementary file 5 — Reporting Summary [file 41467_2022_33069_MOESM5_ESM.pdf]

nature portfolio

Corresponding author(s): Joakim Lundeberg, Erik Sonnhammar

Last updated by author(s): Jul 25, 2022

Reporting Summary

Nature Portfolio wishes to improve the reproducibility of the work that we publish. This form provides structure for consistency and transparency in reporting. For further information on Nature Portfolio policies, see our Editorial Policies and the Editorial Policy Checklist.

Statistics

For all statistical analyses, confirm that the following items are present in the figure legend, table legend, main text, or Methods section.

n/a	Confirmed	
☐	☒	The exact sample size (*n*) for each experimental group/condition, given as a discrete number and unit of measurement
☐	☒	A statement on whether measurements were taken from distinct samples or whether the same sample was measured repeatedly
☐	☒	The statistical test(s) used AND whether they are one- or two-sided<br>*Only common tests should be described solely by name; describe more complex techniques in the Methods section.*
☒	☐	A description of all covariates tested
☐	☒	A description of any assumptions or corrections, such as tests of normality and adjustment for multiple comparisons
☐	☒	A full description of the statistical parameters including central tendency (e.g. means) or other basic estimates (e.g. regression coefficient) AND variation (e.g. standard deviation) or associated estimates of uncertainty (e.g. confidence intervals)
☐	☒	For null hypothesis testing, the test statistic (e.g. *F*, *t*, *r*) with confidence intervals, effect sizes, degrees of freedom and *P* value noted<br>*Give P values as exact values whenever suitable.*
☒	☐	For Bayesian analysis, information on the choice of priors and Markov chain Monte Carlo settings
☒	☐	For hierarchical and complex designs, identification of the appropriate level for tests and full reporting of outcomes
☒	☐	Estimates of effect sizes (e.g. Cohen's *d*, Pearson's *r*), indicating how they were calculated

Our web collection on statistics for biologists contains articles on many of the points above.

Software and code

Policy information about availability of computer code

Data collection

No software was used

Data analysis

-R version 3.6.1
-ST Pipeline version 0.8.3, Navarro et al. 2017
-STAR (2.5.0b)
-Spatial transcriptome decomposition: STDnxt (GNU General Public License v3 at https://github.com/SpatialTranscriptomicsResearch/std-nb/tree/5ed3523
-HTseq-count (version 0.11.3)
-R-package STUtility using R version 3.6.1
-R-package vegan (version 2.5.6)
-R-package STUtility
-R-package Stats (version 3.6.3)
-R-package DESeq2 (version 1.6.3)
-umap-learn, python (version 0.1.5)
-Pathway analysis using PathwAX.sbc.su.se

For manuscripts utilizing custom algorithms or software that are central to the research but not yet described in published literature, software must be made available to editors and reviewers. We strongly encourage code deposition in a community repository (e.g. GitHub). See the Nature Portfolio guidelines for submitting code & software for further information.

Data

Policy information about availability of data

All manuscripts must include a data availability statement. This statement should provide the following information, where applicable:

- Accession codes, unique identifiers, or web links for publicly available datasets
- A description of any restrictions on data availability
- For clinical datasets or third party data, please ensure that the statement adheres to our policy

Sequence raw data
Spatial transcriptome sequencing data from prostate cancer needle biopsies that support the findings of this study have been deposited at the European Genome-Phenome Archive (EGA, www.ebi.ac.uk/ega/), which is hosted by the European Bioinformatics Institute (EBI), under accession number EGAS00001006113 [https://ega-archive.org/studies/EGAS00001006113]. The data are available under Data Use Conditions (DUO) and are limited to non-for-profit use as well as health/medical/biomedical purposes. Access is granted if the above is fulfilled and local institutional review board/ethical review board approvals are provided.
ST and IHC experiments
Count matrices, high-resolution histological, and immunohistochemistry images are available on Mendeley:
Marklund, Maja (2022), "Prostate needle biopsies pre- and post-ADT: Count matrices, histological-, and Androgen receptor immunohistochemistry images", Mendeley Data, V1, doi: 10.17632/mdt8n2xgf4.1
[https://data.mendeley.com/datasets/mdt8n2xgf4/1]

Field-specific reporting

Please select the one below that is the best fit for your research. If you are not sure, read the appropriate sections before making your selection.

☒ Life sciences ☐ Behavioural & social sciences ☐ Ecological, evolutionary & environmental sciences

For a reference copy of the document with all sections, see nature.com/documents/nr-reporting-summary-flat.pdf

Life sciences study design

All studies must disclose on these points even when the disclosure is negative.

Sample size

For the 5 patients included in the paraffin embedded material, we wanted to see if cancer clusters, with AR receptors in the nuclei, occurred commonly after ADT. We argued that if we could see this in at least one of five patients we could consider it to not be a rare event. We have no intension to quantitatively decide the occurrence of these AR-positive clusters, only to say that they exist and that they are not rare events. We found the existence of the clusters in all five patient, making us confident to claim that they are not rare events.
For the 3 patients in the ST part we wanted to show that it was possible to localize these non responding clusters and describe their RNA profile, which we succeeded with. We do no claims on having the RNA profile for all, or not even the most, possible non responding variants. To do that a much larger number of patients is needed and lies beyond the scope of this study.

Data exclusions

All patients analysed were included in the data presented. There were no exluded subjects.

Replication

All biopsies used for ST-technology was done in two replicates. All replicates showed coherent results. The protocols for the immunostainings were developed on prostate needle biopsy tissues and extensively tested before using on the material included in this study.The experiments are based on sections from tissues, thus, perfect replicates is not possible since it will not be the same cells in different sections.

To be able to connect the protein-protein, or protein RNA levels in small cell clusters we need to have consecutive slides to not miss them. If we would have done the stainings in duplicate, the distance between the stainings of different protein would have increased and thus blurred the result. Therefore, we decided to develop the staining protocols and check for reproducibility on other biopsies, taken at the same time frame and treated in the same way (snap frozen, or ffpe).

Randomization

This study do not compare two different treatments. Thus randomization is not possible.

Blinding

In all biopsies, the annotation was made by pathologists who did not have any clinical information and in that sense it was blinded.

Identifying the nucleus and measuring the fluorescence signal within it in the biopsies was done with a script based on J-Image. Thus, no personal or manual judgment was made that could bias the data.

The same is true for the ST-procedure, no personal or manual judgment was made that could bias the data.

Reporting for specific materials, systems and methods

We require information from authors about some types of materials, experimental systems and methods used in many studies. Here, indicate whether each material, system or method listed is relevant to your study. If you are not sure if a list item applies to your research, read the appropriate section before selecting a response.

Materials & experimental systems

n/a	Involved in the study
☐	☒ Antibodies
☒	☐ Eukaryotic cell lines
☒	☐ Palaeontology and archaeology
☒	☐ Animals and other organisms
☐	☒ Human research participants
☒	☐ Clinical data
☒	☐ Dual use research of concern

Methods

n/a	Involved in the study
☒	☐ ChIP-seq
☒	☐ Flow cytometry
☒	☐ MRI-based neuroimaging

Antibodies

Antibodies used

Primary antibodies used: AR antibody:(N-20, Catalog # cs 816, SCBT, 1:500), primary chromogranin A antibody: (LK2H10, Catalog # MA5-13096, Invitrogen, 1:150), Ku70 antibody: (E-5, Catalog # sc-17789, SCBT, 1:500), phosphorylated DNA-PKcs:(S2056, Catalog# ab18192, Abcam, 1:750)

Secondary antibodies used: Donkey anti-Rabbit IgG (H+L) Highly Cross-Adsorbed Secondary Antibody, Alexa Fluor™ 647: (Cat. # A-31573, 1:500), Donkey anti-Rabbit IgG (H+L) Highly Cross-Adsorbed Secondary Antibody, Alexa Fluor™ 555:(Cat. # A-31572, 1:500), Donkey anti-Mouse IgG (H+L) Highly Cross-Adsorbed, Alexa Fluor™ 488 (Cat. # A-21202, 1:500) and Donkey anti-Mouse IgG (H+L) Highly Cross-Adsorbed Secondary Antibody, Alexa Fluor™ 555: (Cat. #A-31570 , 1:500). All secondary antibodies were from ThermoFisher scientific, Molecular Probes.

Validation

N-20 has been validated in several applications, including in paraffin embedded tissue and in immunofluorescence in prostate cells and tissues by the manufacturer. Further, a numerous articles have been using this antibody successfully in those applications in prostate research. We have also used it successfully earlier in another prostate study (Science Translational Medicine, 4 November, 2015, Vol 7, Issue 312).

The Chromogranin A antibody (LK2H10) have been validated in several applications, including Immunofluorescence in formalin fixated neuroendocrine positive cells, by the manufacturer. Further, it has been used successfully to show that EGF promotes neuroendocrine-like differentiation of prostate cancer cells in the presence of LY294002 (Carcinogenesis, 2012 Jun;33(6):1169-77)

Human research participants

Policy information about studies involving human research participants

Population characteristics

Patient	Age	Diagnosis	GS	Treatment	Days	PSA *pre	PSA *post	Testosterone *post
1	77	Cancer, T3	4+5	GnRH-analogue	56	41	0.17	0.52
2	76	Cancer, T4	4+4	GnRH-analogue	56	10780	2.1	0.64
3	74	Cancer, T3	4+5	GnRH-analogue	56	65	17	0.84
A	62	Cancer, T3	3+3	GnRH-analogue	67	4	0.12	0.87
B	62	Cancer, T3	3+4	GnRH-analogue	59	19	1.3	0.0
C	60	Cancer, T4	4+3	GnRH-analogue	112	19	2.4	0.0
D	65	Cancer, T3	4+5	GnRH-analogue	54	95	11	0.78
E	71	Cancer, T3	4+5	GnRH-analogue	54	59	18	0.43

Biopsies pre and post treatment from Patient 1-3 were investigated with immunohistochemistry and spatial transcriptomics, while biopsies from patients A-E were investigated with immumohistochemistry only.

*PSA was measured as ng/mL and testosterone as nmol/L

Recruitment

Participants were identified by one of the study pathologists. All participants were provided with full and adequate verbal and written information about the study before their participation. Written informed consent was obtained from all participants before enrollment in the study.

Patients included for the ffpe study were patients with naive prostate cancer. We included all that had agreed to participate.

Patient included for ST were patients with naive prostate cancer selected by the severeness of the cancer based on the PSA response of the ADT treatment; good responder, intermediate responder and poor responder. No other selection was made.

We have hard to see any self-selection bias in these criteria.

No participant compensation was given.

Ethics oversight

The study was performed according to the Declaration of Helsinki, Basel Declaration and Good Clinical Practice. The study was approved by the Regional Ethical Review Board (REPN) Uppsala, Sweden before study initiation (Dnr 2011/066/2, Landstinget Västmanland, Sari Stenius).

Note that full information on the approval of the study protocol must also be provided in the manuscript.
